# Infant Formula Consumption Is Positively Correlated with Wealth, Within and Between Countries: A Multi-Country Study

**DOI:** 10.1093/jn/nxz327

**Published:** 2019-12-25

**Authors:** Paulo A R Neves, Giovanna Gatica-Domínguez, Nigel C Rollins, Ellen Piwoz, Phillip Baker, Aluísio J D Barros, Cesar G Victora

**Affiliations:** 1 International Center for Equity in Health, Postgraduate Program in Epidemiology, Federal University of Pelotas, Pelotas, Rio Grande do Sul State, Brazil; 2 Department of Maternal, Newborn, Child and Adolescent Health, WHO, Geneva, Switzerland; 3 Global Development Program, The Bill & Melinda Gates Foundation, Seattle, WA, USA; 4 Institute for Physical Activity and Nutrition, Deakin University, Burwood, Victoria, Australia

**Keywords:** breast feeding, infant and young child feeding, breastmilk substitutes, socioeconomic factors, economic status, health equity

## Abstract

**Background:**

In contrast with the ample literature on within- and between-country inequalities in breastfeeding practices, there are no multi-country analyses of socioeconomic disparities in breastmilk substitute (BMS) consumption in low- and middle-income countries (LMICs).

**Objective:**

This study aimed to investigate between- and within-country socioeconomic inequalities in breastfeeding and BMS consumption in LMICs.

**Methods:**

We examined data from the Demographic Health Surveys and Multiple Indicator Cluster Surveys conducted in 90 LMICs since 2010 to calculate Pearson correlation coefficients between infant feeding indicators and per capita gross domestic product (GDP). Within-country inequalities in exclusive breastfeeding, intake of formula or other types of nonhuman milk (cow/goat) were studied for infants aged 0–5 mo, and for continued breastfeeding at ages 12–15 mo through graphical presentation of coverage wealth quintiles.

**Results:**

Between-country analyses showed that log GDP was inversely correlated with exclusive (*r* = −0.37, *P* < 0.001) and continued breastfeeding (*r* = −0.74, *P* < 0.0001), and was positively correlated with formula intake (*r *= 0.70, *P* < 0.0001). Continued breastfeeding was inversely correlated with formula (*r* = −0.79, *P* < 0.0001), and was less strongly correlated with the intake of other types of nonhuman milk (*r* = −0.40, *P* < 0.001). Within-country analyses showed that 69 out of 89 did not have significant disparities in exclusive breastfeeding. Continued breastfeeding was significantly higher in children belonging to the poorest 20% of households compared with the wealthiest 20% in 40 countries (by ∼30 percentage points on average), whereas formula feeding was more common in the wealthiest group in 59 countries.

**Conclusions:**

BMS intake is positively associated with GDP and negatively associated with continued breastfeeding in LMICs. In most countries, BMS intake is positively associated with family wealth, and will likely become more widespread as countries develop. Urgent action is needed to protect, promote, and support breastfeeding in all income groups and to reduce the intake of BMS, in light of the hazards associated with their use.

## Introduction

Breastfeeding is of crucial importance for individuals and nations, and its lifelong benefits are well established for both mother and child. For the latter, breastfeeding protects against infections and death in childhood, increases child and adult intelligence, and likely reduces the risk of overweight and diabetes in adulthood. For mothers, breastfeeding protects against ovarian and breast cancer, and increases interbirth intervals (1–4). In spite of its health benefits, breastfeeding practices in most, if not all countries, are suboptimal in relation to international recommendations: for example, only 37% of children under 6 mo of age were exclusively breastfed and fewer than three-quarters of children aged 12–15 mo were still being breastfed in low- and middle-income countries (LMICs) in 2015 (2). Barriers to optimal breastfeeding occur at multiple levels and include lack of enabling policies and programs at national level, poor support from health workers, aggressive marketing of breastmilk substitutes (BMSs), short maternity leave, and individual-level decisions (3).

Between-country inequalities in breastfeeding patterns were explored in *The Lancet*’s 2016 Breastfeeding Series. The total duration of any breastfeeding tends to be longer in low-income, intermediate in middle-income, and shorter in high-income countries. Data on exclusive breastfeeding are not routinely reported by most high-income countries, but low-income countries also tend to have higher rates than middle-income countries (2). This finding is supported by data on per-child BMS sales, which are noticeably higher in high-income countries than in LMICs (5). *The Lancet* series also reported on an analysis of within-country socioeconomic inequalities in 126 countries, and found minor inequalities in exclusive breastfeeding, in contrast to pronounced propoor inequalities (higher prevalence among poorer quintiles) for continued breastfeeding at age 1 or 2 y (2, 3). A possible explanation for these patterns is the extensive consumption of BMS, in particular formula, among children from wealthier families (3). There is concern that poor countries and families may move towards higher BMS consumption as their incomes increase, and as formula feeding becomes increasingly perceived as sophisticated and modern, whereas breastfeeding is regarded as primitive and old-fashioned (2, 3).

In the present analyses, we used national survey data to describe within-country and between-country inequalities in breastfeeding and BMS consumption. Inequalities in breastfeeding have been previously described (2, 6), however, we were unable to find any published analyses on between-country inequalities in BMS consumption (as opposed to formula sales) or on within country inequalities in BMS consumption, covering LMIC populations.

Understanding the socioeconomic barriers to optimal breastfeeding practices is essential for policymakers, program managers, and stakeholders engaged in the promotion, protection, and support of optimal breastfeeding. Recognizing these drivers can help to inform actions to achieve the World Health Assembly's Global Nutrition Target of increasing the worldwide rate of exclusive breastfeeding in the first 6 mo of life to 50% by 2025, and Sustainable Development Goal 2 Target 2.2. on eliminating all forms of malnutrition by 2030. Using data from 90 LMICs with a national survey from 2010 to 2017, we report on between-country and within-country socioeconomic inequalities in the prevalence of breastfeeding and BMS consumption (including formula and other nonhuman milk).

## Methods

Our analyses are based on nationally representative studies carried out in LMICs, including Demographic Health Surveys (DHS) (7) and Multiple Indicator Cluster Surveys (MICS) (8). Both surveys are cross-sectional household studies covering a large number of reproductive, maternal, newborn, and child health and nutrition indicators, using standardized questionnaires administered via face-to-face interviews by trained fieldworkers with women of childbearing age (15–49 y) and the caregivers of children under the age of 5 y. Data on infant and young child feeding (IYCF) practices relied upon 24-h recall. The 2 types of surveys are highly comparable in terms of sampling methods, questionnaires, measurements, and field procedures (9). We also included data from the nationally representative *Encuesta Nacional de Salud y Nutrición* (National Health and Nutrition Survey) conducted in Ecuador in 2012, after harmonizing its dataset with indicators obtained from DHS/MICS surveys (10).

The International Center for Equity in Health database contains >350 publicly available DHS and MICS conducted from 1991 onwards in LMICs, for which data harmonization was carried out. Data from 2010 to 2017 were available for 100 surveys by the date of analysis. **Supplemental Figure 1** shows that between 87–90 surveys contained information on the indicators of interest and sufficient sample size for analyses.

IYCF indicators were calculated according to WHO definitions (11, 12), when a definition was available. These included: exclusive breastfeeding under 6 mo (proportion of infants aged 0–5 mo who were fed exclusively with breast milk) and continued breastfeeding at 1 y (proportion of children 12–15 mo of age who were fed breast milk). We also calculated prevalence of formula consumption under 6 mo (proportion of infants aged 0–5 mo who were fed formula), and prevalence of consumption of nonhuman milk other than formula (e.g. cow or goat milk) for children under 6 mo (proportion of infants aged 0–5 mo who were fed nonhuman milk other than formula). In addition, we calculated the percentage of children who received formula among those who received any type of BMS (either formula, other types of milk, or both) to show formula as a proportion of total BMS consumption. We considered the last born if a woman had multiple births in the reference period. The denominator was the number of last born children within the age range surveyed (11, 12). National estimates for exclusive and continued breastfeeding were compared with published DHS and MICS national reports; all differences between our recalculated estimates and those presented in the national report were within <1% point, except for small discrepancies in exclusive breastfeeding, mostly occurring when some food groups were not taken into account to generate the estimates in the report. Missing values and “don't know” answers for liquid and food intake were considered as “not consumed”, as is standard practice in the international literature (13, 14).

Our between-country, ecological analyses include the correlation between national-level estimates of feeding indicators and gross national domestic product (GDP) per capita (with power purchasing parity in constant 2011 international dollars) obtained from the World Bank database (15). GDP was log-transformed to improve linear fit. Pearson and partial correlation coefficients were calculated for national prevalence of breastfeeding indicators and consumption of BMSs; correlations were calculated using countries as the units of analyses. We graphed scatter plots to illustrate the relation between breastfeeding indicators with formula by income groups. Linear regression was used to estimate the effect of doubling the GDP per capita on formula consumption prevalence. Departures from linearity were explored with fractional polynomials, showing that the linear regression with log GDP provided an appropriate fit to the data.

The wealth index provided with the survey datasets was used to analyze within-country socioeconomic inequalities. The index was calculated through principal component analysis for each survey based on the ownership of assets and building characteristics of each household (16, 17). As the presence of relevant assets and access to electricity, sanitation, and water may vary in urban and rural households, separate principal component analyses were carried out for each area, then later combined into a single score using a scaling procedure to allow comparability between urban and rural households (18). The definition of area of residence is based on country-specific definitions (18). The resulting score was then split into quintiles, with the first quintile representing the poorest 20% of all families and the fifth quintile representing the wealthiest 20% of all families (19).

We calculated measures of absolute and relative inequalities to examine wealth-related discrepancies. The slope index of inequality expresses absolute inequalities, being typically derived through a logistic regression model where the outcome is the prevalence for each feeding indicator and the explanatory variable is a fractional rank based on the wealth index. The index represents the absolute difference in the fitted value of the health indicator between the highest and the lowest values of the socioeconomic indicator rank (19). The index is expressed in percentage points since all our indicators are prevalence. Relative inequality was assessed through the concentration index, which uses an analogous approach to the Gini index, by ranking individuals according to socioeconomic position on the x-axis and plotting cumulative prevalence of the outcomes on the y-axis (19). Both indices were expressed on a scale from −100 to +100, with zero representing no inequalities across the wealth scale; positive values represent a prorich distribution (higher prevalence among richer quintiles) and negative values a propoor distribution (20).

Country-level analyses accounted for the multi-stage survey design, including sampling weights and clustering. Databases were handled using Stata 15.0 (StataCorp.) and Microsoft Excel^®^ spreadsheets (Microsoft Corp.). Estimates for countries and wealth quintiles are presented alongside their 95% CI for each indicator.

Countries were grouped according to UNICEF regions and World Bank income groups on the year of the survey (21, 22). **Supplemental Table 1** provides a list of countries in each group, as well as the survey sample sizes by age range. Regional and income group estimates were weighted by the size of the population of children within the respective age ranges retrieved from World Bank Population Estimates and Projections, in the year when the survey was carried out (23). In order to visualize socioeconomic inequalities among regions and income groups we used equiplots, which includes a horizontal line to link dots that represent the wealth quintiles (http://www.equidade.org).

We used publicly available data, and the ethical clearance for conducting the surveys was the responsibility of the national institutions that were in charge of data collection.

## Results

Between 87 and 90 countries were included in the analysis, depending on the outcome (Supplemental Table 1, Supplemental Figure 1). The median number of children aged 0–5 and aged 12–15 mo was 703 (ranging from 145 in Kosovo to 22,626 in India) and 526 (ranging from 99 in Kosovo to 16,237 in India), respectively. The median year of the surveys was 2014, the earliest dating from 2010 (Bhutan, Burkina Faso, South Sudan, Suriname, and the Central African Republic) and the latest from 2017 (Albania, Jordan, Philippines, Senegal, and Tajikistan). Data were available for 93.5% of all low-income countries, 70.6% of lower-middle-income countries, and 52.8% of upper-middle-income countries, as of 2014.

### Between-country analysis

#### National-level correlations

**FIGURE 1:**
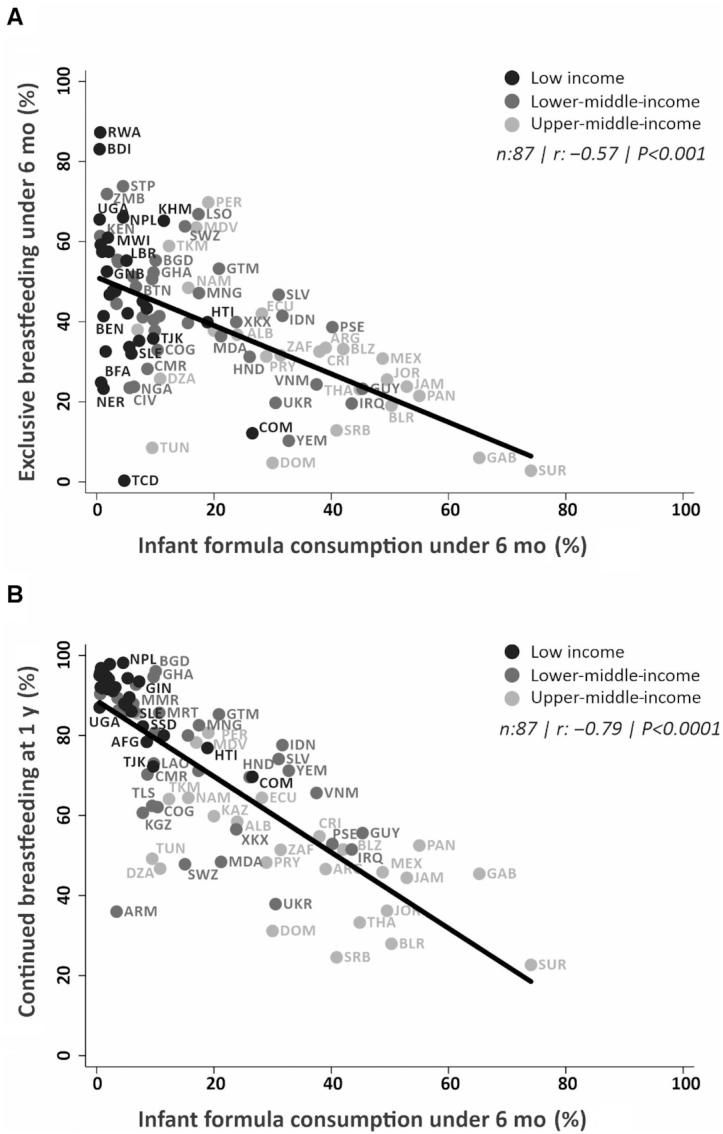
Pearson's correlation between national prevalence of exclusive breastfeeding under 6 mo (A) and continued breastfeeding at 1 y (B) with formula consumption under 6 mo of age for 87 countries with available household surveys from 2010–2017 by income groups. *n*, number of countries; *r*, coefficient of correlation.

**Table 1** shows the results of ecological analyses with each country as the units. GDP per capita was negatively correlated with exclusive breastfeeding and continued breastfeeding at 1 y, and positively correlated with formula and other types of nonhuman milk consumption. The regression analysis showed that formula consumption increased by 7 percentage points for every 2-fold increase in GDP per capita. Exclusive breastfeeding was inversely correlated with formula (**Figure 1**) and other nonhuman milk consumption, whereas continued breastfeeding at 1 y was strongly and inversely correlated with formula consumption (Figure 1) and moderately correlated with the consumption of other types of milk in the first 6 mo.

**TABLE 1:** Pearson's correlation matrix for indicators of breastfeeding and breastmilk substitutes, and country-specific log per capita gross domestic product for countries with available household survey from 2010–2017^1^

	Log GDP^2^	Exclusive breastfeeding under 6 mo	Infant formula consumption under 6 mo	Consumption of nonhuman milk other than formula under 6 mo	Continued breastfeeding at 1 y
Log GDP^2^	1.00	—	—	—	—
Exclusive breastfeeding under 6 mo	−0.37**	1.00	—	—	—
Infant formula consumption under 6 mo	0.70***	−0.57**	1.00	—	—
Consumption of nonhuman milk other than formula under 6 mo	0.25*	−0.46***	0.26**	1.00	—
Continued breastfeeding at 1 y	−0.74***	0.56***	−0.79***	−0.40**	1.00

1 *P* level: *P < 0.01; **P < 0.001; ***P < 0.0001.

2 Log GDP, log-transformed gross domestic product per capita, power purchasing parity (constant 2011 international dollars).

After adjustment for nonhuman milk, the partial correlation coefficients between breastfeeding indicators and formula were equal to −0.53 (*P *< 0.0001) for exclusive breastfeeding and −0.77 (*P *< 0.0001) for continued breastfeeding at 1 y. The coefficients for other nonhuman milk, adjusted for formula, were −0.40 (*P *= 0.0001) and −0.36 (*P *= 0.0007) for exclusive and continued breastfeeding, respectively.

### Within-country analysis

#### Exclusive breastfeeding under 6 mo

**FIGURE 2:**
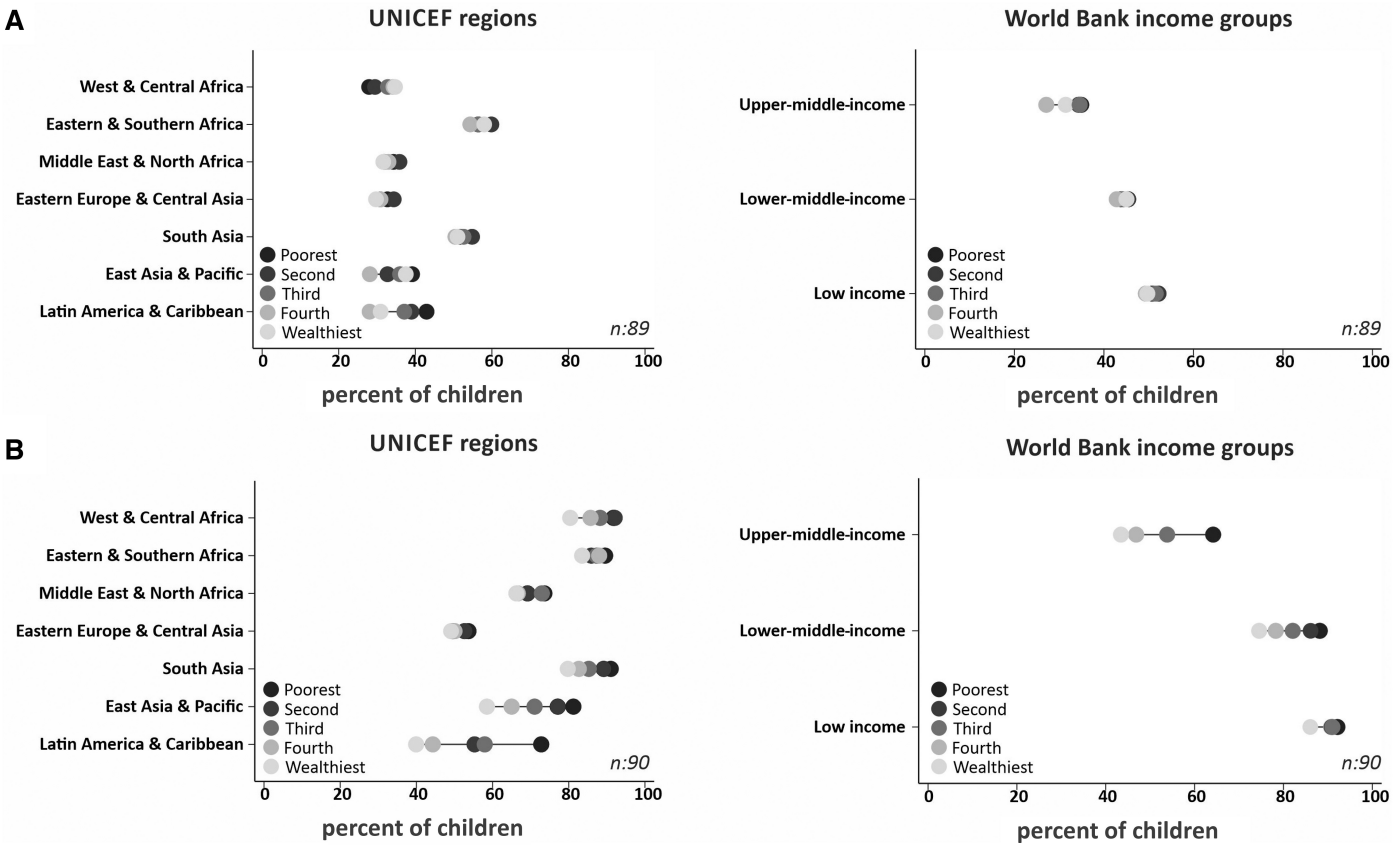
Average weighted national prevalence of exclusive breastfeeding under 6 mo (A) and continued breastfeeding at 1 y (B) by wealth quintiles, grouped by world region and income for countries with available household surveys from 2010–2017. *n*, number of countries.

**Supplemental Table 2** shows the proportion of infants below 6 mo who were exclusively breastfed at the time of the survey at national level and by wealth quintiles, as well as the corresponding national slope and concentration indices. Prevalence varied from 0.3% in Chad to 87.3% in Rwanda. The mean value of the slope index was −3.9% points, indicating higher prevalence among children from poor rather than rich families; 21 countries had values significantly different from zero for the index, 7 with a higher prevalence among the rich, and 14 with a higher prevalence among poor children. Cameroon (slope index of 30.9) had the most pronounced prorich distribution, whereas Guatemala (−58.8) the most marked propoor pattern. The average concentration index for all countries was −1.8, suggesting an overall propoor pattern with 25 values significantly different from zero, of which 9 were prorich and 16 were propoor (Supplemental Table 2). When countries were grouped by world region (Figure 1A; **Supplemental Table 3**), only Latin America and the Caribbean showed clear inequalities with higher prevalence among the poor. In terms of World Bank groups (**Figure 2**; Supplemental Table 3), there were no salient patterns, with the exception of lower prevalence in the 2 wealthiest quintiles compared with other quintiles in upper-middle-income countries.

#### Continued breastfeeding at 1 y

**Supplemental Table 4** presents results for continued breastfeeding at 1 y. The lowest prevalence was in Suriname (22.7%) and the highest in Nepal (98.1%). The mean value for the slope index was −17.0% points, showing a propoor distribution; the index was significantly negative for 40 countries, and no country had a prorich distribution. Cameroon displayed the highest significant value for the slope index (−60.2) and Guinea the lowest (−13.1). In consonance with the slope index, the mean concentration index was −4.9, with 41 countries showing significant negative values. Costa Rica had the highest concentration index (−18.6) and Burundi (−1.1) the lowest (Supplemental Table 4). In all regions analyzed, marked propoor inequalities were observed, with monotonic associations in most regions. In Latin America and the Caribbean prevalence ranged from 40% in the richest to over 70% in the poorest quintile (Figure 2; **Supplemental Table 5**). For country income groups, the widest disparities were seen in upper-middle-income countries, where prevalence among the poorest children was noticeably higher than in the other 4 quintiles (Figure 1B; Supplemental Table 5).

#### Infant formula consumption under 6 mo

**FIGURE 3:**
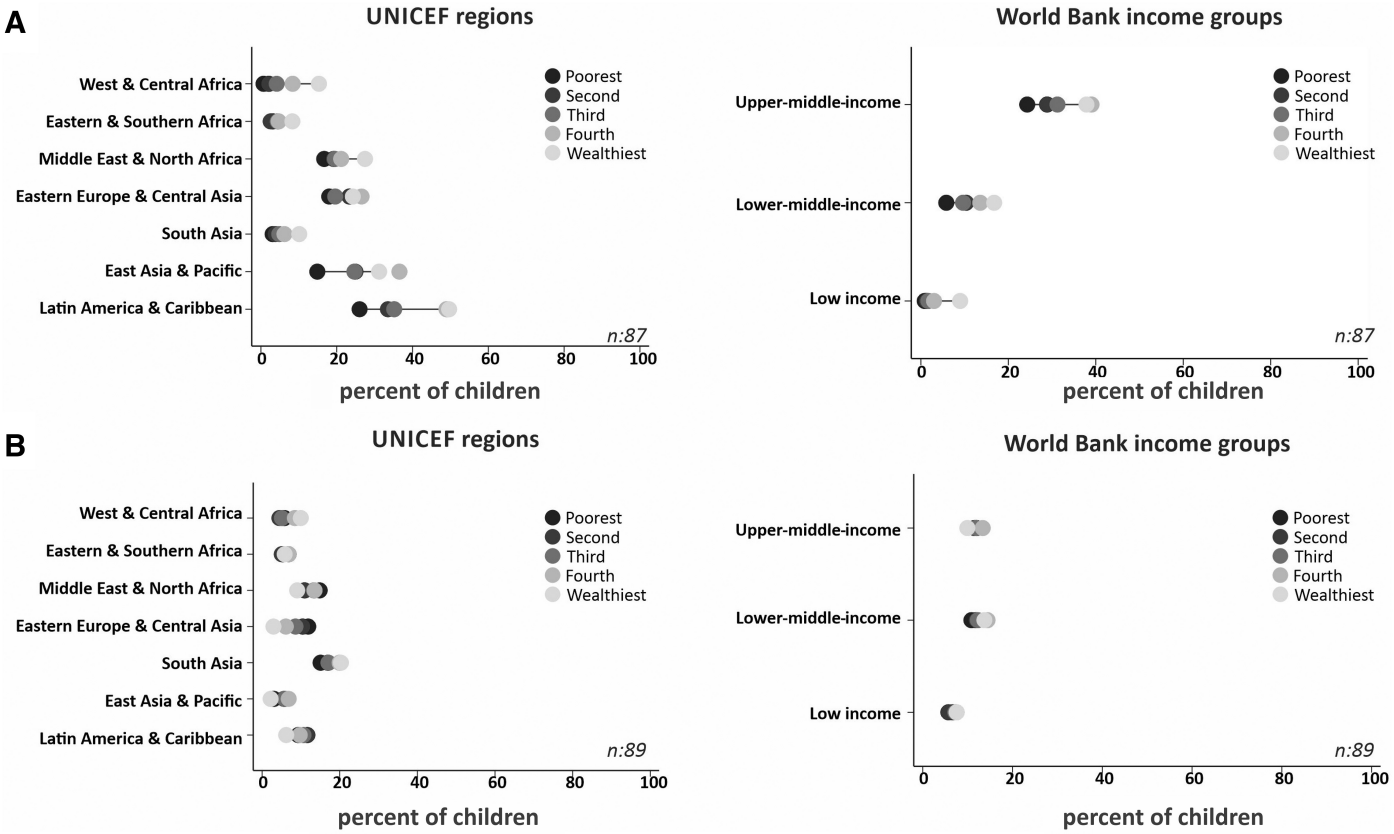
Average weighted national prevalence of infant formula consumption under 6 mo of age (A) and consumption of other nonhuman milk under 6 mo (B) by wealth quintiles, grouped by world region and income for countries with available household surveys from 2010–2017. *n*, number of countries.

As shown in **Supplemental Table 6** the prevalence of formula consumption ranged from 0.5% in Burundi to 74.1% in Suriname. All 59 countries with significant slope indices showed prorich patterns, with a mean value of 17.9% points, ranging from 71.3 in Panama to 2.1 in Rwanda. Accordingly, 67 countries had significant positive values for the concentration index, with a mean value of 29.9. The only country with a significant propoor distribution was Armenia where the index was −36.5 (Supplemental Table 6). Prorich distributions were observed in all regions of the world, particularly in Latin America and the Caribbean where formula feeding was more common than in other regions; even the poorest quintile in this region had a higher prevalence than in the wealthiest quintile in West and Central Africa, Eastern and Central Africa, and South Asia. Less than 1% of the infants in the poorest quintile in West and Central Africa consumed formula (**Figure 3**; **Supplemental Table 7**). Prorich patterns were also observed in the analyses according to country income groups, particularly among upper-middle-income countries (Figure 3; Supplemental Table 7).

#### Consumption of nonhuman milk other than formula under 6 mo

**Supplemental Table 8** shows that national prevalence of consumption of nonhuman milk other than formula under 6 mo ranged from 0.9% in Togo to 60.1% in the Dominican Republic. Significant slope indices were observed in 31 countries, of which 25 displayed higher prevalence among wealthier children, and only 6 among poor children. The most marked prorich pattern was observed in Guyana (27.5), whereas the sharpest propoor pattern was found in Argentina (−21.4). Likewise, 30 countries had significant positive values and 12 had significant negative values for the concentration index, ranging from 73.1 in Zimbabwe to −55.3 in Eswatini (Supplemental Table 8). The analyses by world regions and country income groupings failed to show any clear patterns (Figure 3; **Supplemental Table 9**).

We analyzed the share of formula consumption among infants aged 0–5 mo who received any type of nonhuman milk. **Supplemental Table 10** shows the results for each country. The average formula share across the 87 countries were 44%, 50%, 55%, 60%, and 74% from the poorest to the wealthiest quintile, respectively, demonstrating that, among children who received BMS, formula became progressively more common as family income increased.

## Discussion

We investigated socioeconomic inequalities in breastfeeding and BMS consumption in 90 countries, using data from surveys carried out between 2010 and 2017. An earlier multi-country comparison of inequalities in breastfeeding practices published in 2016 covering 126 countries up to 2014, showed a lack of clear social gradients in exclusive breastfeeding, in contrast to a strong propoor pattern in the prevalence of continued breastfeeding at 1 y (2). The latter finding was confirmed by a UNICEF report on inequalities in 73 countries with surveys from 2010 to 2016 (6). Neither of these publications reported upon inequalities in BMS consumption.

The results of this within-country analysis also shows a lack of clear socioeconomic inequalities in exclusive breastfeeding in all regions except for Latin America and the Caribbean, where this practice was more common among children from poor families. There was no evidence of absolute inequality in 68 of the 89 countries studied, with a tendency towards more propoor distributions in those countries with significant inequality. Our results also confirm the propoor pattern in the prevalence of continued breastfeeding at 1 y in all regions, particularly in Latin America and the Caribbean, and East Asia and Pacific. Of the 90 countries, 40 had significant propoor distributions and none showed prorich patterns in breastfeeding continuation.

Our within-country analyses on BMS consumption revealed prorich distributions in formula consumption in all countries and regions, whereas nonhuman milk consumption did not show any clear socioeconomic patterns in most regions, except for Eastern Europe and Central Asia where there was a discrete propoor distribution. At country level, 58 and 47 out of 89 countries did not show absolute and relative inequalities in nonhuman milk consumption, respectively.

Analyses by World Bank country income groups failed to show clear within-country inequalities in exclusive breastfeeding. However, the results for continued breastfeeding and BMS consumption are reasons for concern. There was little inequality and a high prevalence of breastfeeding at 12–15 mo in low-income countries. In these countries, formula consumption under 6 mo was restricted to the richest quintile—albeit at a low prevalence. In contrast, upper-middle-income countries showed important propoor gaps in continued breastfeeding and prorich gaps in formula consumption. Nonhuman milk consumption, on the other hand, did not show clear patterns in inequalities or prevalence among country income groups. These results suggest that, as countries become richer, breastfeeding is replaced with formula feeding, initially among children from wealthier families, followed by the rest of the population.

Our findings of within-country inequalities in breastfeeding in LMICs sharply contrasts with patterns observed in high-income settings, where breastfeeding tends to be more common and lasts longer among children born to more educated, wealthier mothers (2, 24–26). Of the 25 upper-middle-income countries in our analyses, only 3 (Argentina, Thailand, and Serbia) showed some indication of higher exclusive breastfeeding in the top quintile, and another 2 (Jordan and Kazakhstan) of higher prevalence of breastfeeding at 1 y among better-off children.

Between-country comparisons showed that formula sales are markedly greater in high-income countries than in LMICs (3, 5), which is consistent with the short duration of breastfeeding in industrialized countries (2). Even within LMICs, our analyses showed that per capita GDP was strongly and directly correlated with the use of formula and nonhuman milk, and inversely correlated with both breastfeeding indicators. Even more striking is our finding of a strong inverse correlation between early formula introduction and the prevalence of breastfeeding at 1 y. These ecological-level findings are in agreement with individual-level studies, including randomized trials, showing that the introduction of formula, formula advertising, and provision of free samples in hospitals negatively affects breastfeeding initiation and duration (27–29).

Our results reinforce the conclusions of the 2016 *Lancet* Breastfeeding Series, namely that the marketing and distribution of infant formula represent a major threat to optimal breastfeeding practices, as countries become richer and families are able to afford BMS alongside a shift in social norms unfavorable to breastfeeding (3). According to the latest Euromonitor data, total world formula sales (for infants/children aged 0–36 mo) grew by 30.3% from 4.3 to 5.6 kg/infant or child in 2008–2013, outstripping GDP growth of 25.7% over the same period. Since then, total world formula sales has slowed, growing by 9.2% from 5.8 to 6.3 kg/infant or child in 2014–2018, although growth remains strong in many middle-income countries (30). This market expansion has applied not only to infant formula (0–5 mo) but also to follow-up (6–12 mo) and toddler (children aged 13–36 mo) formula categories, which can displace continued breastfeeding (5).

The scale and anticipated impacts of the transition towards higher formula diets is unprecedented, given the large infant and young child population sizes of transitioning countries (5). The drivers of this transition include income growth, urbanization, workforce feminization, the medicalization of pregnancy and childbirth, more intensive formula marketing practices, and the failure of policies to promote, protect, and support breastfeeding in these new contexts (3, 5). The social stratification of breastfeeding and formula consumption reported here suggests these drivers have variegated and dynamic effects within countries, initially affecting the feeding choices of higher-income groups, and at later stages reaching middle- and low-income groups as countries transition. However, further investigation is needed to understand the interactions between these drivers and their socially stratified effects within and between countries at different stages of transition.

Our analyses have some limitations. Whereas survey data were available for nearly all low-income countries, only half of all upper-middle-income countries had data for analyses. Of 97 countries with available information, 6 had to be excluded from the stratified analyses due to small sample sizes in some wealth quintiles. Although we only included surveys carried out between 2010 and 2017, 13% of the countries had data collected before 2012, and breastfeeding practices may have changed since then. In addition, lack of recent, standardized data for large countries such as Brazil and China, as well as for high-income countries, may have affected our results as these constitute important markets for formula at the global level.

The strengths of our analyses include the use of nationally representative data from a large number of LMICs, allowing the first description of socioeconomic disparities in the use of formula in the literature. The 2 types of surveys—DHS and MICS—are highly comparable in terms of sampling methodology and field procedures, and both used standardized definitions for infant feeding and the assessment of socioeconomic position in urban and rural areas. Feeding information was obtained through 24-h recall of an extensive list of foods and fluids, thus minimizing recall bias.

There is ample evidence on the hazards associated with formula feeding and suboptimal breastfeeding practices. These include not only increased child morbidity and mortality, but also reduced human capital in adulthood associated with lower intelligence, and possible effects on diabetes and obesity (2, 3, 31). In addition, breastfeeding is associated with a lower risk of breast and ovarian cancer for the mother, as well as with birth spacing (2). Our findings on the levels and disparities in breastfeeding and BMS consumption are causes for concern, due to the hazards associated with the lack of breastfeeding and to the increasing use of BMS in higher-income countries. Better-off women and families are often trendsetters within a society (32) and their growing adoption of formula feeding will likely influence the feeding decisions of women from low-income families, who are currently more likely to breastfeed. Urgent action is needed in order to promote and support exclusive breastfeeding in all social groups, and to protect the practice of breastfeeding in the second year among the poorest mothers and their children.

## Supplementary Material

nxz327_Supplemental_File

## Notes

This study was supported by The Bill & Melinda Gates Foundation, through the WHO (OPP1148933) and Associação Brasileira e Saúde Coletiva (Brazilian Association of Collective Health). PARN received scholarships from the Brazilian National Council for Scientific and Technological Development—CNPq (grant number 155541/2018-8). The funders had no role in the design and conduct of the study; collection, management, analysis, and interpretation of the data; preparation, review, and approval of the manuscript; or the decision to submit the manuscript for publication.

Supplemental Figure 1 and Supplemental Tables 1–10 are available from the “Supplemental data” link in the online posting of the article and from the same link in the online table of contents at https://academic.oup.com/jn.

Abbreviations used: BMS, breastmilk substitute; DHS, Demographic and Health Surveys; GDP, gross domestic product; IYCF, infant and young child feeding; LMIC, low- and middle-income country; MICS, Multiple Indicator Cluster Surveys.
