# Supplementary material for: Infant Formula Consumption Is Positively Correlated with Wealth, Within and Between Countries: A Multi-Country Study
Source: J Nutr. 2019 Dec 25;150(4):910–7. doi: 10.1093/jn/nxz327 (PMC7138652; doi:10.1093/jn/nxz327)
Supplement: nxz327_Supplemental_File [file nxz327_supplemental_file.doc]

Title: Infant formula consumption is positively correlated with wealth, within and between countries: a multi-country study

1^st^ author: Paulo A R Neves

“Online Supplementary Material”

National Survey since 1991 analysed to date by ICEH (2019-04-05)

378 surveys from 115 countries

Latest surveys from 2010 onwards

100 surveys

**Excluded surveys (7)**

Small sample size per wealth quintile

*Barbados 2012*

*Bosnia and Herzegovina 2011*

*Macedonia 2011*

*Montenegro 2013*

*St Lucia 2012*

*Trinidad and Tobago 2011*

Lack of data on household wealth

*Cuba 2014*

Number of eligible surveys

97 surveys

**Excluded surveys (3)**

Lack of disaggregated data by wealth quintiles

*Colombia 2015*

*Qatar 2012*

*Uruguay 2011*

Wealth quintiles analyses

**Not included surveys owing to lack of disaggregated data by wealth quintiles for some indicators**

Exclusive breastfeeding (1)

*Philippines 2017*

Infant formula (3)

E*cuador 2012, Mozambique 2015,*

*Philippines 2017*

Other non-human milk (1)

*Philippines 2017*

ICEH - International Center for Equity in Health

**Included surveys in the final analyses by wealth quintiles**

Exclusive breastfeeding under 6 months – 89 surveys

Continued breastfeeding at 1 year – 90 surveys

Infant formula consumption under 6 months – 87 surveys

Other non-human milk consumption under 6 months – 89 surveys

**Supplemental figure 1. Flowchart of national surveys included in the breastfeeding inequalities analyses**

**Supplemental Table 1. Countries and surveys included in the breastfeeding and breastmilk substitutes inequalities analyses. Source: DHS, MICS, and ENSANUT, 2010-2017.1**

| **Country** | **ISO code^2^** | **UNICEF region** | **Income Group** | **Survey and year** | **Number of children aged 0-5 months** | **Number of children aged 12-15 months** |
| --- | --- | --- | --- | --- | --- | --- |
| Afghanistan | AFG | South Asia | Low income | DHS 2015 | 3203 | 2467 |
| Albania | ALB | Eastern Europe & Central Asia | Upper-middle income | DHS 2017 | 285 | 188 |
| Algeria | DZA | Middle East & North Africa | Upper-middle income | MICS 2012 | 1624 | 947 |
| Angola | AGO | Eastern & Southern Africa | Upper-middle income | DHS 2015 | 1620 | 944 |
| Argentina | ARG | Latin America & Caribbean | Upper-middle income | MICS 2011 | 788 | 615 |
| Armenia | ARM | Eastern Europe & Central Asia | Lower-middle income | DHS 2015 | 177 | 118 |
| Bangladesh | BGD | South Asia | Lower-middle income | DHS 2014 | 632 | 552 |
| Belarus | BLR | Eastern Europe & Central Asia | Upper-middle income | MICS 2012 | 247 | 211 |
| Belize | BLZ | Latin America & Caribbean | Upper-middle income | MICS 2015 | 165 | 209 |
| Benin | BEN | West & Central Africa | Low income | MICS 2014 | 1284 | 856 |
| Bhutan | BTN | South Asia | Lower-middle income | MICS 2010 | 608 | 423 |
| Burkina Faso | BFA | West & Central Africa | Low income | DHS 2010 | 1454 | 986 |
| Burundi | BDI | Eastern & Southern Africa | Low income | DHS 2016 | 1236 | 894 |
| Central African Republic | CAF | West & Central Africa | Low income | MICS 2010 | 1283 | 768 |
| Cambodia | KHM | East Asia & the Pacific | Low income | DHS 2014 | 688 | 450 |
| Cameroon | CMR | West & Central Africa | Lower-middle income | MICS 2014 | 703 | 493 |
| Chad | TCD | West & Central Africa | Low income | DHS 2014 | 1823 | 1094 |
| Comoros | COM | Eastern & Southern Africa | Low income | DHS 2012 | 327 | 210 |
| Congo Brazzaville | COG | West & Central Africa | Lower-middle income | MICS 2014 | 899 | 567 |
| Congo Democratic Republic | COD | West & Central Africa | Low income | DHS 2013 | 1934 | 1294 |
| Costa Rica | CRI | Latin America & Caribbean | Upper-middle income | MICS 2011 | 216 | 141 |
| Cote d'Ivoire | CIV | West & Central Africa | Lower-middle income | MICS 2016 | 981 | 657 |
| Dominican Republic | DOM | Latin America & Caribbean | Upper-middle income | MICS 2014 | 1656 | 1082 |
| Ecuador | ECU | Latin America & Caribbean | Upper-middle income | ENSANUT 2012 | 906 | 747 |
| Egypt | EGY | Middle East & North Africa | Lower-middle income | DHS 2014 | 1487 | 1018 |
| El Salvador | SLV | Latin America & Caribbean | Lower-middle income | MICS 2014 | 515 | 490 |
| Eswatini | SWZ | Eastern & Southern Africa | Lower-middle income | MICS 2014 | 235 | 191 |
| Ethiopia | ETH | Eastern & Southern Africa | Low income | DHS 2016 | 1092 | 754 |
| Gabon | GAB | West & Central Africa | Upper-middle income | DHS 2012 | 631 | 381 |
| Gambia | GMB | West & Central Africa | Low income | DHS 2013 | 951 | 640 |
| Ghana | GHA | West & Central Africa | Lower-middle income | DHS 2014 | 606 | 362 |
| Guatemala | GTM | Latin America & Caribbean | Lower-middle income | DHS 2014 | 1175 | 825 |
| Guinea | GIN | West & Central Africa | Low income | MICS 2016 | 686 | 514 |
| Guinea Bissau | GNB | West & Central Africa | Low income | MICS 2014 | 830 | 573 |
| Guyana | GUY | Latin America & Caribbean | Lower-middle income | MICS 2014 | 290 | 204 |
| Haiti | HTI | Latin America & Caribbean | Low income | DHS 2016 | 700 | 425 |
| Honduras | HND | Latin America & Caribbean | Lower-middle income | DHS 2011 | 1084 | 769 |
| India | IND | South Asia | Lower-middle income | DHS 2015 | 22626 | 16237 |
| Indonesia | IDN | East Asia & the Pacific | Lower-middle income | DHS 2012 | 1686 | 1173 |
| Iraq | IRQ | Middle East & North Africa | Lower-middle income | MICS 2011 | 3882 | 2765 |
| Jamaica | JAM | Latin America & Caribbean | Upper-middle income | MICS 2011 | 167 | 118 |
| Jordan | JOR | Middle East & North Africa | Upper-middle income | DHS 2017 | 1218 | 581 |
| Kazakhstan | KAZ | Eastern Europe & Central Asia | Upper-middle income | MICS 2015 | 508 | 381 |
| Kenya | KEN | Eastern & Southern Africa | Lower-middle income | DHS 2014 | 856 | 666 |
| Kosovo | XKX | Eastern Europe & Central Asia | Lower-middle income | MICS 2013 | 145 | 99 |
| Kyrgyzstan | KGZ | Eastern Europe & Central Asia | Lower-middle income | MICS 2014 | 432 | 288 |
| Lao | LAO | East Asia & the Pacific | Lower-middle income | MICS 2011 | 1168 | 802 |
| Lesotho | LSO | Eastern & Southern Africa | Lower-middle income | DHS 2014 | 327 | 260 |
| Liberia | LBR | West & Central Africa | Low income | DHS 2013 | 717 | 472 |
| Malawi | MWI | Eastern & Southern Africa | Low income | DHS 2015 | 1636 | 1118 |
| Maldives | MDV | South Asia | Upper-middle income | DHS 2016 | 288 | 199 |
| Mali | MLI | West & Central Africa | Low income | MICS2015 | 1663 | 1290 |
| Mauritania | MRT | West & Central Africa | Lower-middle income | MICS 2015 | 915 | 884 |
| Mexico | MEX | Latin America & Caribbean | Upper-middle income | MICS 2015 | 666 | 538 |
| Moldova | MDA | Eastern Europe & Central Asia | Lower-middle income | MICS 2012 | 176 | 112 |
| Mongolia | MNG | East Asia & the Pacific | Lower-middle income | MICS 2013 | 644 | 382 |
| Mozambique | MOZ | Eastern & Southern Africa | Low income | DHS 2015 | 514 | 342 |
| Myanmar | MMR | East Asia & the Pacific | Lower-middle income | DHS 2015 | 468 | 333 |
| Namibia | NAM | Eastern & Southern Africa | Upper-middle income | DHS 2013 | 525 | 311 |
| Nepal | NPL | South Asia | Low income | DHS 2016 | 467 | 340 |
| Niger | NER | West & Central Africa | Low income | DHS 2012 | 1303 | 926 |
| Nigeria | NGA | West & Central Africa | Lower-middle income | MICS 2016 | 2748 | 2042 |
| Pakistan | PAK | South Asia | Lower-middle income | DHS 2012 | 1075 | 802 |
| Panama | PAN | Latin America & Caribbean | Upper-middle income | MICS 2013 | 510 | 410 |
| Paraguay | PRY | Latin America & Caribbean | Upper-middle income | MICS 2016 | 394 | 334 |
| Peru | PER | Latin America & Caribbean | Upper-middle income | DHS 2016 | 1446 | 1405 |
| Philippines | PHL | East Asia & the Pacific | Lower-middle income | DHS 2017 | - | 613 |
| Rwanda | RWA | Eastern & Southern Africa | Low income | DHS 2014 | 703 | 502 |
| Sao Tome and Principe | STP | West & Central Africa | Lower-middle income | MICS 2014 | 169 | 137 |
| Senegal | SEN | West & Central Africa | Low income | DHS 2017 | 1142 | 734 |
| Serbia | SRB | Eastern Europe & Central Asia | Upper-middle income | MICS 2014 | 169 | 146 |
| Sierra Leone | SLE | West & Central Africa | Low income | DHS 2013 | 1115 | 803 |
| South Africa | ZAF | Eastern & Southern Africa | Upper-middle income | DHS 2016 | 346 | 203 |
| South Sudan***** | SSD | Eastern & Southern Africa | Low income | MICS 2010 | 877 | 799 |
| State of Palestine | PSE | Middle East & North Africa | Lower-middle income | MICS 2014 | 665 | 506 |
| Sudan | SDN | Middle East & North Africa | Lower-middle income | MICS 2014 | 1543 | 1032 |
| Suriname | SUR | Latin America & Caribbean | Upper-middle income | MICS 2010 | 304 | 187 |
| Tajikistan | TJK | Eastern Europe & Central Asia | Low income | DHS 2017 | 553 | 416 |
| Tanzania | TZA | Eastern & Southern Africa | Low income | DHS 2015 | 1015 | 724 |
| Thailand | THA | East Asia & the Pacific | Upper-middle income | MICS 2015 | 661 | 804 |
| Timor Leste | TLS | East Asia & the Pacific | Lower-middle income | DHS 2016 | 743 | 491 |
| Togo | TGO | West & Central Africa | Low income | DHS 2013 | 603 | 477 |
| Tunisia | TUN | Middle East & North Africa | Upper-middle income | MICS 2011 | 306 | 176 |
| Turkmenistan | TKM | Eastern Europe & Central Asia | Upper-middle income | MICS 2015 | 342 | 270 |
| Uganda | UGA | Eastern & Southern Africa | Low income | DHS 2016 | 1482 | 882 |
| Ukraine | UKR | Eastern Europe & Central Asia | Lower-middle income | MICS 2012 | 307 | 310 |
| Vietnam | VNM | East Asia & the Pacific | Lower-middle income | MICS 2013 | 358 | 265 |
| Yemen | YEM | Middle East & North Africa | Lower-middle income | DHS 2013 | 1672 | 1265 |
| Zambia | ZMB | Eastern & Southern Africa | Lower-middle income | DHS 2013 | 1189 | 863 |
| Zimbabwe | ZWE | Eastern & Southern Africa | Low income | DHS 2015 | 603 | 379 |

^1^DHS: Demographic Health Survey; MICS: Multiple Indicator Cluster Survey; ENSANUT: Encuesta Nacional de Salud y Nutrición; ^2^ISO: International Organization for Standardization; *As South Sudan was not classified on their income by the year of the survey implementation (2010), we used its classification in 2014.

**Supplemental Table 2. Percentage of children under six months of age who were exclusively breastfed at the time of the survey by wealth quintiles, slope and concentration inequalities index. Source: DHS, MICS, and ENSANUT, 2010-2017.1**

| **Country** | **Exclusive breastfeeding under 6 months of age (0-5 months)** | | | | | | | | | | | | | | | | | | | | | | | | | | |
| --- | --- | --- | --- | --- | --- | --- | --- | --- | --- | --- | --- | --- | --- | --- | --- | --- | --- | --- | --- | --- | --- | --- | --- | --- | --- | --- | --- |
|  | **Poorest** | | | | **Second** | | | | **Third** | | | | **Fourth** | | | | **Wealthiest** | | | | **National prevalence** | **SII^2^** | **95% CI** | | **CIX^3^** | **95% CI** | |
|  | **%** | **95% CI** | | **N** | **%** | **95% CI** | | **N** | **%** | **95% CI** | | **N** | **%** | **95% CI** | | **N** | **%** | **95% CI** | | **N** |  |  |  |  |  |  |  |
| Afghanistan | 41.3 | 35.6 | 47.3 | 528 | 40.4 | 34.5 | 46.7 | 754 | 45.8 | 40.2 | 51.5 | 759 | 48.1 | 41.0 | 55.4 | 710 | 40.5 | 33.5 | 48.0 | 452 | 43.3 | 2.8 | -8.2 | 13.7 | 1.7 | -2.6 | 6.0 |
| Albania | 35.1 | 24.1 | 47.9 | 84 | 33.5 | 21.2 | 48.6 | 60 | 41.4 | 26.9 | 57.6 | 55 | 38.5 | 24.4 | 54.8 | 59 | 33.2 | 15.8 | 56.8 | 27 | 36.7 | 0.9 | -24.4 | 26.3 | -1.3 | -12.9 | 10.4 |
| Algeria | 25.7 | 20.4 | 31.8 | 399 | 23.1 | 17.7 | 29.6 | 344 | 29.4 | 22.5 | 37.4 | 316 | 21.4 | 16.4 | 27.5 | 312 | 30.3 | 22.9 | 39.0 | 253 | 25.7 | 3.0 | -7.3 | 13.4 | 1.6 | -5.1 | 8.4 |
| Angola | 37.4 | 31.7 | 43.5 | 413 | 33.7 | 28.6 | 39.2 | 448 | 44.2 | 37.9 | 50.7 | 418 | 35.4 | 26.0 | 46.0 | 211 | 39.0 | 29.1 | 49.9 | 130 | 38.0 | 2.8 | -10.3 | 15.9 | 0.5 | -5.4 | 6.3 |
| Argentina | 30.4 | 21.6 | 40.8 | 214 | 33.7 | 23.9 | 45.1 | 143 | 31.6 | 22.0 | 43.1 | 159 | 16.1 | 9.6 | 25.7 | 149 | 55.9 | 39.6 | 71.0 | 123 | 33.5 | 15.6 | -6.4 | 37.5 | 6.9 | -3.3 | 17.1 |
| Armenia | 44.5 | 30.0 | 60.1 | 28 | 57.9 | 39.5 | 74.4 | 37 | 51.1 | 33.4 | 68.5 | 39 | 35.4 | 19.2 | 55.7 | 35 | 37.5 | 22.2 | 55.8 | 38 | 44.5 | -19.4 | -46.0 | 7.1 | -6.6 | -17.6 | 4.3 |
| Bangladesh | 46.1 | 34.1 | 58.6 | 122 | 68.8 | 57.2 | 78.5 | 130 | 52.5 | 38.4 | 66.2 | 121 | 57.8 | 46.9 | 68.0 | 120 | 53.4 | 42.3 | 64.1 | 139 | 55.3 | 2.4 | -16.5 | 21.2 | 1.2 | -4.8 | 7.2 |
| Belarus | 28.6 | 11.5 | 55.2 | 36 | 16.3 | 6.0 | 37.1 | 51 | 11.5 | 3.4 | 32.1 | 41 | 8.1 | 3.4 | 17.8 | 52 | 27.9 | 15.8 | 44.5 | 67 | 19.0 | 3.4 | -26.2 | 32.9 | 1.0 | -25.0 | 27.0 |
| Belize | 45.3 | 29.5 | 62.0 | 40 | 27.6 | 15.0 | 45.2 | 37 | 21.3 | 9.6 | 41.0 | 34 | 37.1 | 16.4 | 64.0 | 33 | 37.7 | 17.2 | 63.8 | 21 | 33.2 | -4.0 | -37.3 | 29.3 | -1.3 | -17.0 | 14.4 |
| Benin | 39.5 | 32.4 | 47.1 | 221 | 41.4 | 34.7 | 48.3 | 241 | 39.3 | 30.9 | 48.4 | 233 | 46.1 | 39.3 | 53.0 | 294 | 39.4 | 32.5 | 46.6 | 295 | 41.4 | 3.0 | -7.4 | 13.4 | 0.9 | -3.4 | 5.1 |
| Bhutan | 36.1 | 25.6 | 48.1 | 123 | 50.3 | 39.1 | 61.5 | 115 | 42.8 | 33.2 | 53.0 | 149 | 47.1 | 36.4 | 58.1 | 119 | 65.0 | 51.7 | 76.4 | 102 | 48.7 | 29.8 | 11.4 | 48.2 | 10.2 | 4.0 | 16.4 |
| Burkina Faso | 23.9 | 18.6 | 30.0 | 275 | 21.4 | 16.6 | 27.1 | 324 | 22.9 | 18.0 | 28.7 | 329 | 28.1 | 22.8 | 34.0 | 318 | 29.7 | 22.8 | 37.6 | 208 | 24.8 | 8.3 | -0.9 | 17.4 | 5.2 | -1.1 | 11.5 |
| Burundi | 82.1 | 76.1 | 86.8 | 233 | 80.5 | 74.8 | 85.2 | 241 | 86.7 | 81.2 | 90.7 | 222 | 88.6 | 83.5 | 92.2 | 236 | 77.7 | 71.2 | 83.0 | 304 | 83.1 | 0.3 | -8.3 | 9.0 | -0.2 | -1.9 | 1.5 |
| Cambodia | 75.5 | 64.3 | 84.1 | 156 | 74.3 | 64.4 | 82.3 | 121 | 66.3 | 57.1 | 74.4 | 119 | 65.1 | 53.8 | 75.0 | 134 | 40.5 | 30.3 | 51.6 | 158 | 65.2 | -35.9 | -51.6 | -20.2 | -9.5 | -13.7 | -5.2 |
| Cameroon | 9.5 | 5.2 | 16.8 | 117 | 27.5 | 19.9 | 36.7 | 153 | 37.4 | 29.1 | 46.5 | 174 | 38.6 | 29.7 | 48.3 | 147 | 31.5 | 22.1 | 42.7 | 112 | 28.2 | 30.9 | 18.5 | 43.3 | 17.9 | 10.3 | 25.5 |
| CAR^4^ | 27.4 | 21.5 | 34.3 | 277 | 34.5 | 28.1 | 41.5 | 314 | 36.0 | 30.0 | 42.6 | 325 | 41.4 | 33.6 | 49.6 | 233 | 29.3 | 19.4 | 41.5 | 134 | 33.7 | 7.5 | -5.7 | 20.7 | 3.5 | -2.8 | 9.9 |
| Chad | 0.6 | 0.2 | 2.3 | 355 | 0.0 | 0.0 | 0.0 | 356 | 0.2 | 0.0 | 1.0 | 375 | 0.0 | 0.0 | 0.0 | 405 | 0.7 | 0.2 | 2.4 | 332 | 0.3 | 0.0 | -1.1 | 1.2 | 3.6 | -63.6 | 70.9 |
| Comoros | 7.1 | 2.8 | 16.9 | 89 | 8.5 | 3.5 | 19.2 | 66 | 15.5 | 7.8 | 28.5 | 61 | 20.4 | 10.6 | 35.6 | 54 | 11.7 | 4.2 | 28.5 | 57 | 12.1 | 11.3 | -4.0 | 26.7 | 18.8 | -1.8 | 39.3 |
| Congo Brazzaville | 21.2 | 16.8 | 26.3 | 432 | 37.4 | 27.1 | 49.0 | 219 | 24.7 | 16.8 | 34.7 | 112 | 42.1 | 29.5 | 55.9 | 78 | 43.7 | 28.1 | 60.6 | 58 | 32.9 | 24.8 | 9.3 | 40.2 | 14.1 | 7.1 | 21.0 |
| CDR^5^ | 47.3 | 40.8 | 54.0 | 535 | 46.7 | 39.6 | 54.0 | 434 | 54.4 | 46.1 | 62.4 | 369 | 51.1 | 44.3 | 57.9 | 333 | 37.7 | 29.5 | 46.7 | 263 | 47.6 | -6.4 | -18.3 | 5.5 | -2.7 | -6.7 | 1.4 |
| Costa Rica | 33.4 | 21.2 | 48.4 | 90 | 32.4 | 13.7 | 59.2 | 48 | 40.2 | 19.0 | 65.8 | 32 | 12.4 | 2.1 | 48.1 | 24 | 36.4 | 10.0 | 74.5 | 22 | 32.5 | -3.5 | -45.0 | 38.1 | -3.9 | -25.6 | 17.8 |
| Cote d’Ivoire | 26.0 | 20.2 | 32.8 | 316 | 21.6 | 16.0 | 28.7 | 253 | 21.3 | 15.5 | 28.5 | 219 | 24.6 | 16.7 | 34.8 | 119 | 23.4 | 13.5 | 37.5 | 74 | 23.5 | -2.5 | -15.0 | 10.0 | -2.1 | -10.9 | 6.7 |
| Dominican Republic | 7.2 | 3.9 | 12.8 | 552 | 5.3 | 2.8 | 10.0 | 377 | 3.4 | 1.7 | 6.7 | 293 | 2.4 | 1.0 | 5.4 | 253 | 3.7 | 1.8 | 7.2 | 181 | 4.7 | -5.6 | -11.3 | 0.2 | -17.9 | -34.1 | -1.7 |
| Ecuador | 47.3 | 38.4 | 56.4 | 312 | 42.6 | 32.7 | 53.2 | 222 | 38.9 | 30.7 | 47.8 | 168 | 42.5 | 30.8 | 55.2 | 115 | 34.8 | 22.4 | 49.6 | 89 | 42.0 | -14.6 | -30.0 | 0.8 | -5.8 | -12.4 | 0.8 |
| Egypt | 39.4 | 33.2 | 45.9 | 299 | 44.6 | 38.2 | 51.2 | 263 | 37.1 | 31.5 | 43.0 | 324 | 42.0 | 35.4 | 49.0 | 314 | 35.1 | 28.1 | 42.8 | 287 | 39.7 | -4.2 | -14.0 | 5.7 | -2.2 | -6.3 | 2.0 |
| El Salvador | 55.7 | 45.3 | 65.6 | 144 | 43.8 | 32.2 | 56.1 | 104 | 43.1 | 29.9 | 57.4 | 98 | 40.7 | 26.7 | 56.4 | 86 | 45.8 | 29.8 | 62.8 | 83 | 46.7 | -15.3 | -36.7 | 6.1 | -6.3 | -14.1 | 1.5 |
| Eswatini | 67.0 | 54.5 | 77.5 | 65 | 71.9 | 57.8 | 82.8 | 59 | 56.0 | 40.9 | 70.0 | 56 | 67.0 | 39.9 | 86.1 | 30 | 50.2 | 26.7 | 73.6 | 25 | 63.8 | -15.2 | -44.2 | 13.7 | -3.7 | -11.5 | 4.2 |
| Ethiopia | 55.1 | 46.6 | 63.2 | 418 | 63.4 | 54.5 | 71.6 | 174 | 55.0 | 43.2 | 66.3 | 129 | 47.3 | 37.1 | 57.7 | 145 | 67.1 | 55.7 | 76.8 | 226 | 57.5 | 1.9 | -12.4 | 16.1 | 0.7 | -3.5 | 4.9 |
| Gabon | 6.3 | 3.6 | 10.9 | 300 | 5.3 | 1.9 | 13.7 | 137 | 3.5 | 0.7 | 15.9 | 88 | 15.6 | 6.0 | 35.1 | 60 | 0.7 | 0.1 | 4.8 | 46 | 6.0 | -0.3 | -9.2 | 8.7 | -4.4 | -29.4 | 20.7 |
| Gambia | 53.6 | 45.7 | 61.3 | 230 | 54.5 | 45.6 | 63.2 | 235 | 43.4 | 35.0 | 52.1 | 192 | 38.5 | 30.5 | 47.2 | 177 | 43.5 | 27.5 | 60.9 | 117 | 46.8 | -18.6 | -36.5 | -0.8 | -7.3 | -14.0 | -0.5 |
| Ghana | 62.0 | 52.5 | 70.8 | 210 | 45.3 | 35.4 | 55.6 | 121 | 55.2 | 43.3 | 66.5 | 112 | 52.3 | 39.4 | 64.9 | 91 | 42.6 | 31.1 | 55.1 | 72 | 52.3 | -16.1 | -33.0 | 0.9 | -4.7 | -10.5 | 1.1 |
| Guatemala | 75.3 | 69.2 | 80.6 | 318 | 61.7 | 55.0 | 68.0 | 287 | 50.2 | 41.6 | 58.8 | 222 | 31.7 | 24.5 | 39.8 | 206 | 24.9 | 18.0 | 33.5 | 142 | 53.2 | -58.8 | -67.6 | -49.9 | -20.1 | -23.7 | -16.5 |
| **Country** | **Exclusive breastfeeding under 6 months of age (0-5 months)** | | | | | | | | | | | | | | | | | | | | | | | | | | |
|  | **Poorest** | | | | **Second** | | | | **Third** | | | | **Fourth** | | | | **Wealthiest** | | | | **National prevalence** | **SII** | **95% CI** | | **CIX** | **95% CI** | |
|  | **%** | **95% CI** | | **N** | **%** | **95% CI** | | **N** | **%** | **95% CI** | | **N** | **%** | **95% CI** | | **N** | **%** | **95% CI** | | **N** |  |  |  |  |  |  |  |
| Guinea | 24.7 | 18.2 | 32.5 | 168 | 41.6 | 32.3 | 51.6 | 162 | 26.9 | 19.2 | 36.4 | 139 | 42.5 | 32.4 | 53.4 | 133 | 41.3 | 30.8 | 52.8 | 84 | 35.2 | 15.6 | 0.8 | 30.5 | 7.4 | 0.2 | 14.5 |
| Guinea Bissau | 56.0 | 49.3 | 62.4 | 260 | 45.4 | 38.1 | 53.0 | 209 | 60.3 | 52.7 | 67.4 | 198 | 50.5 | 38.5 | 62.5 | 94 | 48.1 | 34.3 | 62.2 | 69 | 52.5 | -3.3 | -18.9 | 12.2 | -1.1 | -5.9 | 3.6 |
| Guyana | 30.9 | 21.0 | 42.9 | 114 | 17.2 | 9.2 | 30.1 | 59 | 26.2 | 13.7 | 44.1 | 50 | 13.6 | 4.8 | 32.9 | 33 | 21.3 | 9.5 | 41.3 | 34 | 23.3 | -13.5 | -34.7 | 7.7 | -11.3 | -26.9 | 4.2 |
| Haiti | 33.9 | 25.5 | 43.4 | 200 | 41.3 | 32.6 | 50.5 | 189 | 47.4 | 35.7 | 59.4 | 141 | 37.1 | 25.7 | 50.1 | 105 | 37.1 | 24.8 | 51.3 | 65 | 39.9 | 4.8 | -11.6 | 21.1 | 2.5 | -4.0 | 9.1 |
| Honduras | 51.5 | 45.4 | 57.5 | 341 | 36.2 | 29.9 | 43.1 | 270 | 23.4 | 17.7 | 30.3 | 185 | 16.1 | 10.9 | 23.1 | 178 | 22.8 | 15.1 | 32.9 | 110 | 31.2 | -40.9 | -51.1 | -30.8 | -21.9 | -28.1 | -15.7 |
| India | 55.9 | 54.3 | 57.5 | 5829 | 56.6 | 54.8 | 58.4 | 5494 | 54.3 | 52.2 | 56.3 | 4508 | 53.1 | 50.6 | 55.6 | 3660 | 53.8 | 51.1 | 56.5 | 3135 | 54.9 | -3.8 | -7.0 | -0.5 | -1.2 | -2.2 | -0.2 |
| Indonesia | 43.6 | 37.9 | 49.5 | 510 | 41.2 | 34.4 | 48.3 | 350 | 43.7 | 35.4 | 52.4 | 281 | 33.1 | 25.7 | 41.4 | 310 | 46.4 | 37.4 | 55.7 | 235 | 41.5 | -2.3 | -14.8 | 10.2 | -1.0 | -5.9 | 3.9 |
| Iraq | 22.6 | 19.4 | 26.1 | 1320 | 23.7 | 19.4 | 28.7 | 939 | 16.6 | 12.7 | 21.5 | 691 | 16.8 | 12.6 | 22.0 | 533 | 16.6 | 11.8 | 22.8 | 399 | 19.6 | -9.5 | -16.4 | -2.6 | -8.4 | -14.4 | -2.4 |
| Jamaica | 20.6 | 8.8 | 41.1 | 28 | 32.8 | 17.0 | 53.8 | 39 | 15.8 | 8.2 | 28.3 | 47 | 25.9 | 12.5 | 46.0 | 30 | 24.9 | 9.4 | 51.4 | 23 | 23.8 | -2.9 | -29.4 | 23.7 | -5.2 | -24.0 | 13.6 |
| Jordan | 26.5 | 20.9 | 32.9 | 441 | 26.2 | 19.3 | 34.5 | 312 | 22.9 | 15.6 | 32.3 | 241 | 27.4 | 19.1 | 37.7 | 164 | 23.3 | 11.9 | 40.8 | 60 | 25.5 | -1.9 | -14.9 | 11.2 | -1.9 | -10.3 | 6.5 |
| Kazakhstan | 35.2 | 26.0 | 45.5 | 82 | 35.8 | 24.3 | 49.2 | 90 | 45.3 | 31.4 | 60.0 | 142 | 35.8 | 26.6 | 46.2 | 102 | 33.2 | 23.2 | 45.0 | 92 | 37.8 | -1.2 | -18.2 | 15.8 | -1.2 | -8.4 | 5.9 |
| Kenya | 55.8 | 49.4 | 62.1 | 320 | 57.9 | 48.1 | 67.1 | 167 | 53.6 | 44.1 | 62.9 | 140 | 74.8 | 61.1 | 84.8 | 119 | 69.8 | 56.6 | 80.3 | 110 | 61.4 | 20.2 | 5.9 | 34.5 | 5.2 | 1.2 | 9.2 |
| Kosovo | 34.0 | 20.7 | 50.4 | 38 | 36.0 | 17.4 | 60.0 | 24 | 43.4 | 25.9 | 62.6 | 28 | 37.2 | 20.2 | 58.1 | 25 | 48.8 | 33.3 | 64.5 | 30 | 39.9 | 16.3 | -9.7 | 42.3 | 5.3 | -5.8 | 16.4 |
| Kyrgyzstan | 39.5 | 28.2 | 52.0 | 89 | 39.5 | 28.8 | 51.4 | 97 | 46.4 | 34.8 | 58.5 | 93 | 44.7 | 32.8 | 57.3 | 79 | 36.0 | 24.6 | 49.2 | 74 | 41.1 | -1.4 | -21.5 | 18.7 | -0.8 | -9.1 | 7.4 |
| Lao | 46.6 | 40.0 | 53.4 | 325 | 39.7 | 32.9 | 46.9 | 273 | 41.8 | 35.3 | 48.7 | 253 | 36.8 | 28.7 | 45.8 | 175 | 34.1 | 26.3 | 42.9 | 142 | 40.4 | -13.3 | -25.3 | -1.3 | -6.3 | -11.3 | -1.3 |
| Lesotho | 68.8 | 58.3 | 77.6 | 82 | 73.4 | 61.5 | 82.6 | 76 | 74.8 | 62.2 | 84.3 | 67 | 69.2 | 54.3 | 80.9 | 59 | 40.5 | 22.4 | 61.6 | 43 | 66.9 | -23.2 | -46.8 | 0.3 | -5.3 | -11.5 | 0.9 |
| Liberia | 60.5 | 52.4 | 68.0 | 256 | 62.7 | 53.8 | 70.8 | 194 | 59.5 | 47.9 | 70.2 | 148 | 47.1 | 32.6 | 62.1 | 77 | 36.9 | 21.2 | 55.8 | 42 | 55.2 | -25.6 | -43.8 | -7.5 | -8.8 | -14.6 | -3.0 |
| Malawi | 58.9 | 52.7 | 64.8 | 379 | 55.7 | 49.4 | 61.8 | 358 | 61.5 | 55.0 | 67.6 | 317 | 60.6 | 53.4 | 67.4 | 293 | 71.4 | 64.4 | 77.5 | 289 | 61.0 | 12.7 | 2.1 | 23.3 | 3.5 | 0.7 | 6.4 |
| Maldives | 59.2 | 47.2 | 70.2 | 86 | 60.4 | 49.6 | 70.3 | 82 | 67.6 | 54.9 | 78.2 | 73 | 59.9 | 37.9 | 78.6 | 28 | 68.6 | 42.4 | 86.6 | 19 | 63.5 | 9.9 | -19.6 | 39.3 | 3.9 | -3.2 | 11.0 |
| Mali | 31.4 | 25.6 | 37.9 | 345 | 32.8 | 27.1 | 39.0 | 362 | 32.0 | 26.9 | 37.5 | 357 | 33.7 | 27.4 | 40.6 | 303 | 33.2 | 27.1 | 39.9 | 296 | 32.6 | 2.1 | -7.2 | 11.4 | 0.9 | -3.9 | 5.7 |
| Mauritania | 57.5 | 49.5 | 65.1 | 198 | 46.7 | 39.2 | 54.4 | 220 | 42.1 | 33.9 | 50.7 | 190 | 24.1 | 16.6 | 33.6 | 147 | 25.6 | 17.5 | 35.9 | 160 | 41.4 | -42.9 | -55.3 | -30.5 | -17.1 | -22.5 | -11.7 |
| Mexico | 33.7 | 25.0 | 43.8 | 189 | 32.0 | 21.6 | 44.7 | 203 | 33.9 | 14.4 | 61.1 | 134 | 24.1 | 12.4 | 41.5 | 93 | 19.5 | 7.3 | 42.6 | 47 | 30.8 | -11.1 | -32.7 | 10.5 | -8.4 | -18.9 | 2.1 |
| Moldova | 48.8 | 27.6 | 70.4 | 21 | 32.8 | 19.2 | 49.9 | 38 | 39.4 | 20.6 | 62.1 | 27 | 28.6 | 15.0 | 47.7 | 34 | 34.9 | 23.2 | 48.6 | 56 | 36.4 | -11.7 | -36.7 | 13.3 | -4.1 | -15.6 | 7.3 |
| Mongolia | 55.4 | 46.5 | 63.9 | 146 | 51.4 | 43.3 | 59.5 | 150 | 43.6 | 34.2 | 53.4 | 129 | 39.3 | 29.8 | 49.6 | 108 | 44.3 | 34.6 | 54.4 | 111 | 47.1 | -17.1 | -31.7 | -2.4 | -5.8 | -11.2 | -0.5 |
| Mozambique | 55.4 | 43.9 | 66.4 | 91 | 53.4 | 39.5 | 66.8 | 95 | 53.0 | 38.7 | 66.8 | 86 | 55.1 | 42.1 | 67.3 | 123 | 56.5 | 44.9 | 67.5 | 119 | 54.6 | 1.4 | -17.1 | 19.9 | -0.3 | -6.0 | 5.4 |
| Myanmar | 52.2 | 40.7 | 63.5 | 119 | 38.6 | 26.9 | 51.8 | 101 | 52.8 | 39.1 | 66.1 | 87 | 51.8 | 39.5 | 64.0 | 87 | 61.8 | 49.3 | 72.9 | 74 | 51.2 | 12.9 | -6.9 | 32.7 | 4.8 | -1.9 | 11.5 |
| Namibia | 57.1 | 47.8 | 66.0 | 122 | 50.9 | 41.2 | 60.6 | 124 | 43.8 | 34.5 | 53.5 | 116 | 41.1 | 30.1 | 53.1 | 97 | 46.7 | 33.3 | 60.6 | 66 | 48.5 | -17.6 | -35.4 | 0.3 | -4.6 | -11.0 | 1.7 |
| Nepal | 71.3 | 61.8 | 79.2 | 131 | 62.2 | 50.3 | 72.8 | 88 | 73.3 | 62.2 | 82.1 | 98 | 60.1 | 47.5 | 71.5 | 82 | 59.7 | 46.1 | 72.0 | 68 | 66.1 | -12.4 | -29.8 | 4.9 | -3.9 | -8.0 | 0.3 |
| Niger | 25.2 | 18.7 | 32.9 | 268 | 20.0 | 14.9 | 26.5 | 241 | 22.1 | 13.8 | 33.4 | 243 | 20.7 | 15.8 | 26.7 | 264 | 29.5 | 22.7 | 37.3 | 287 | 23.3 | 2.5 | -8.4 | 13.5 | 3.1 | -4.8 | 11.1 |
| Nigeria | 16.4 | 12.9 | 20.7 | 611 | 20.0 | 16.4 | 24.3 | 638 | 23.7 | 17.4 | 31.3 | 525 | 27.1 | 22.5 | 32.1 | 551 | 35.8 | 30.1 | 42.0 | 423 | 23.7 | 20.9 | 13.3 | 28.4 | 16.4 | 11.3 | 21.5 |
| Pakistan | 34.7 | 27.8 | 42.3 | 233 | 40.1 | 31.8 | 49.1 | 199 | 44.0 | 35.6 | 52.9 | 212 | 32.8 | 25.3 | 41.1 | 217 | 37.7 | 29.2 | 46.9 | 214 | 37.8 | -0.4 | -14.0 | 13.1 | -0.4 | -6.3 | 5.6 |
| Panama | 38.1 | 29.7 | 47.2 | 285 | 13.3 | 4.9 | 31.6 | 86 | 11.3 | 3.2 | 33.1 | 67 | 21.9 | 7.5 | 49.1 | 42 | 19.1 | 6.6 | 44.1 | 30 | 21.5 | -18.0 | -41.9 | 6.0 | -22.1 | -39.8 | -4.4 |
| Paraguay | 36.3 | 23.3 | 51.7 | 105 | 29.8 | 18.6 | 44.0 | 92 | 29.9 | 17.1 | 46.9 | 80 | 27.3 | 14.6 | 45.3 | 68 | 31.2 | 16.4 | 51.3 | 49 | 31.3 | -8.6 | -33.5 | 16.2 | -7.2 | -20.0 | 5.6 |
| Peru | 86.0 | 81.8 | 89.3 | 399 | 73.4 | 67.6 | 78.5 | 401 | 64.4 | 56.3 | 71.7 | 280 | 57.7 | 49.3 | 65.7 | 213 | 55.8 | 45.6 | 65.6 | 153 | 69.8 | -37.9 | -47.9 | -28.0 | -9.7 | -12.3 | -7.0 |
| Rwanda | 88.4 | 81.4 | 93.0 | 152 | 87.2 | 80.2 | 91.9 | 141 | 86.7 | 79.6 | 91.6 | 124 | 88.9 | 82.0 | 93.3 | 131 | 85.2 | 79.0 | 89.8 | 155 | 87.3 | -2.4 | -11.3 | 6.5 | -0.4 | -2.2 | 1.3 |
| **Country** | **Exclusive breastfeeding under 6 months of age (0-5 months)** | | | | | | | | | | | | | | | | | | | | | | | | | | |
|  | **Poorest** | | | | **Second** | | | | **Third** | | | | **Fourth** | | | | **Wealthiest** | | | | **National prevalence** | **SII** | **95% CI** | | **CIX** | **95% CI** | |
|  | **%** | **95% CI** | | **N** | **%** | **95% CI** | | **N** | **%** | **95% CI** | | **N** | **%** | **95% CI** | | **N** | **%** | **95% CI** | | **N** |  |  |  |  |  |  |  |
| Sao Tome and Principe | 73.1 | 55.1 | 85.8 | 44 | 66.3 | 45.5 | 82.2 | 35 | 73.7 | 57.5 | 85.3 | 40 | 80.0 | 60.2 | 91.3 | 35 | 77.8 | 47.8 | 93.0 | 15 | 73.8 | 11.6 | -16.2 | 39.5 | 2.9 | -3.3 | 9.0 |
| Senegal | 37.5 | 31.4 | 44.1 | 350 | 42.1 | 36.1 | 48.5 | 306 | 41.7 | 34.3 | 49.4 | 244 | 43.8 | 35.0 | 53.0 | 151 | 48.9 | 37.7 | 60.2 | 91 | 42.1 | 11.0 | -1.0 | 23.0 | 5.0 | 0.3 | 9.7 |
| Serbia | 0.0 | 0.0 | 0.0 | 17 | 1.2 | 0.1 | 9.3 | 23 | 8.5 | 1.7 | 33.1 | 40 | 6.3 | 1.1 | 28.1 | 39 | 31.1 | 11.2 | 61.6 | 50 | 12.8 | 35.8 | -0.1 | 71.7 | 38.7 | 11.3 | 66.1 |
| Sierra Leone | 30.9 | 24.3 | 38.4 | 246 | 33.9 | 26.1 | 42.6 | 215 | 41.9 | 34.3 | 50.0 | 266 | 27.2 | 20.8 | 34.6 | 229 | 20.9 | 13.4 | 31.2 | 159 | 32.0 | -9.4 | -21.5 | 2.7 | -3.9 | -10.3 | 2.6 |
| South Africa | 29.8 | 17.4 | 46.1 | 68 | 47.9 | 34.7 | 61.3 | 86 | 31.7 | 21.4 | 44.1 | 89 | 23.2 | 13.1 | 37.7 | 60 | 23.3 | 12.0 | 40.2 | 43 | 31.6 | -18.4 | -40.1 | 3.3 | -8.5 | -19.8 | 2.8 |
| South Sudan | 50.9 | 41.8 | 60.0 | 156 | 45.9 | 38.3 | 53.8 | 164 | 44.3 | 35.9 | 53.0 | 166 | 43.4 | 36.5 | 50.5 | 195 | 41.5 | 33.8 | 49.7 | 196 | 45.1 | -10.6 | -24.1 | 2.9 | -3.9 | -8.9 | 1.1 |
| State of Palestine | 37.2 | 30.0 | 45.1 | 154 | 37.2 | 27.1 | 48.6 | 128 | 41.9 | 32.6 | 51.9 | 121 | 36.8 | 28.7 | 45.8 | 154 | 41.8 | 32.3 | 51.9 | 108 | 38.6 | 3.8 | -10.0 | 17.7 | 1.0 | -5.0 | 7.0 |
| Sudan | 58.4 | 51.3 | 65.2 | 367 | 55.9 | 49.0 | 62.5 | 435 | 52.4 | 44.6 | 60.2 | 352 | 53.4 | 42.0 | 64.5 | 223 | 57.0 | 48.3 | 65.2 | 166 | 55.4 | -4.5 | -18.4 | 9.5 | -1.2 | -5.4 | 3.0 |
| Suriname | 2.4 | 0.9 | 6.5 | 173 | 3.0 | 0.7 | 11.6 | 38 | 1.3 | 0.2 | 8.9 | 39 | 6.8 | 1.8 | 22.4 | 36 | 0.0 | 0.0 | 0.0 | 18 | 2.8 | 1.2 | -6.1 | 8.5 | -3.8 | -46.0 | 38.5 |
| Tajikistan | 43.3 | 32.9 | 54.5 | 99 | 43.3 | 31.3 | 56.1 | 79 | 29.2 | 20.2 | 40.1 | 112 | 37.4 | 28.7 | 47.2 | 121 | 27.6 | 19.5 | 37.6 | 142 | 35.8 | -16.3 | -33.5 | 0.9 | -7.5 | -15.4 | 0.3 |
| Tanzania | 58.9 | 51.6 | 65.8 | 258 | 63.0 | 54.5 | 70.8 | 209 | 58.7 | 50.5 | 66.4 | 179 | 58.1 | 49.3 | 66.3 | 214 | 56.8 | 47.2 | 65.9 | 155 | 59.2 | -3.5 | -16.9 | 10.0 | -0.7 | -4.7 | 3.2 |
| Thailand | 24.0 | 13.3 | 39.2 | 151 | 18.2 | 9.0 | 33.4 | 153 | 24.7 | 13.2 | 41.4 | 136 | 18.5 | 8.1 | 36.7 | 130 | 36.4 | 17.3 | 61.0 | 91 | 23.1 | 8.5 | -15.7 | 32.6 | 3.5 | -14.1 | 21.1 |
| Timor Leste | 58.6 | 48.7 | 67.9 | 126 | 50.5 | 41.0 | 60.0 | 150 | 56.3 | 47.4 | 64.8 | 158 | 50.6 | 41.9 | 59.3 | 160 | 38.9 | 26.7 | 52.6 | 149 | 50.7 | -19.4 | -36.9 | -1.9 | -6.9 | -13.1 | -0.8 |
| Togo | 55.5 | 47.4 | 63.3 | 199 | 50.2 | 39.3 | 61.1 | 114 | 56.2 | 46.4 | 65.6 | 102 | 64.6 | 54.3 | 73.6 | 97 | 60.9 | 49.6 | 71.2 | 91 | 57.5 | 11.6 | -4.3 | 27.5 | 2.4 | -2.3 | 7.0 |
| Tunisia | 15.7 | 8.3 | 27.6 | 78 | 9.2 | 3.9 | 20.4 | 65 | 1.4 | 0.3 | 5.7 | 50 | 9.1 | 4.0 | 19.2 | 75 | 7.1 | 2.0 | 22.7 | 38 | 8.5 | -7.2 | -21.9 | 7.5 | -16.8 | -43.8 | 10.2 |
| Turkmenistan | 58.4 | 43.7 | 71.8 | 49 | 57.5 | 46.6 | 67.7 | 59 | 62.5 | 50.0 | 73.6 | 70 | 64.8 | 52.3 | 75.5 | 84 | 50.5 | 37.9 | 63.0 | 80 | 58.9 | -3.8 | -24.7 | 17.1 | -2.0 | -7.7 | 3.8 |
| Uganda | 70.8 | 64.9 | 76.1 | 400 | 69.3 | 63.5 | 74.5 | 331 | 67.0 | 60.1 | 73.2 | 286 | 57.0 | 49.7 | 64.0 | 255 | 61.3 | 53.3 | 68.7 | 210 | 65.5 | -15.4 | -25.4 | -5.5 | -4.3 | -6.9 | -1.8 |
| Ukraine | 20.4 | 8.9 | 40.4 | 65 | 29.0 | 15.9 | 46.8 | 76 | 10.7 | 3.6 | 27.6 | 49 | 18.3 | 9.6 | 32.3 | 57 | 17.7 | 7.1 | 37.4 | 60 | 19.7 | -9.7 | -34.5 | 15.1 | -5.7 | -28.6 | 17.3 |
| Vietnam | 41.6 | 30.9 | 53.2 | 89 | 20.6 | 11.9 | 33.3 | 68 | 24.1 | 13.6 | 38.9 | 74 | 12.0 | 4.6 | 27.9 | 68 | 22.2 | 12.5 | 36.2 | 59 | 24.3 | -24.1 | -41.9 | -6.4 | -17.3 | -29.6 | -5.0 |
| Yemen | 10.4 | 6.7 | 15.6 | 366 | 11.7 | 8.1 | 16.7 | 376 | 10.6 | 7.1 | 15.5 | 360 | 10.3 | 6.0 | 17.1 | 343 | 7.4 | 3.9 | 13.7 | 227 | 10.3 | -3.0 | -10.2 | 4.2 | -5.2 | -17.0 | 6.5 |
| Zambia | 69.1 | 62.7 | 74.8 | 288 | 72.6 | 67.0 | 77.7 | 309 | 78.4 | 72.1 | 83.7 | 251 | 73.4 | 65.9 | 79.8 | 203 | 63.6 | 52.5 | 73.4 | 138 | 71.9 | -0.6 | -12.0 | 10.8 | -0.5 | -3.1 | 2.2 |
| Zimbabwe | 50.1 | 41.9 | 58.4 | 120 | 55.2 | 45.2 | 64.7 | 117 | 44.8 | 35.0 | 55.0 | 99 | 45.4 | 36.2 | 54.9 | 161 | 40.5 | 30.5 | 51.4 | 106 | 47.7 | -13.1 | -28.0 | 1.8 | -4.2 | -9.4 | 1.0 |

^1^DHS: Demographic Health Survey; MICS: Multiple Indicator Cluster Survey; ENSANUT: Encuesta Nacional de Salud y Nutrición; ^2^SII: slope index of inequality; ^3^CIX: concentration index of inequality; ^4^CAR: Central African Republic; ^5^CDR: Congo Democratic Republic.

**Supplemental Table 3. Average weighted prevalence of exclusive breastfeeding under 6 months of age by wealth quintiles according to regions and national income groups. Source: DHS, MICS, and ENSANUT, 2010-2017.1**

|  | **Wealth quintiles** | | | | |
| --- | --- | --- | --- | --- | --- |
|  | **Poorest** | **Second** | **Third** | **Fourth** | **Wealthiest** |
| **UNICEF regions** |  |  |  |  |  |
| East Asia & Pacific | 39.2 | 32.7 | 36.0 | 28.1 | 37.5 |
| South Asia | 51.6 | 54.8 | 52.6 | 50.5 | 51.0 |
| Eastern Europe & Central Asia | 32.7 | 34.4 | 30.9 | 30.9 | 29.7 |
| Middle East & North Africa | 34.3 | 35.9 | 31.8 | 33.0 | 31.6 |
| West & Central Africa | 27.9 | 29.5 | 32.8 | 34.3 | 34.7 |
| Eastern & Southern Africa | 56.5 | 59.9 | 56.5 | 54.4 | 58.0 |
| Latin America & Caribbean | 42.9 | 39.0 | 37.1 | 28.1 | 30.9 |
|  |  |  |  |  |  |
| **World Bank income groups** |  |  |  |  |  |
| Upper-middle income | 34.9 | 34.4 | 34.6 | 27.1 | 31.5 |
| Lower-middle income | 43.9 | 45.4 | 44.3 | 42.9 | 45.0 |
| Low income | 50.8 | 52.2 | 51.6 | 49.4 | 49.7 |

^1^DHS: Demographic Health Survey; MICS: Multiple Indicator Cluster Survey; ENSANUT: Encuesta Nacional de Salud y Nutrición.

**Supplemental Table 4. Percentage of children at one year of age who were breastfed at the time of the survey by wealth quintiles, slope and concentration inequalities index. Source: DHS, MICS, and ENSANUT, 2010-2017.1**

| **Country** | **Continued breastfeeding at 1 year (12-15 months)** | | | | | | | | | | | | | | | | | | | | | | | | | | |
| --- | --- | --- | --- | --- | --- | --- | --- | --- | --- | --- | --- | --- | --- | --- | --- | --- | --- | --- | --- | --- | --- | --- | --- | --- | --- | --- | --- |
|  | **Poorest** | | | | **Second** | | | | **Third** | | | | **Fourth** | | | | **Wealthiest** | | | | **National prevalence** | **SII^2^** | **95% CI** | | **CIX^3^** | **95% CI** | |
|  | **%** | **95% CI** | | **N** | **%** | **95% CI** | | **N** | **%** | **95% CI** | | **N** | **%** | **95% CI** | | **N** | **%** | **95% CI** | | **N** |  |  |  |  |  |  |  |
| Afghanistan | 86.1 | 81.8 | 89.4 | 403 | 75.1 | 69.6 | 79.8 | 548 | 79.6 | 69.7 | 87.0 | 557 | 77.5 | 71.4 | 82.5 | 571 | 74.5 | 65.5 | 81.9 | 388 | 78.4 | -8.9 | -18.3 | 0.4 | -1.6 | -3.5 | 0.3 |
| Albania | 54.3 | 33.8 | 73.4 | 55 | 63.8 | 49.0 | 76.4 | 56 | 74.5 | 50.1 | 89.4 | 31 | 63.4 | 40.4 | 81.5 | 31 | 32.9 | 10.5 | 67.1 | 15 | 58.4 | -17.4 | -56.4 | 21.6 | -6.0 | -16.9 | 5.0 |
| Algeria | 45.1 | 36.1 | 54.4 | 231 | 47.0 | 37.7 | 56.5 | 197 | 46.4 | 37.6 | 55.4 | 197 | 50.7 | 41.3 | 60.1 | 177 | 44.2 | 35.0 | 53.8 | 145 | 46.7 | 1.9 | -12.7 | 16.5 | 0.0 | -5.1 | 5.2 |
| Angola | 85.8 | 80.6 | 89.8 | 239 | 80.1 | 73.7 | 85.3 | 288 | 87.9 | 82.0 | 92.0 | 225 | 89.0 | 78.8 | 94.6 | 120 | 87.1 | 73.5 | 94.3 | 72 | 85.4 | 5.3 | -4.8 | 15.3 | 0.9 | -1.1 | 3.0 |
| Argentina | 63.3 | 51.3 | 73.8 | 156 | 46.8 | 32.7 | 61.4 | 121 | 43.7 | 28.9 | 59.6 | 113 | 38.6 | 24.8 | 54.5 | 102 | 34.2 | 23.2 | 47.1 | 123 | 46.6 | -35.3 | -55.2 | -15.5 | -12.4 | -20.2 | -4.6 |
| Armenia | 32.2 | 15.6 | 54.8 | 22 | 51.5 | 35.4 | 67.4 | 30 | 31.1 | 16.3 | 51.0 | 21 | 16.4 | 6.9 | 34.5 | 23 | 43.2 | 21.5 | 67.9 | 22 | 36.0 | -6.4 | -43.6 | 30.7 | -2.4 | -21.3 | 16.6 |
| Bangladesh | 97.8 | 90.0 | 99.5 | 125 | 98.4 | 89.1 | 99.8 | 90 | 95.1 | 86.3 | 98.4 | 120 | 93.3 | 81.2 | 97.8 | 107 | 95.7 | 88.7 | 98.4 | 110 | 96.0 | -4.5 | -11.5 | 2.4 | -0.9 | -2.2 | 0.5 |
| Belarus | 32.2 | 13.7 | 58.8 | 26 | 35.3 | 16.4 | 60.2 | 37 | 19.5 | 10.8 | 32.8 | 52 | 21.0 | 10.3 | 38.1 | 37 | 31.5 | 18.5 | 48.1 | 59 | 27.9 | -1.7 | -33.9 | 30.5 | -1.1 | -20.5 | 18.3 |
| Belize | 54.8 | 36.9 | 71.5 | 50 | 59.5 | 40.7 | 75.8 | 52 | 54.2 | 35.5 | 71.8 | 44 | 41.2 | 24.3 | 60.4 | 30 | 40.4 | 23.5 | 59.9 | 33 | 51.5 | -21.4 | -51.6 | 8.9 | -9.1 | -18.4 | 0.1 |
| Benin | 96.1 | 92.2 | 98.1 | 150 | 97.6 | 93.7 | 99.1 | 141 | 97.6 | 92.0 | 99.3 | 153 | 95.9 | 91.1 | 98.2 | 176 | 91.5 | 86.9 | 94.5 | 236 | 95.8 | -5.2 | -10.4 | 0.0 | -0.9 | -1.7 | 0.0 |
| Bhutan | 96.1 | 91.0 | 98.4 | 94 | 91.7 | 83.7 | 96.0 | 107 | 92.3 | 84.4 | 96.3 | 82 | 96.3 | 88.7 | 98.9 | 72 | 87.7 | 76.2 | 94.1 | 68 | 92.7 | -6.6 | -17.0 | 3.8 | -1.0 | -2.8 | 0.9 |
| Burkina Faso | 98.2 | 94.4 | 99.4 | 178 | 95.6 | 92.1 | 97.6 | 216 | 98.7 | 95.7 | 99.6 | 213 | 98.1 | 95.0 | 99.3 | 216 | 92.3 | 85.5 | 96.1 | 163 | 96.8 | -3.2 | -8.2 | 1.8 | -0.6 | -1.5 | 0.3 |
| Burundi | 96.1 | 91.6 | 98.2 | 179 | 95.6 | 91.2 | 97.9 | 195 | 97.2 | 93.3 | 98.8 | 171 | 95.8 | 91.1 | 98.1 | 170 | 89.3 | 82.5 | 93.6 | 179 | 95.0 | -5.7 | -12.0 | 0.6 | -1.1 | -2.1 | -0.1 |
| Cambodia | 84.4 | 74.8 | 90.7 | 120 | 84.7 | 74.3 | 91.4 | 91 | 92.6 | 82.1 | 97.1 | 65 | 82.3 | 69.8 | 90.3 | 73 | 49.9 | 35.6 | 64.2 | 101 | 80.0 | -28.9 | -46.2 | -11.6 | -6.1 | -10.1 | -2.0 |
| Cameroon | 84.9 | 68.7 | 93.5 | 80 | 88.7 | 81.1 | 93.4 | 118 | 71.6 | 59.9 | 80.9 | 100 | 67.0 | 56.3 | 76.2 | 110 | 25.1 | 16.5 | 36.2 | 85 | 70.3 | -60.2 | -75.0 | -45.4 | -14.6 | -18.8 | -10.4 |
| CAR^4^ | 90.7 | 83.7 | 94.8 | 209 | 85.0 | 78.0 | 90.0 | 186 | 91.2 | 84.6 | 95.1 | 171 | 92.0 | 84.6 | 96.0 | 130 | 88.6 | 75.6 | 95.1 | 72 | 89.5 | 1.4 | -9.6 | 12.3 | -0.1 | -2.0 | 1.7 |
| Chad | 88.2 | 81.5 | 92.6 | 218 | 86.3 | 80.5 | 90.6 | 258 | 90.4 | 85.2 | 94.0 | 235 | 90.3 | 85.0 | 93.9 | 226 | 83.2 | 74.9 | 89.2 | 157 | 87.9 | -0.9 | -9.4 | 7.6 | -0.2 | -1.8 | 1.4 |
| Comoros | 75.4 | 61.2 | 85.6 | 51 | 62.1 | 46.4 | 75.6 | 50 | 67.8 | 48.3 | 82.5 | 43 | 75.3 | 51.9 | 89.6 | 30 | 70.2 | 52.0 | 83.7 | 36 | 69.7 | -0.2 | -22.2 | 21.8 | 0.2 | -5.2 | 5.5 |
| Congo Brazzaville | 80.1 | 74.5 | 84.7 | 262 | 73.0 | 60.9 | 82.5 | 148 | 54.1 | 38.3 | 69.1 | 77 | 40.5 | 27.2 | 55.4 | 50 | 53.3 | 30.5 | 74.8 | 30 | 62.1 | -46.7 | -64.2 | -29.2 | -12.8 | -18.9 | -6.6 |
| CDR^5^ | 92.5 | 88.7 | 95.1 | 346 | 96.0 | 92.6 | 97.9 | 296 | 91.1 | 85.0 | 94.9 | 272 | 89.9 | 84.4 | 93.6 | 229 | 88.9 | 81.8 | 93.5 | 151 | 92.0 | -6.0 | -12.5 | 0.5 | -1.2 | -2.4 | 0.0 |
| Costa Rica | 63.1 | 39.0 | 82.1 | 38 | 75.2 | 50.8 | 89.9 | 40 | 56.2 | 21.8 | 85.5 | 23 | 42.4 | 16.1 | 73.8 | 29 | 12.3 | 1.7 | 53.9 | 11 | 54.8 | -56.3 | -92.6 | -19.9 | -18.6 | -32.0 | -5.2 |
| Cote d’Ivoire | 94.7 | 90.6 | 97.0 | 230 | 88.9 | 79.4 | 94.4 | 164 | 90.0 | 77.6 | 95.9 | 132 | 91.5 | 75.3 | 97.4 | 78 | 67.6 | 54.5 | 78.4 | 53 | 88.1 | -22.3 | -35.4 | -9.2 | -4.4 | -6.8 | -2.0 |
| Dominican Republic | 40.1 | 33.0 | 47.7 | 378 | 28.6 | 21.3 | 37.2 | 215 | 29.7 | 20.9 | 40.4 | 202 | 24.4 | 16.0 | 35.4 | 155 | 28.6 | 15.5 | 46.7 | 132 | 31.2 | -15.7 | -32.3 | 0.9 | -9.0 | -18.1 | 0.2 |
| Ecuador | 70.3 | 59.9 | 78.9 | 226 | 65.4 | 53.5 | 75.5 | 177 | 60.9 | 49.0 | 71.6 | 155 | 58.9 | 44.1 | 72.3 | 111 | 64.3 | 48.5 | 77.5 | 78 | 64.4 | -11.2 | -31.0 | 8.5 | -2.8 | -8.3 | 2.7 |
| Egypt | 86.4 | 79.4 | 91.2 | 178 | 77.9 | 69.7 | 84.4 | 171 | 85.0 | 77.8 | 90.2 | 222 | 76.5 | 69.3 | 82.5 | 204 | 72.7 | 65.2 | 79.1 | 243 | 80.0 | -13.2 | -23.4 | -3.0 | -2.9 | -5.0 | -0.8 |
| El Salvador | 87.6 | 75.1 | 94.3 | 114 | 75.7 | 62.9 | 85.1 | 105 | 72.4 | 59.2 | 82.5 | 96 | 68.1 | 51.3 | 81.1 | 84 | 60.0 | 45.6 | 72.8 | 91 | 74.1 | -30.8 | -48.0 | -13.6 | -7.2 | -11.4 | -3.0 |
| Eswatini | 58.6 | 43.5 | 72.2 | 53 | 59.0 | 42.0 | 74.0 | 37 | 51.6 | 35.4 | 67.4 | 42 | 41.1 | 23.9 | 60.7 | 30 | 27.4 | 12.1 | 50.8 | 29 | 47.8 | -38.6 | -63.3 | -13.8 | -14.4 | -23.8 | -4.9 |
| Ethiopia | 91.4 | 86.3 | 94.8 | 290 | 90.7 | 79.9 | 95.9 | 119 | 92.7 | 83.8 | 96.9 | 110 | 94.5 | 84.1 | 98.2 | 96 | 89.1 | 80.1 | 94.4 | 139 | 91.8 | 1.0 | -7.5 | 9.4 | 0.6 | -1.0 | 2.2 |
| Gabon | 64.9 | 56.9 | 72.1 | 192 | 54.1 | 36.4 | 70.8 | 71 | 28.9 | 14.5 | 49.3 | 55 | 44.8 | 29.8 | 60.8 | 35 | 30.2 | 12.0 | 57.9 | 28 | 45.4 | -37.8 | -60.6 | -15.0 | -15.2 | -25.3 | -5.0 |
| Gambia | 96.9 | 90.0 | 99.1 | 171 | 97.6 | 92.8 | 99.2 | 167 | 100.0 | 0.0 | 0.0 | 129 | 98.9 | 94.2 | 99.8 | 103 | 95.2 | 84.3 | 98.6 | 70 | 97.8 | -0.4 | -6.8 | 6.0 | 0.1 | -1.1 | 1.2 |
| Ghana | 98.0 | 92.2 | 99.5 | 132 | 96.6 | 89.4 | 99.0 | 67 | 93.7 | 80.8 | 98.1 | 51 | 90.8 | 74.2 | 97.1 | 61 | 94.0 | 84.1 | 97.9 | 51 | 94.6 | -7.1 | -16.2 | 1.9 | -1.4 | -3.0 | 0.2 |
| Guatemala | 94.2 | 89.5 | 96.8 | 228 | 92.4 | 87.8 | 95.4 | 197 | 84.8 | 76.4 | 90.6 | 174 | 81.4 | 72.9 | 87.7 | 135 | 58.6 | 46.9 | 69.4 | 91 | 85.3 | -33.9 | -44.6 | -23.2 | -6.6 | -8.8 | -4.4 |
| Guinea | 95.4 | 89.5 | 98.1 | 125 | 97.4 | 92.3 | 99.2 | 116 | 94.2 | 88.0 | 97.3 | 108 | 96.7 | 89.6 | 99.0 | 92 | 80.7 | 68.3 | 89.0 | 73 | 93.5 | -13.1 | -24.1 | -2.1 | -2.6 | -4.5 | -0.6 |
| Guinea Bissau | 99.2 | 96.8 | 99.8 | 147 | 96.1 | 90.8 | 98.4 | 156 | 94.9 | 88.2 | 97.9 | 151 | 93.5 | 83.8 | 97.6 | 83 | 84.2 | 69.0 | 92.7 | 36 | 94.6 | -13.5 | -25.1 | -1.9 | -2.2 | -4.0 | -0.4 |
| Guyana | 77.0 | 65.8 | 85.4 | 72 | 53.9 | 34.4 | 72.4 | 42 | 53.1 | 29.5 | 75.4 | 41 | 36.7 | 16.4 | 63.2 | 21 | 35.3 | 19.2 | 55.6 | 28 | 55.6 | -46.4 | -69.3 | -23.5 | -15.0 | -23.7 | -6.3 |
| Haiti | 77.9 | 67.3 | 85.8 | 128 | 82.1 | 71.3 | 89.4 | 100 | 74.4 | 60.2 | 84.8 | 98 | 87.2 | 63.5 | 96.4 | 53 | 62.2 | 47.0 | 75.3 | 46 | 76.9 | -11.0 | -29.3 | 7.2 | -2.5 | -6.4 | 1.4 |
| Honduras | 87.7 | 81.6 | 92.0 | 256 | 77.9 | 70.0 | 84.2 | 187 | 65.9 | 56.5 | 74.1 | 143 | 56.3 | 44.3 | 67.7 | 108 | 48.4 | 35.8 | 61.2 | 75 | 69.6 | -46.4 | -58.4 | -34.4 | -11.6 | -15.2 | -7.9 |
| India | 90.7 | 89.5 | 91.8 | 4293 | 89.4 | 87.9 | 90.7 | 3772 | 85.7 | 83.8 | 87.3 | 3242 | 82.6 | 80.4 | 84.5 | 2725 | 78.8 | 76.0 | 81.3 | 2205 | 86.2 | -14.3 | -17.3 | -11.4 | -2.7 | -3.3 | -2.2 |
| Indonesia | 86.6 | 81.4 | 90.5 | 336 | 79.5 | 72.4 | 85.2 | 273 | 80.1 | 72.0 | 86.3 | 200 | 71.3 | 61.6 | 79.4 | 195 | 69.2 | 59.1 | 77.7 | 169 | 77.6 | -20.8 | -31.7 | -9.9 | -4.2 | -6.6 | -1.9 |
| Iraq | 61.3 | 56.1 | 66.4 | 953 | 52.5 | 46.4 | 58.6 | 631 | 52.0 | 45.3 | 58.6 | 505 | 43.3 | 36.6 | 50.3 | 414 | 42.0 | 34.3 | 50.2 | 262 | 51.5 | -23.5 | -32.9 | -14.0 | -8.1 | -11.2 | -5.0 |
| Jamaica | 44.8 | 26.3 | 64.9 | 37 | 52.5 | 31.3 | 72.8 | 23 | 41.8 | 19.2 | 68.4 | 21 | 46.8 | 24.8 | 70.2 | 21 | 32.3 | 8.0 | 72.2 | 16 | 44.4 | -11.8 | -54.8 | 31.1 | -4.6 | -21.5 | 12.4 |
| Jordan | 36.3 | 27.9 | 45.6 | 222 | 30.2 | 22.1 | 39.8 | 152 | 43.1 | 31.1 | 55.9 | 134 | 24.2 | 11.4 | 44.3 | 51 | 47.3 | 23.0 | 72.9 | 22 | 36.2 | 4.1 | -16.9 | 25.1 | -0.6 | -10.2 | 9.1 |
| Kazakhstan | 52.7 | 39.8 | 65.2 | 76 | 62.1 | 47.2 | 75.1 | 72 | 61.3 | 49.0 | 72.2 | 94 | 56.2 | 42.2 | 69.2 | 75 | 67.5 | 50.3 | 81.0 | 64 | 59.8 | 11.1 | -9.3 | 31.5 | 2.8 | -3.1 | 8.6 |
| Kenya | 90.2 | 85.0 | 93.7 | 253 | 91.0 | 83.1 | 95.4 | 130 | 88.7 | 79.3 | 94.1 | 112 | 88.0 | 74.4 | 94.8 | 103 | 95.3 | 86.7 | 98.4 | 68 | 90.4 | 1.9 | -7.5 | 11.3 | 0.3 | -1.7 | 2.2 |
| Kosovo | 58.1 | 39.0 | 75.0 | 24 | 69.9 | 47.9 | 85.4 | 26 | 42.5 | 22.7 | 65.0 | 19 | 47.2 | 22.4 | 73.4 | 14 | 59.5 | 34.7 | 80.3 | 16 | 56.5 | -12.0 | -46.8 | 22.9 | -3.6 | -14.1 | 6.9 |
| Kyrgyzstan | 63.2 | 45.9 | 77.7 | 57 | 62.0 | 49.0 | 73.5 | 65 | 53.9 | 40.6 | 66.8 | 68 | 60.0 | 40.5 | 76.7 | 56 | 65.0 | 49.6 | 77.7 | 42 | 60.7 | -0.9 | -24.6 | 22.7 | 0.5 | -6.1 | 7.1 |
| Lao | 88.3 | 82.6 | 92.3 | 247 | 84.0 | 76.9 | 89.2 | 181 | 69.4 | 60.5 | 77.1 | 149 | 59.3 | 50.2 | 67.9 | 139 | 51.1 | 39.9 | 62.2 | 86 | 73.0 | -45.7 | -56.5 | -34.9 | -10.6 | -13.5 | -7.8 |
| Lesotho | 88.9 | 78.6 | 94.6 | 73 | 71.1 | 54.2 | 83.6 | 51 | 76.2 | 61.6 | 86.5 | 58 | 60.7 | 39.9 | 78.3 | 38 | 51.3 | 34.3 | 68.1 | 40 | 71.2 | -39.4 | -61.0 | -17.8 | -9.7 | -15.3 | -4.1 |
| Liberia | 95.7 | 91.7 | 97.8 | 167 | 88.6 | 79.7 | 93.9 | 124 | 96.5 | 89.4 | 98.9 | 79 | 82.0 | 68.7 | 90.4 | 63 | 75.0 | 55.7 | 87.8 | 39 | 87.5 | -23.7 | -40.8 | -6.5 | -5.1 | -8.6 | -1.6 |
| Malawi | 93.3 | 89.4 | 95.8 | 269 | 90.3 | 85.4 | 93.7 | 242 | 90.6 | 85.1 | 94.2 | 208 | 90.3 | 84.9 | 94.0 | 204 | 93.1 | 86.7 | 96.5 | 195 | 91.6 | -1.1 | -7.3 | 5.1 | -0.2 | -1.3 | 1.0 |
| Maldives | 88.2 | 76.8 | 94.4 | 51 | 87.6 | 75.5 | 94.2 | 55 | 90.7 | 79.2 | 96.1 | 56 | 79.1 | 50.9 | 93.3 | 28 | 42.7 | 17.8 | 71.8 | 9 | 78.2 | -42.7 | -71.3 | -14.1 | -10.4 | -18.4 | -2.5 |
| Mali | 91.8 | 86.7 | 95.1 | 268 | 92.6 | 88.6 | 95.4 | 271 | 93.9 | 89.5 | 96.5 | 255 | 91.1 | 84.9 | 94.8 | 253 | 91.6 | 86.8 | 94.7 | 243 | 92.3 | -1.0 | -7.4 | 5.3 | -0.3 | -1.4 | 0.8 |
| Mauritania | 81.7 | 73.9 | 87.6 | 197 | 90.5 | 85.4 | 93.9 | 201 | 86.6 | 80.1 | 91.2 | 195 | 92.0 | 84.7 | 96.0 | 163 | 76.1 | 66.4 | 83.7 | 128 | 85.6 | -0.9 | -14.1 | 12.2 | -0.4 | -2.7 | 2.0 |
| Mexico | 70.7 | 58.6 | 80.5 | 165 | 39.8 | 28.5 | 52.2 | 147 | 50.4 | 32.8 | 67.9 | 106 | 24.9 | 13.1 | 42.1 | 76 | 26.6 | 12.9 | 47.0 | 44 | 45.8 | -46.5 | -65.2 | -27.7 | -17.8 | -25.9 | -9.6 |
| Moldova | 67.3 | 43.2 | 84.8 | 21 | 33.6 | 14.4 | 60.3 | 17 | 44.7 | 21.3 | 70.8 | 16 | 44.9 | 22.7 | 69.3 | 19 | 46.3 | 28.2 | 65.4 | 39 | 48.4 | -19.3 | -54.8 | 16.3 | -8.0 | -20.3 | 4.2 |
| Mongolia | 85.5 | 76.0 | 91.6 | 84 | 90.6 | 80.4 | 95.8 | 66 | 89.4 | 80.5 | 94.5 | 78 | 80.7 | 69.6 | 88.4 | 74 | 70.4 | 58.9 | 79.7 | 80 | 82.5 | -22.0 | -37.1 | -6.9 | -4.0 | -7.1 | -1.0 |
| Mozambique | 95.7 | 89.4 | 98.3 | 62 | 95.4 | 88.2 | 98.3 | 57 | 93.6 | 85.7 | 97.3 | 67 | 88.3 | 79.2 | 93.8 | 83 | 81.5 | 68.2 | 90.1 | 73 | 91.5 | -16.4 | -27.7 | -5.0 | -3.0 | -5.1 | -1.0 |
| Myanmar | 92.7 | 83.1 | 97.1 | 99 | 89.1 | 78.1 | 94.9 | 81 | 85.6 | 69.0 | 94.1 | 51 | 85.3 | 71.2 | 93.1 | 61 | 82.0 | 60.5 | 93.1 | 41 | 87.9 | -13.1 | -29.4 | 3.3 | -1.8 | -4.9 | 1.3 |
| Namibia | 73.7 | 59.8 | 84.1 | 69 | 76.3 | 63.3 | 85.7 | 67 | 73.9 | 61.2 | 83.5 | 74 | 52.7 | 37.6 | 67.2 | 60 | 31.2 | 17.2 | 49.7 | 41 | 64.4 | -44.8 | -63.8 | -25.8 | -11.9 | -17.6 | -6.2 |
| Nepal | 97.2 | 89.9 | 99.3 | 90 | 100.0 | 0.0 | 0.0 | 59 | 97.5 | 84.1 | 99.7 | 75 | 97.9 | 91.1 | 99.5 | 75 | 98.3 | 88.6 | 99.8 | 41 | 98.1 | 0.0 | -5.1 | 5.0 | 0.0 | -0.8 | 0.9 |
| Niger | 94.1 | 89.3 | 96.9 | 182 | 93.1 | 87.4 | 96.4 | 176 | 87.3 | 79.9 | 92.3 | 167 | 94.6 | 90.0 | 97.2 | 182 | 93.5 | 88.8 | 96.3 | 219 | 92.6 | 0.0 | -5.6 | 5.6 | -0.1 | -1.1 | 0.9 |
| Nigeria | 90.6 | 86.3 | 93.6 | 474 | 90.7 | 87.5 | 93.2 | 448 | 86.2 | 79.7 | 90.8 | 394 | 81.0 | 76.0 | 85.2 | 372 | 78.5 | 73.2 | 83.0 | 354 | 85.9 | -16.3 | -22.8 | -9.8 | -3.2 | -4.5 | -1.9 |
| Pakistan | 88.6 | 81.7 | 93.2 | 184 | 84.2 | 74.4 | 90.7 | 151 | 77.0 | 65.9 | 85.3 | 154 | 76.0 | 64.0 | 85.0 | 144 | 73.6 | 65.6 | 80.2 | 169 | 80.6 | -19.3 | -30.7 | -8.0 | -3.9 | -6.4 | -1.4 |
| Panama | 69.9 | 57.7 | 79.8 | 205 | 58.2 | 40.1 | 74.3 | 80 | 56.7 | 35.1 | 76.0 | 57 | 29.8 | 14.4 | 51.6 | 48 | 17.9 | 3.3 | 58.0 | 20 | 52.5 | -54.1 | -75.4 | -32.8 | -17.0 | -24.6 | -9.4 |
| Paraguay | 63.5 | 50.3 | 74.9 | 95 | 34.0 | 20.4 | 51.0 | 69 | 57.0 | 41.0 | 71.7 | 52 | 40.6 | 27.3 | 55.5 | 77 | 54.3 | 32.6 | 74.4 | 41 | 48.2 | -7.3 | -34.6 | 20.0 | -2.6 | -12.1 | 6.9 |
| Peru | 87.3 | 81.7 | 91.3 | 330 | 84.4 | 79.3 | 88.4 | 379 | 84.8 | 79.1 | 89.2 | 302 | 77.6 | 70.2 | 83.6 | 248 | 64.9 | 53.5 | 74.9 | 146 | 80.7 | -22.6 | -33.7 | -11.4 | -5.0 | -7.4 | -2.5 |
| Philippines | 75.7 | 66.5 | 83.0 | 211 | 77.6 | 67.4 | 85.3 | 151 | 55.3 | 40.2 | 69.4 | 111 | 59.5 | 46.1 | 71.6 | 85 | 55.9 | 35.7 | 74.3 | 55 | 66.0 | -28.2 | -50.8 | -5.6 | -7.5 | -13.9 | -1.1 |
| Rwanda | 97.4 | 92.3 | 99.2 | 123 | 98.0 | 92.2 | 99.5 | 108 | 98.1 | 92.4 | 99.5 | 94 | 97.3 | 89.6 | 99.4 | 91 | 84.3 | 69.5 | 92.7 | 86 | 95.6 | -11.3 | -23.0 | 0.5 | -1.8 | -3.6 | 0.0 |
| Sao Tome and Principe | 100.0 | 0.0 | 0.0 | 38 | 86.3 | 62.8 | 95.9 | 30 | 100.0 | 0.0 | 0.0 | 19 | 71.9 | 46.1 | 88.5 | 22 | 73.6 | 54.9 | 86.5 | 28 | 85.9 | -35.7 | -55.7 | -15.7 | -7.4 | -11.7 | -3.2 |
| Senegal | 95.9 | 91.7 | 98.0 | 230 | 96.2 | 91.9 | 98.2 | 201 | 92.8 | 87.6 | 95.9 | 150 | 95.0 | 84.9 | 98.5 | 95 | 90.1 | 74.2 | 96.6 | 58 | 94.3 | -6.0 | -15.7 | 3.6 | -1.1 | -2.8 | 0.6 |
| Serbia | 36.6 | 14.1 | 67.0 | 20 | 8.7 | 2.0 | 31.0 | 23 | 20.3 | 8.9 | 39.9 | 32 | 24.6 | 12.0 | 43.7 | 31 | 30.8 | 15.4 | 52.0 | 40 | 24.6 | 7.1 | -28.9 | 43.1 | 4.3 | -20.8 | 29.4 |
| Sierra Leone | 82.4 | 76.3 | 87.2 | 205 | 90.0 | 83.5 | 94.1 | 172 | 88.5 | 81.7 | 93.0 | 165 | 84.8 | 75.8 | 90.9 | 162 | 83.7 | 72.7 | 90.8 | 99 | 86.0 | 1.5 | -8.6 | 11.7 | 0.2 | -1.8 | 2.1 |
| South Africa | 62.3 | 46.5 | 75.8 | 60 | 41.6 | 27.2 | 57.5 | 48 | 44.2 | 27.1 | 62.7 | 44 | 59.9 | 39.9 | 77.0 | 35 | 44.2 | 19.7 | 71.9 | 16 | 51.4 | -8.8 | -39.1 | 21.5 | -3.3 | -13.5 | 6.9 |
| South Sudan | 88.7 | 81.8 | 93.2 | 162 | 84.7 | 77.5 | 89.9 | 165 | 80.2 | 72.1 | 86.4 | 154 | 81.7 | 74.8 | 87.1 | 178 | 74.5 | 64.8 | 82.3 | 140 | 82.3 | -15.3 | -26.4 | -4.1 | -3.1 | -5.4 | -0.9 |
| State of Palestine | 65.6 | 55.7 | 74.3 | 108 | 60.1 | 49.7 | 69.7 | 92 | 48.0 | 39.2 | 56.9 | 115 | 41.8 | 32.7 | 51.6 | 115 | 47.2 | 36.0 | 58.6 | 76 | 52.9 | -28.4 | -43.7 | -13.1 | -8.5 | -13.6 | -3.4 |
| Sudan | 84.7 | 77.9 | 89.7 | 247 | 90.5 | 86.1 | 93.6 | 278 | 91.6 | 86.6 | 94.8 | 242 | 86.7 | 77.5 | 92.5 | 150 | 94.5 | 86.8 | 97.8 | 115 | 89.4 | 7.6 | -1.6 | 16.7 | 1.3 | -0.3 | 3.0 |
| Suriname | 27.5 | 18.5 | 38.8 | 85 | 29.7 | 17.1 | 46.5 | 40 | 14.1 | 4.2 | 38.1 | 22 | 12.9 | 3.9 | 34.9 | 20 | 20.6 | 6.8 | 48.1 | 20 | 22.7 | -17.1 | -41.0 | 6.8 | -17.6 | -35.9 | 0.8 |
| Tajikistan | 79.1 | 67.5 | 87.3 | 81 | 67.8 | 52.3 | 80.2 | 63 | 66.9 | 54.7 | 77.2 | 94 | 80.5 | 70.8 | 87.5 | 83 | 65.9 | 54.2 | 75.9 | 95 | 72.2 | -4.4 | -19.3 | 10.5 | -1.5 | -4.7 | 1.8 |
| Tanzania | 92.0 | 86.8 | 95.3 | 159 | 86.2 | 77.9 | 91.7 | 151 | 95.1 | 89.0 | 97.9 | 127 | 92.7 | 86.7 | 96.2 | 170 | 95.8 | 89.7 | 98.4 | 117 | 92.1 | 6.6 | -0.5 | 13.7 | 1.1 | -0.1 | 2.4 |
| Thailand | 46.6 | 31.8 | 62.0 | 183 | 35.3 | 22.8 | 50.3 | 194 | 34.1 | 16.2 | 57.9 | 153 | 22.1 | 12.0 | 37.3 | 155 | 14.9 | 7.7 | 26.7 | 119 | 33.3 | -35.2 | -57.1 | -13.3 | -17.4 | -28.2 | -6.6 |
| Timor Leste | 74.1 | 63.5 | 82.5 | 93 | 70.3 | 60.3 | 78.6 | 115 | 68.8 | 56.5 | 78.9 | 94 | 49.0 | 37.8 | 60.3 | 105 | 50.4 | 36.0 | 64.8 | 84 | 62.4 | -32.5 | -50.3 | -14.7 | -8.7 | -14.2 | -3.3 |
| Togo | 96.1 | 91.9 | 98.2 | 157 | 93.1 | 85.8 | 96.8 | 101 | 96.0 | 89.6 | 98.6 | 87 | 93.7 | 84.5 | 97.6 | 65 | 90.6 | 81.5 | 95.4 | 67 | 94.0 | -5.1 | -13.4 | 3.3 | -0.8 | -2.4 | 0.7 |
| Tunisia | 61.3 | 42.4 | 77.3 | 43 | 46.0 | 28.9 | 64.1 | 40 | 45.0 | 24.8 | 66.9 | 27 | 57.4 | 38.7 | 74.1 | 34 | 40.0 | 23.1 | 59.7 | 32 | 49.2 | -16.9 | -48.9 | 15.1 | -6.1 | -16.9 | 4.7 |
| Turkmenistan | 82.0 | 66.5 | 91.3 | 40 | 71.3 | 55.4 | 83.2 | 40 | 63.3 | 49.1 | 75.6 | 63 | 61.9 | 47.6 | 74.3 | 69 | 40.3 | 27.5 | 54.7 | 58 | 64.1 | -42.7 | -62.4 | -23.1 | -10.7 | -16.3 | -5.0 |
| Uganda | 94.1 | 90.1 | 96.6 | 248 | 89.4 | 82.6 | 93.7 | 189 | 84.4 | 77.0 | 89.8 | 154 | 85.5 | 78.6 | 90.5 | 165 | 78.6 | 69.5 | 85.6 | 126 | 87.0 | -17.2 | -26.1 | -8.4 | -3.5 | -5.3 | -1.7 |
| Ukraine | 39.2 | 24.4 | 56.3 | 54 | 41.6 | 28.7 | 55.6 | 84 | 39.2 | 17.8 | 65.7 | 47 | 37.8 | 20.7 | 58.6 | 62 | 32.8 | 18.8 | 50.8 | 63 | 37.9 | -9.2 | -34.7 | 16.2 | -5.4 | -17.1 | 6.2 |
| Vietnam | 81.2 | 67.2 | 90.1 | 64 | 78.9 | 63.9 | 88.8 | 47 | 69.5 | 51.9 | 82.8 | 45 | 58.2 | 43.3 | 71.8 | 56 | 38.9 | 25.6 | 54.1 | 53 | 65.6 | -48.4 | -67.3 | -29.4 | -12.9 | -18.6 | -7.3 |
| Yemen | 76.3 | 69.7 | 81.8 | 266 | 69.3 | 62.6 | 75.3 | 265 | 74.1 | 67.1 | 80.1 | 282 | 67.1 | 59.2 | 74.1 | 256 | 67.3 | 57.6 | 75.7 | 196 | 71.2 | -10.0 | -21.0 | 1.0 | -2.4 | -4.9 | 0.1 |
| Zambia | 96.3 | 92.0 | 98.4 | 214 | 97.3 | 94.0 | 98.8 | 213 | 94.2 | 88.5 | 97.2 | 198 | 90.9 | 82.8 | 95.4 | 130 | 77.1 | 65.0 | 85.9 | 108 | 92.2 | -20.4 | -31.4 | -9.3 | -3.7 | -5.5 | -1.8 |
| Zimbabwe | 90.6 | 81.0 | 95.6 | 90 | 94.1 | 85.7 | 97.7 | 79 | 94.9 | 86.1 | 98.3 | 55 | 92.8 | 86.2 | 96.4 | 90 | 79.7 | 63.9 | 89.6 | 65 | 91.1 | -6.8 | -20.2 | 6.7 | -1.5 | -3.8 | 0.8 |

^1^DHS: Demographic Health Survey; MICS: Multiple Indicator Cluster Survey; ENSANUT: Encuesta Nacional de Salud y Nutrición; ^2^SII: slope index of inequality; ^3^CIX: concentration index of inequality; ^4^CAR: Central African Republic; ^5^CDR: Congo Democratic Republic.

**Supplemental Table 5. Average weighted prevalence of continued breastfeeding at 1 years of age by wealth quintiles according to regions and national income groups. Source: DHS, MICS, and ENSANUT, 2010-2017.1**

|  | **Wealth quintiles** | | | | |
| --- | --- | --- | --- | --- | --- |
|  | **Poorest** | **Second** | **Third** | **Fourth** | **Wealthiest** |
| **UNICEF regions** |  |  |  |  |  |
| East Asia & Pacific | 81.2 | 77.1 | 71.0 | 65.0 | 58.5 |
| South Asia | 91.0 | 89.2 | 85.3 | 82.7 | 79.8 |
| Eastern Europe & Central Asia | 53.7 | 52.6 | 49.5 | 50.1 | 49.1 |
| Middle East & North Africa | 73.6 | 69.2 | 72.9 | 66.6 | 66.2 |
| West & Central Africa | 91.7 | 92.1 | 88.2 | 85.7 | 80.4 |
| Eastern & Southern Africa | 89.5 | 85.9 | 87.3 | 88.0 | 83.5 |
| Latin America & Caribbean | 72.8 | 55.3 | 57.9 | 44.3 | 39.9 |
|  |  |  |  |  |  |
| **World Bank income groups** |  |  |  |  |  |
| Upper-middle income | 64.2 | 50.5 | 53.8 | 46.8 | 43.4 |
| Lower-middle income | 88.2 | 86.1 | 82.1 | 78.3 | 74.5 |
| Low income | 92.2 | 90.8 | 91.0 | 90.7 | 86.0 |

^1^DHS: Demographic Health Survey; MICS: Multiple Indicator Cluster Survey; ENSANUT: Encuesta Nacional de Salud y Nutrición.

**Supplemental Table 6. Percentage of children under six months of age who were fed formula at the time of the survey by wealth quintiles, slope and concentration inequalities index. Source: DHS, MICS, and ENSANUT, 2010-2017.1**

| **Country** | **Infant formula consumption under 6 months of age (0-5 months)** | | | | | | | | | | | | | | | | | | | | | | | | | | |
| --- | --- | --- | --- | --- | --- | --- | --- | --- | --- | --- | --- | --- | --- | --- | --- | --- | --- | --- | --- | --- | --- | --- | --- | --- | --- | --- | --- |
|  | **Poorest** | | | | **Second** | | | | **Third** | | | | **Fourth** | | | | **Wealthiest** | | | | **National prevalence** | **SII^2^** | **95% CI** | | **CIX^3^** | **95% CI** | |
|  | **%** | **95% CI** | | **N** | **%** | **95% CI** | | **N** | **%** | **95% CI** | | **N** | **%** | **95% CI** | | **N** | **%** | **95% CI** | | **N** |  |  |  |  |  |  |  |
| Afghanistan | 4.2 | 2.6 | 6.7 | 528 | 7.9 | 4.9 | 12.4 | 754 | 7.4 | 5.0 | 10.6 | 759 | 9.4 | 6.5 | 13.4 | 710 | 13.8 | 9.0 | 20.5 | 452 | 8.5 | 9.3 | 2.8 | 15.8 | 19.0 | 7.1 | 30.9 |
| Albania | 14.7 | 7.4 | 26.9 | 84 | 27.6 | 14.5 | 46.0 | 60 | 18.8 | 7.8 | 38.6 | 55 | 33.8 | 20.5 | 50.4 | 59 | 24.2 | 8.8 | 51.5 | 27 | 24.0 | 14.0 | -10.3 | 38.4 | 13.9 | -2.5 | 30.3 |
| Algeria | 11.5 | 7.6 | 16.9 | 399 | 12.5 | 7.2 | 20.7 | 344 | 8.6 | 5.6 | 12.8 | 316 | 10.7 | 6.9 | 16.2 | 312 | 10.1 | 6.6 | 15.2 | 253 | 10.8 | -2.3 | -9.7 | 5.1 | -2.1 | -13.6 | 9.4 |
| Angola | 1.3 | 0.5 | 3.5 | 413 | 1.7 | 0.7 | 4.1 | 448 | 5.5 | 2.4 | 12.0 | 418 | 12.6 | 7.8 | 19.6 | 211 | 19.7 | 12.7 | 29.2 | 130 | 7.0 | 22.5 | 13.9 | 31.2 | 51.7 | 41.2 | 62.2 |
| Argentina | 29.0 | 21.0 | 38.5 | 214 | 35.6 | 23.9 | 49.3 | 143 | 50.8 | 39.1 | 62.4 | 159 | 57.0 | 44.1 | 69.1 | 149 | 32.8 | 20.8 | 47.5 | 123 | 39.0 | 19.0 | 0.4 | 37.7 | 7.9 | 0.5 | 15.4 |
| Armenia | 7.4 | 2.1 | 23.2 | 28 | 4.5 | 1.1 | 16.4 | 37 | 2.5 | 0.3 | 16.5 | 39 | 2.4 | 0.4 | 14.1 | 35 | 1.7 | 0.2 | 11.1 | 38 | 3.4 | -6.2 | -16.3 | 4.0 | -36.5 | -72.2 | -0.8 |
| Bangladesh | 9.5 | 3.9 | 21.6 | 122 | 3.5 | 1.2 | 9.3 | 130 | 5.9 | 2.2 | 14.7 | 121 | 14.4 | 8.3 | 23.8 | 120 | 17.4 | 10.5 | 27.6 | 139 | 10.1 | 12.7 | 0.0 | 25.5 | 21.8 | 0.1 | 43.5 |
| Belarus | 46.3 | 25.5 | 68.6 | 36 | 57.0 | 38.2 | 74.0 | 51 | 45.5 | 25.8 | 66.7 | 41 | 62.6 | 44.0 | 78.1 | 52 | 43.5 | 29.4 | 58.7 | 67 | 50.3 | -4.6 | -34.8 | 25.5 | -3.6 | -13.4 | 6.2 |
| Belize | 27.9 | 13.0 | 50.1 | 40 | 34.0 | 19.1 | 53.0 | 37 | 51.9 | 29.9 | 73.2 | 34 | 42.9 | 22.3 | 66.3 | 33 | 58.6 | 33.6 | 79.8 | 21 | 42.0 | 32.1 | 2.0 | 62.2 | 12.6 | 0.6 | 24.5 |
| Benin | 0.4 | 0.1 | 2.9 | 221 | 0.0 | 0.0 | 0.0 | 241 | 1.2 | 0.3 | 4.8 | 233 | 1.9 | 0.9 | 3.9 | 294 | 2.5 | 1.5 | 4.2 | 295 | 1.2 | 2.9 | 1.2 | 4.7 | 40.5 | 19.8 | 61.1 |
| Bhutan | 2.8 | 0.5 | 14.0 | 123 | 2.9 | 0.8 | 9.6 | 115 | 5.3 | 2.5 | 10.7 | 149 | 11.6 | 6.0 | 21.1 | 119 | 9.2 | 3.7 | 21.4 | 102 | 6.7 | 10.8 | -0.8 | 22.4 | 24.7 | 0.9 | 48.5 |
| Burkina Faso | 0.0 | 0.0 | 0.0 | 275 | 0.0 | 0.0 | 0.0 | 324 | 0.7 | 0.2 | 2.9 | 329 | 0.0 | 0.0 | 0.0 | 318 | 4.2 | 1.9 | 9.0 | 208 | 0.8 | 3.4 | 0.5 | 6.2 | 77.6 | 52.8 | 102.4 |
| Burundi | 0.0 | 0.0 | 0.0 | 233 | 0.0 | 0.0 | 0.0 | 241 | 0.0 | 0.0 | 0.0 | 222 | 0.0 | 0.0 | 0.0 | 236 | 2.7 | 1.2 | 5.8 | 304 | 0.5 | 2.6 | 0.4 | 4.9 | 95.3 | 85.0 | 105.5 |
| Cambodia | 4.2 | 1.7 | 9.8 | 156 | 5.0 | 2.2 | 10.8 | 121 | 8.3 | 4.3 | 15.4 | 119 | 9.6 | 5.6 | 16.0 | 134 | 34.2 | 24.5 | 45.3 | 158 | 11.5 | 30.0 | 18.0 | 42.0 | 44.8 | 30.6 | 59.0 |
| Cameroon | 0.0 | 0.0 | 0.0 | 117 | 2.0 | 0.8 | 5.0 | 153 | 4.9 | 2.6 | 9.1 | 174 | 13.6 | 6.6 | 26.0 | 147 | 32.3 | 23.7 | 42.4 | 112 | 8.7 | 38.1 | 27.3 | 48.9 | 64.2 | 54.0 | 74.5 |
| CAR^4^ | 1.4 | 0.6 | 3.3 | 277 | 4.4 | 2.4 | 8.2 | 314 | 5.2 | 3.1 | 8.6 | 325 | 7.3 | 3.9 | 13.4 | 233 | 11.2 | 5.9 | 20.4 | 134 | 5.6 | 11.4 | 3.5 | 19.2 | 33.1 | 19.0 | 47.1 |
| Chad | 0.9 | 0.4 | 2.4 | 355 | 0.4 | 0.1 | 0.8 | 356 | 0.7 | 0.3 | 1.9 | 375 | 1.2 | 0.5 | 2.9 | 405 | 21.8 | 17.6 | 26.6 | 332 | 4.7 | 24.9 | 17.8 | 31.9 | 71.7 | 62.9 | 80.5 |
| Comoros | 7.7 | 3.5 | 16.0 | 89 | 30.6 | 18.3 | 46.5 | 66 | 24.0 | 14.7 | 36.6 | 61 | 35.5 | 23.7 | 49.4 | 54 | 43.3 | 28.7 | 59.2 | 57 | 26.5 | 37.7 | 22.5 | 52.9 | 27.4 | 16.7 | 38.2 |
| Congo Brazzaville | 4.6 | 2.7 | 7.7 | 432 | 9.0 | 4.1 | 18.4 | 219 | 10.0 | 4.7 | 20.2 | 112 | 15.6 | 7.6 | 29.3 | 78 | 15.6 | 7.8 | 28.9 | 58 | 10.4 | 14.9 | 3.6 | 26.2 | 23.7 | 10.8 | 36.7 |
| CDR^5^ | 0.2 | 0.0 | 1.4 | 535 | 0.0 | 0.0 | 0.1 | 434 | 0.6 | 0.2 | 2.1 | 369 | 3.1 | 1.2 | 7.3 | 333 | 13.3 | 9.0 | 19.3 | 263 | 3.2 | 16.9 | 9.7 | 24.1 | 76.9 | 66.1 | 87.7 |
| Costa Rica | 34.2 | 20.6 | 51.0 | 90 | 40.2 | 16.2 | 70.1 | 48 | 28.5 | 11.6 | 54.9 | 32 | 65.5 | 36.7 | 86.2 | 24 | 33.1 | 13.5 | 61.1 | 22 | 38.0 | 8.3 | -27.8 | 44.3 | 6.5 | -8.2 | 21.2 |
| Cote d’Ivoire | 0.1 | 0.0 | 0.9 | 316 | 2.2 | 0.5 | 9.6 | 253 | 4.3 | 1.9 | 9.7 | 219 | 10.1 | 3.8 | 24.0 | 119 | 20.1 | 11.4 | 32.9 | 74 | 5.6 | 23.5 | 9.9 | 37.1 | 59.6 | 45.8 | 73.4 |
| Dominican Republic | 22.0 | 16.2 | 29.3 | 552 | 26.5 | 19.7 | 34.7 | 377 | 36.2 | 25.9 | 48.0 | 293 | 30.7 | 23.3 | 39.2 | 253 | 42.2 | 32.0 | 53.1 | 181 | 30.0 | 20.9 | 8.6 | 33.2 | 12.8 | 6.5 | 19.2 |
| Egypt | 15.4 | 11.2 | 20.6 | 299 | 12.2 | 8.3 | 17.5 | 263 | 14.4 | 10.5 | 19.3 | 324 | 16.1 | 11.7 | 21.7 | 314 | 21.3 | 15.8 | 28.0 | 287 | 15.6 | 6.7 | -0.9 | 14.3 | 7.4 | -0.6 | 15.4 |
| El Salvador | 18.7 | 11.5 | 28.9 | 144 | 31.8 | 20.9 | 45.2 | 104 | 35.4 | 23.2 | 49.8 | 98 | 37.9 | 24.8 | 53.0 | 86 | 39.5 | 24.8 | 56.3 | 83 | 31.0 | 26.3 | 7.9 | 44.7 | 15.2 | 5.1 | 25.2 |
| Eswatini | 5.4 | 1.9 | 14.4 | 65 | 6.5 | 2.3 | 16.7 | 59 | 18.8 | 9.7 | 33.2 | 56 | 19.2 | 6.2 | 46.0 | 30 | 37.1 | 19.1 | 59.7 | 25 | 15.1 | 33.2 | 10.7 | 55.6 | 36.6 | 18.3 | 54.8 |
| Ethiopia | 0.2 | 0.0 | 0.9 | 418 | 0.2 | 0.0 | 1.2 | 174 | 0.1 | 0.0 | 0.7 | 129 | 0.1 | 0.0 | 0.4 | 145 | 5.3 | 2.3 | 11.9 | 226 | 0.9 | 4.2 | -0.2 | 8.7 | 75.6 | 52.0 | 99.1 |
| Gabon | 41.6 | 35.0 | 48.5 | 300 | 66.3 | 53.2 | 77.2 | 137 | 73.4 | 60.6 | 83.2 | 88 | 64.5 | 43.8 | 80.9 | 60 | 84.2 | 63.1 | 94.3 | 46 | 65.3 | 40.0 | 22.2 | 57.9 | 11.3 | 6.5 | 16.1 |
| Gambia | 0.5 | 0.1 | 3.8 | 230 | 0.2 | 0.0 | 1.1 | 235 | 0.9 | 0.2 | 3.6 | 192 | 5.8 | 2.8 | 11.7 | 177 | 3.9 | 1.1 | 12.5 | 117 | 2.2 | 6.6 | 1.5 | 11.8 | 49.5 | 31.7 | 67.2 |
| Ghana | 0.0 | 0.0 | 0.0 | 210 | 5.3 | 2.0 | 13.3 | 121 | 10.9 | 5.6 | 20.2 | 112 | 15.6 | 8.9 | 25.8 | 91 | 21.1 | 12.1 | 34.3 | 72 | 9.7 | 27.4 | 14.8 | 39.9 | 43.4 | 31.8 | 55.1 |
| Guatemala | 3.4 | 1.8 | 6.3 | 318 | 8.0 | 5.2 | 11.9 | 287 | 20.3 | 15.0 | 26.8 | 222 | 40.3 | 32.1 | 49.0 | 206 | 56.3 | 46.2 | 65.9 | 142 | 20.9 | 59.4 | 51.5 | 67.3 | 49.2 | 44.0 | 54.4 |
| Guinea | 0.0 | 0.0 | 0.0 | 168 | 3.4 | 1.5 | 7.6 | 162 | 5.6 | 2.3 | 13.2 | 139 | 10.0 | 5.8 | 16.7 | 133 | 21.6 | 13.2 | 33.3 | 84 | 7.2 | 23.9 | 13.6 | 34.3 | 51.1 | 38.0 | 64.2 |
| Guinea Bissau | 0.9 | 0.3 | 2.6 | 260 | 0.6 | 0.1 | 3.9 | 209 | 0.5 | 0.1 | 3.2 | 198 | 1.8 | 0.3 | 11.8 | 94 | 6.9 | 2.6 | 17.1 | 69 | 1.8 | 5.8 | -1.1 | 12.7 | 45.2 | 19.3 | 71.0 |
| Guyana | 19.3 | 10.0 | 34.0 | 114 | 52.7 | 34.8 | 69.8 | 59 | 54.2 | 36.0 | 71.3 | 50 | 59.0 | 36.9 | 77.9 | 33 | 64.0 | 44.7 | 79.6 | 34 | 45.3 | 53.0 | 31.9 | 74.1 | 20.7 | 11.0 | 30.4 |
| Haiti | 8.0 | 4.4 | 14.3 | 200 | 13.4 | 8.4 | 20.7 | 189 | 17.7 | 11.6 | 26.0 | 141 | 26.1 | 17.5 | 36.9 | 105 | 45.7 | 33.0 | 59.1 | 65 | 18.9 | 34.7 | 22.1 | 47.3 | 32.6 | 22.6 | 42.6 |
| Honduras | 5.8 | 3.6 | 9.1 | 341 | 15.0 | 10.6 | 20.8 | 270 | 33.4 | 25.4 | 42.4 | 185 | 39.6 | 31.4 | 48.5 | 178 | 46.4 | 34.9 | 58.2 | 110 | 26.1 | 50.5 | 40.0 | 61.0 | 33.2 | 27.5 | 38.9 |
| India | 2.0 | 1.5 | 2.6 | 5829 | 3.6 | 2.7 | 4.8 | 5494 | 3.9 | 3.1 | 4.9 | 4508 | 4.5 | 3.6 | 5.5 | 3660 | 5.4 | 4.2 | 6.8 | 3135 | 3.7 | 3.8 | 2.3 | 5.2 | 16.7 | 10.2 | 23.3 |
| Indonesia | 19.1 | 14.9 | 24.2 | 510 | 30.1 | 23.9 | 37.0 | 350 | 28.9 | 22.7 | 36.0 | 281 | 46.0 | 37.4 | 54.7 | 310 | 35.2 | 27.4 | 44.0 | 235 | 31.7 | 25.4 | 14.4 | 36.3 | 13.7 | 8.3 | 19.2 |
| Iraq | 31.8 | 28.4 | 35.5 | 1320 | 42.2 | 37.2 | 47.4 | 939 | 43.0 | 37.4 | 48.8 | 691 | 48.5 | 42.6 | 54.5 | 533 | 56.7 | 49.2 | 64.0 | 399 | 43.5 | 27.1 | 18.7 | 35.4 | 11.4 | 8.4 | 14.5 |
| Jamaica | 37.2 | 21.9 | 55.6 | 28 | 30.9 | 17.3 | 48.9 | 39 | 68.9 | 53.1 | 81.3 | 47 | 59.9 | 39.4 | 77.5 | 30 | 65.5 | 40.3 | 84.2 | 23 | 52.9 | 42.3 | 15.6 | 69.0 | 13.8 | 4.3 | 23.3 |
| Jordan | 40.0 | 32.5 | 48.1 | 441 | 48.2 | 40.2 | 56.3 | 312 | 55.5 | 45.7 | 64.8 | 241 | 53.9 | 44.8 | 62.7 | 164 | 55.2 | 37.7 | 71.5 | 60 | 49.5 | 19.5 | 3.9 | 35.2 | 7.6 | 2.4 | 12.7 |
| Kazakhstan | 14.3 | 8.3 | 23.7 | 82 | 25.0 | 14.2 | 40.1 | 90 | 22.9 | 15.2 | 33.1 | 142 | 12.8 | 7.5 | 21.1 | 102 | 23.1 | 11.7 | 40.5 | 92 | 20.0 | 1.7 | -15.5 | 18.8 | 1.0 | -12.4 | 14.5 |
| Kenya | 0.8 | 0.1 | 4.8 | 320 | 0.0 | 0.0 | 0.0 | 167 | 0.9 | 0.2 | 3.8 | 140 | 0.8 | 0.2 | 3.2 | 119 | 0.5 | 0.1 | 2.4 | 110 | 0.6 | 0.0 | -1.9 | 1.9 | -5.9 | -67.1 | 55.2 |
| Kosovo | 21.2 | 10.7 | 37.6 | 38 | 18.9 | 7.4 | 40.5 | 24 | 22.5 | 9.9 | 43.5 | 28 | 38.4 | 20.0 | 60.8 | 25 | 20.3 | 8.3 | 41.7 | 30 | 23.8 | 7.1 | -18.6 | 32.7 | 5.0 | -12.8 | 22.8 |
| Kyrgyzstan | 3.7 | 1.4 | 9.5 | 89 | 4.9 | 1.9 | 11.9 | 97 | 7.4 | 2.9 | 17.8 | 93 | 13.9 | 6.6 | 26.8 | 79 | 10.2 | 4.5 | 21.3 | 74 | 7.9 | 10.5 | 0.6 | 20.3 | 21.9 | 2.8 | 41.0 |
| Lao | 2.5 | 0.8 | 7.7 | 325 | 3.1 | 1.6 | 6.2 | 273 | 8.6 | 5.2 | 13.9 | 253 | 14.1 | 9.3 | 20.9 | 175 | 27.5 | 19.7 | 36.9 | 142 | 9.7 | 28.5 | 19.0 | 38.0 | 51.1 | 40.3 | 61.8 |
| Lesotho | 4.4 | 1.7 | 11.0 | 82 | 9.2 | 4.1 | 19.4 | 76 | 10.9 | 4.9 | 22.4 | 67 | 23.5 | 13.0 | 38.8 | 59 | 49.3 | 27.1 | 71.8 | 43 | 17.4 | 46.8 | 24.9 | 68.6 | 42.4 | 27.3 | 57.4 |
| Liberia | 0.9 | 0.2 | 3.4 | 256 | 0.4 | 0.1 | 1.7 | 194 | 6.1 | 2.1 | 16.3 | 148 | 9.9 | 4.1 | 22.1 | 77 | 12.6 | 5.1 | 27.9 | 42 | 5.1 | 16.5 | 5.6 | 27.4 | 50.7 | 33.4 | 67.9 |
| Malawi | 2.6 | 1.0 | 6.4 | 379 | 0.9 | 0.3 | 2.4 | 358 | 0.7 | 0.2 | 2.2 | 317 | 2.7 | 1.0 | 7.4 | 293 | 2.8 | 1.0 | 8.0 | 289 | 1.9 | 0.6 | -2.8 | 4.0 | 7.8 | -22.2 | 37.7 |
| Maldives | 22.2 | 13.8 | 33.7 | 86 | 13.0 | 6.6 | 23.7 | 82 | 7.0 | 3.2 | 14.5 | 73 | 15.6 | 4.1 | 44.2 | 28 | 26.1 | 9.2 | 55.0 | 19 | 17.0 | 5.7 | -24.8 | 36.3 | -1.0 | -29.1 | 27.1 |
| Mali | 0.6 | 0.1 | 2.2 | 345 | 0.7 | 0.2 | 2.2 | 362 | 0.5 | 0.2 | 1.6 | 357 | 2.2 | 1.0 | 4.6 | 303 | 4.1 | 2.4 | 7.2 | 296 | 1.5 | 4.1 | 1.4 | 6.8 | 43.8 | 22.5 | 65.2 |
| Mauritania | 2.5 | 1.0 | 6.2 | 198 | 3.7 | 1.4 | 9.5 | 220 | 5.7 | 3.1 | 10.5 | 190 | 18.7 | 10.9 | 30.1 | 147 | 30.9 | 21.7 | 41.9 | 160 | 10.7 | 37.0 | 23.8 | 50.2 | 51.1 | 38.7 | 63.4 |
| Mexico | 40.4 | 29.9 | 51.7 | 189 | 49.6 | 38.9 | 60.3 | 203 | 41.0 | 23.7 | 60.8 | 134 | 63.7 | 47.9 | 77.0 | 93 | 66.3 | 45.9 | 82.0 | 47 | 48.8 | 20.8 | -0.5 | 42.1 | 8.1 | 1.3 | 14.9 |
| Moldova | 4.6 | 1.1 | 17.4 | 21 | 19.5 | 9.5 | 36.0 | 38 | 27.7 | 10.4 | 55.9 | 27 | 24.3 | 12.4 | 42.3 | 34 | 26.6 | 16.7 | 39.6 | 56 | 21.2 | 21.6 | 3.4 | 39.9 | 17.5 | 2.9 | 32.1 |
| Mongolia | 3.3 | 1.4 | 7.8 | 146 | 7.7 | 4.3 | 13.4 | 150 | 18.4 | 11.8 | 27.7 | 129 | 28.4 | 20.0 | 38.7 | 108 | 32.8 | 24.0 | 43.0 | 111 | 17.4 | 39.7 | 28.7 | 50.8 | 36.2 | 27.9 | 44.5 |
| Myanmar | 2.0 | 0.5 | 7.8 | 119 | 10.9 | 5.3 | 21.3 | 101 | 4.1 | 1.2 | 12.7 | 87 | 3.9 | 1.3 | 11.4 | 87 | 11.7 | 4.9 | 25.2 | 74 | 6.2 | 6.9 | -3.1 | 16.8 | 18.7 | -5.5 | 42.9 |
| Namibia | 6.8 | 3.1 | 14.0 | 122 | 6.8 | 3.1 | 14.4 | 124 | 11.0 | 5.3 | 21.4 | 116 | 29.5 | 19.9 | 41.3 | 97 | 35.4 | 24.1 | 48.5 | 66 | 15.6 | 35.6 | 22.0 | 49.2 | 37.6 | 25.4 | 49.7 |
| Nepal | 1.0 | 0.1 | 6.5 | 131 | 3.4 | 0.9 | 12.4 | 88 | 0.0 | 0.0 | 0.0 | 98 | 5.4 | 2.0 | 13.5 | 82 | 15.7 | 7.0 | 31.6 | 68 | 4.5 | 15.8 | 1.8 | 29.9 | 53.3 | 30.6 | 76.0 |
| Niger | 0.2 | 0.0 | 1.3 | 268 | 0.2 | 0.0 | 1.7 | 241 | 0.0 | 0.0 | 0.0 | 243 | 0.9 | 0.2 | 5.4 | 264 | 5.7 | 3.1 | 10.0 | 287 | 1.2 | 5.2 | 1.7 | 8.8 | 71.4 | 51.6 | 91.1 |
| Nigeria | 0.8 | 0.4 | 1.7 | 611 | 2.6 | 1.6 | 4.3 | 638 | 5.2 | 3.0 | 8.8 | 525 | 11.5 | 8.0 | 16.2 | 551 | 15.1 | 11.8 | 19.2 | 423 | 6.4 | 18.8 | 14.2 | 23.5 | 46.5 | 39.3 | 53.6 |
| Pakistan | 4.2 | 1.8 | 9.5 | 233 | 4.5 | 2.1 | 9.4 | 199 | 8.6 | 4.9 | 14.5 | 212 | 8.7 | 5.4 | 13.6 | 217 | 26.8 | 18.7 | 36.8 | 214 | 9.9 | 21.8 | 12.6 | 31.1 | 36.7 | 23.1 | 50.2 |
| Panama | 18.8 | 12.7 | 27.0 | 285 | 45.0 | 29.6 | 61.5 | 86 | 80.9 | 62.8 | 91.4 | 67 | 71.4 | 46.4 | 87.8 | 42 | 78.6 | 53.7 | 92.1 | 30 | 55.0 | 71.3 | 54.6 | 87.9 | 27.0 | 21.1 | 32.9 |
| Paraguay | 13.7 | 7.5 | 23.8 | 105 | 23.2 | 13.5 | 36.9 | 92 | 31.8 | 20.1 | 46.3 | 80 | 44.7 | 28.0 | 62.7 | 68 | 44.1 | 25.7 | 64.2 | 49 | 28.9 | 40.9 | 21.2 | 60.5 | 25.9 | 15.6 | 36.2 |
| Peru | 3.5 | 2.0 | 6.2 | 399 | 14.3 | 10.5 | 19.2 | 401 | 22.0 | 16.4 | 28.9 | 280 | 29.2 | 21.9 | 37.7 | 213 | 38.4 | 28.4 | 49.5 | 153 | 19.0 | 41.3 | 31.3 | 51.2 | 37.2 | 30.7 | 43.7 |
| Rwanda | 0.0 | 0.0 | 0.0 | 152 | 0.0 | 0.0 | 0.0 | 141 | 1.0 | 0.1 | 6.8 | 124 | 0.0 | 0.0 | 0.0 | 131 | 2.1 | 0.8 | 5.2 | 155 | 0.6 | 2.1 | 0.2 | 4.1 | 69.0 | 28.0 | 109.9 |
| Sao Tome and Principe | 2.4 | 0.3 | 16.1 | 44 | 6.8 | 1.3 | 27.9 | 35 | 6.4 | 1.5 | 23.2 | 40 | 0.8 | 0.1 | 5.7 | 35 | 5.9 | 0.8 | 33.0 | 15 | 4.5 | -0.7 | -11.2 | 9.7 | -5.5 | -44.8 | 33.8 |
| Senegal | 1.2 | 0.3 | 4.1 | 350 | 1.4 | 0.5 | 3.8 | 306 | 7.5 | 4.2 | 13.0 | 244 | 5.4 | 2.3 | 12.0 | 151 | 16.2 | 9.0 | 27.4 | 91 | 5.3 | 15.6 | 6.5 | 24.6 | 46.7 | 32.1 | 61.3 |
| Serbia | 23.6 | 7.5 | 54.2 | 17 | 54.4 | 29.6 | 77.1 | 23 | 33.6 | 17.6 | 54.4 | 40 | 61.0 | 33.5 | 82.9 | 39 | 26.6 | 14.1 | 44.5 | 50 | 40.9 | -9.0 | -39.5 | 21.5 | -3.1 | -16.6 | 10.4 |
| Sierra Leone | 1.2 | 0.3 | 3.9 | 246 | 2.7 | 0.7 | 9.9 | 215 | 4.3 | 2.1 | 8.4 | 266 | 6.1 | 2.9 | 12.3 | 229 | 20.2 | 14.4 | 27.6 | 159 | 5.9 | 18.5 | 10.8 | 26.3 | 46.8 | 31.4 | 62.2 |
| South Africa | 30.2 | 19.1 | 44.4 | 68 | 23.8 | 15.3 | 35.0 | 86 | 41.1 | 29.6 | 53.6 | 89 | 29.4 | 17.6 | 44.7 | 60 | 31.8 | 17.2 | 51.2 | 43 | 31.4 | 4.7 | -17.1 | 26.5 | 2.7 | -8.8 | 14.2 |
| South Sudan | 5.4 | 2.7 | 10.5 | 156 | 7.0 | 3.6 | 13.4 | 164 | 3.9 | 1.5 | 9.8 | 166 | 9.9 | 6.4 | 15.1 | 195 | 12.2 | 8.2 | 17.8 | 196 | 7.9 | 8.6 | 1.2 | 16.1 | 18.7 | 4.0 | 33.3 |
| State of Palestine | 39.6 | 31.0 | 48.8 | 154 | 45.1 | 35.6 | 55.0 | 128 | 36.1 | 27.9 | 45.3 | 121 | 40.7 | 32.1 | 49.9 | 154 | 38.4 | 29.6 | 48.1 | 108 | 40.2 | -2.6 | -17.6 | 12.4 | -0.6 | -6.9 | 5.7 |
| Sudan | 2.6 | 1.4 | 4.8 | 367 | 6.3 | 3.3 | 11.9 | 435 | 1.8 | 1.0 | 3.5 | 352 | 1.1 | 0.4 | 3.1 | 223 | 6.7 | 3.1 | 13.8 | 166 | 3.6 | 0.2 | -4.4 | 4.7 | -1.5 | -24.4 | 21.4 |
| Suriname | 67.0 | 57.9 | 74.9 | 173 | 77.8 | 61.3 | 88.6 | 38 | 81.6 | 64.6 | 91.5 | 39 | 76.1 | 58.5 | 87.7 | 36 | 80.4 | 57.9 | 92.4 | 18 | 74.1 | 19.3 | -3.4 | 42.0 | 5.5 | 1.2 | 9.7 |
| Tajikistan | 6.4 | 3.1 | 12.7 | 99 | 5.4 | 2.1 | 13.1 | 79 | 10.9 | 6.5 | 17.7 | 112 | 11.4 | 6.6 | 19.0 | 121 | 12.7 | 7.6 | 20.4 | 142 | 9.7 | 8.9 | 0.3 | 17.5 | 13.9 | 0.3 | 27.4 |
| Tanzania | 0.0 | 0.0 | 0.0 | 258 | 0.1 | 0.0 | 0.6 | 209 | 0.0 | 0.0 | 0.0 | 179 | 0.3 | 0.0 | 2.5 | 214 | 4.1 | 1.5 | 10.9 | 155 | 0.7 | 3.3 | -0.1 | 6.8 | 86.0 | 72.6 | 99.5 |
| Thailand | 37.7 | 22.7 | 55.4 | 151 | 40.4 | 26.3 | 56.4 | 153 | 41.4 | 26.6 | 57.9 | 136 | 61.0 | 44.0 | 75.6 | 130 | 36.0 | 19.3 | 56.8 | 91 | 44.9 | 14.1 | -12.0 | 40.2 | 6.5 | -3.3 | 16.4 |
| Timor Leste | 2.1 | 0.6 | 7.5 | 126 | 4.0 | 1.7 | 9.1 | 150 | 9.2 | 4.6 | 17.4 | 158 | 12.2 | 7.2 | 20.0 | 160 | 18.3 | 11.1 | 28.5 | 149 | 9.5 | 20.4 | 10.1 | 30.7 | 38.4 | 24.5 | 52.3 |
| Togo | 0.0 | 0.0 | 0.0 | 199 | 1.2 | 0.3 | 5.1 | 114 | 1.7 | 0.2 | 11.3 | 102 | 1.9 | 0.5 | 7.6 | 97 | 6.0 | 2.5 | 13.6 | 91 | 2.1 | 6.8 | 0.7 | 13.0 | 51.9 | 25.4 | 78.5 |
| Tunisia | 10.5 | 4.7 | 21.7 | 78 | 3.7 | 1.1 | 11.6 | 65 | 8.5 | 3.1 | 21.2 | 50 | 9.9 | 4.6 | 20.1 | 75 | 14.8 | 5.5 | 34.4 | 38 | 9.5 | 7.4 | -8.1 | 22.9 | 15.6 | -10.7 | 41.9 |
| Turkmenistan | 16.4 | 7.9 | 30.9 | 49 | 6.4 | 2.4 | 16.4 | 59 | 12.7 | 6.1 | 24.4 | 70 | 5.6 | 2.4 | 12.6 | 84 | 21.9 | 13.1 | 34.2 | 80 | 12.3 | 4.8 | -11.1 | 20.7 | 3.7 | -17.4 | 24.7 |
| Uganda | 0.0 | 0.0 | 0.3 | 400 | 0.1 | 0.0 | 0.4 | 331 | 0.4 | 0.1 | 2.3 | 286 | 0.7 | 0.1 | 4.5 | 255 | 1.7 | 0.5 | 5.7 | 210 | 0.5 | 1.8 | -0.2 | 3.8 | 69.7 | 42.8 | 96.6 |
| Ukraine | 26.1 | 16.4 | 39.0 | 65 | 29.9 | 17.2 | 46.6 | 76 | 19.0 | 9.2 | 35.2 | 49 | 41.2 | 25.4 | 59.2 | 57 | 32.8 | 16.6 | 54.5 | 60 | 30.5 | 10.1 | -17.9 | 38.2 | 3.5 | -11.6 | 18.6 |
| Vietnam | 13.8 | 6.8 | 26.1 | 89 | 36.3 | 24.7 | 49.7 | 68 | 39.9 | 27.5 | 53.7 | 74 | 51.0 | 37.3 | 64.6 | 68 | 49.9 | 36.8 | 63.0 | 59 | 37.5 | 41.2 | 24.9 | 57.4 | 20.8 | 13.2 | 28.3 |
| Yemen | 20.9 | 16.7 | 25.9 | 366 | 29.9 | 24.0 | 36.6 | 376 | 33.4 | 26.8 | 40.8 | 360 | 33.8 | 27.4 | 40.9 | 343 | 51.9 | 42.8 | 60.8 | 227 | 32.8 | 28.8 | 18.9 | 38.6 | 14.9 | 9.9 | 19.8 |
| Zambia | 0.3 | 0.0 | 1.8 | 288 | 0.0 | 0.0 | 0.0 | 309 | 0.3 | 0.0 | 2.1 | 251 | 1.6 | 0.6 | 4.2 | 203 | 10.4 | 5.6 | 18.4 | 138 | 1.8 | 9.2 | 3.2 | 15.1 | 74.4 | 59.1 | 89.8 |
| Zimbabwe | 0.6 | 0.1 | 4.0 | 120 | 0.0 | 0.0 | 0.0 | 117 | 0.0 | 0.0 | 0.0 | 99 | 4.9 | 2.3 | 10.1 | 161 | 10.4 | 4.5 | 22.0 | 106 | 2.8 | 12.3 | 3.4 | 21.1 | 62.1 | 43.2 | 81.1 |

^1^DHS: Demographic Health Survey; MICS: Multiple Indicator Cluster Survey; ENSANUT: Encuesta Nacional de Salud y Nutrición; ^2^SII: slope index of inequality; ^3^CIX: concentration index of inequality; ^4^CAR: Central African Republic; ^5^CDR: Congo Democratic Republic.

**Supplemental Table 7. Average weighted prevalence of infant formula consumption under 6 months of age by wealth quintiles according to regions and national income. Source: DHS, MICS, and ENSANUT, 2010-2017.1**

|  | **Wealth quintiles** | | | | |
| --- | --- | --- | --- | --- | --- |
|  | **Poorest** | **Second** | **Third** | **Fourth** | **Wealthiest** |
| **UNICEF regions** |  |  |  |  |  |
| East Asia & Pacific | 14.9 | 24.9 | 24.7 | 36.5 | 31.2 |
| South Asia | 3.1 | 3.8 | 4.8 | 6.2 | 10.1 |
| Eastern Europe & Central Asia | 18.0 | 23.4 | 19.6 | 26.6 | 24.3 |
| Middle East & North Africa | 16.7 | 19.2 | 19.5 | 21.2 | 27.5 |
| West & Central Africa | 0.7 | 2.1 | 4.1 | 8.4 | 15.3 |
| Eastern & Southern Africa | 3.1 | 2.6 | 4.4 | 4.7 | 8.3 |
| Latin America & Caribbean | 26.0 | 33.5 | 35.2 | 49.0 | 49.6 |
|  |  |  |  |  |  |
| **World Bank income groups** |  |  |  |  |  |
| Upper-middle income | 24.3 | 28.9 | 31.2 | 39.0 | 37.9 |
| Lower-middle income | 5.8 | 10.3 | 9.7 | 13.6 | 16.8 |
| Low income | 0.9 | 1.3 | 1.7 | 3.0 | 9.0 |

^1^DHS: Demographic Health Survey; MICS: Multiple Indicator Cluster Survey; ENSANUT: Encuesta Nacional de Salud y Nutrición.

**Supplemental Table 8. Percentage of children under six months of age who were fed non-human milk other than formula at the time of the survey by wealth quintiles, slope and concentration inequalities index. Source: DHS, MICS, and ENSANUT, 2010-2017.1**

| **Country** | **Consumption of other non-human milks under 6 months of age (0-5 months)** | | | | | | | | | | | | | | | | | | | | | | | | | | |
| --- | --- | --- | --- | --- | --- | --- | --- | --- | --- | --- | --- | --- | --- | --- | --- | --- | --- | --- | --- | --- | --- | --- | --- | --- | --- | --- | --- |
|  | **Poorest** | | | | **Second** | | | | **Third** | | | | **Fourth** | | | | **Wealthiest** | | | | **National prevalence** | **SII^2^** | **95% CI** | | **CIX^3^** | **95% CI** | |
|  | **%** | **95% CI** | | **N** | **%** | **95% CI** | | **N** | **%** | **95% CI** | | **N** | **%** | **95% CI** | | **N** | **%** | **95% CI** | | **N** |  |  |  |  |  |  |  |
| Afghanistan | 40.6 | 34.0 | 47.7 | 528 | 34.7 | 27.4 | 42.7 | 754 | 31.9 | 26.4 | 38.0 | 759 | 28.0 | 20.8 | 36.5 | 710 | 28.0 | 20.0 | 37.9 | 452 | 32.5 | -14.1 | -27.1 | -1.2 | -8.6 | -15.3 | -1.8 |
| Albania | 17.3 | 9.6 | 29.1 | 84 | 19.8 | 8.6 | 39.4 | 60 | 17.6 | 7.0 | 37.7 | 55 | 21.0 | 10.4 | 37.7 | 59 | 19.0 | 5.9 | 47.0 | 27 | 19.0 | 2.6 | -20.2 | 25.5 | 4.1 | -15.8 | 24.1 |
| Algeria | 39.9 | 33.0 | 47.3 | 399 | 34.7 | 28.7 | 41.3 | 344 | 41.0 | 34.3 | 48.1 | 316 | 45.4 | 38.2 | 52.8 | 312 | 37.8 | 30.9 | 45.2 | 253 | 39.6 | 3.3 | -7.5 | 14.0 | 2.3 | -2.2 | 6.9 |
| Angola | 2.4 | 1.3 | 4.3 | 413 | 0.9 | 0.3 | 3.1 | 448 | 3.9 | 1.3 | 11.2 | 418 | 7.8 | 3.2 | 17.7 | 211 | 6.7 | 2.9 | 14.9 | 130 | 4.0 | 7.7 | 1.3 | 14.0 | 32.2 | 14.3 | 50.1 |
| Argentina | 16.6 | 10.9 | 24.6 | 214 | 19.0 | 9.9 | 33.4 | 143 | 2.1 | 0.9 | 4.8 | 159 | 11.4 | 5.1 | 23.6 | 149 | 1.6 | 0.5 | 4.7 | 123 | 11.0 | -21.4 | -33.9 | -8.9 | -28.6 | -43.1 | -14.0 |
| Armenia | 17.2 | 6.7 | 37.5 | 28 | 16.8 | 7.5 | 33.7 | 37 | 13.6 | 5.7 | 28.8 | 39 | 7.0 | 2.0 | 21.9 | 35 | 23.4 | 12.0 | 40.8 | 38 | 16.1 | 5.2 | -16.8 | 27.2 | 5.1 | -19.2 | 29.4 |
| Bangladesh | 11.6 | 5.4 | 23.2 | 122 | 9.9 | 4.7 | 19.4 | 130 | 6.6 | 2.8 | 14.9 | 121 | 4.9 | 2.2 | 10.5 | 120 | 8.5 | 3.1 | 21.5 | 139 | 8.4 | -5.4 | -18.1 | 7.3 | -13.7 | -37.7 | 10.4 |
| Belarus | 8.1 | 1.1 | 40.7 | 36 | 5.3 | 1.8 | 14.3 | 51 | 0.0 | 0.0 | 0.0 | 41 | 2.3 | 0.3 | 15.1 | 52 | 4.1 | 1.4 | 11.5 | 67 | 3.8 | -3.7 | -18.7 | 11.2 | -12.4 | -70.1 | 45.4 |
| Belize | 11.8 | 4.1 | 29.5 | 40 | 15.7 | 6.2 | 34.4 | 37 | 9.8 | 3.9 | 22.4 | 34 | 4.4 | 1.3 | 13.8 | 33 | 13.7 | 4.3 | 36.2 | 21 | 11.1 | -5.4 | -25.2 | 14.4 | -10.4 | -39.9 | 19.2 |
| Benin | 6.8 | 3.7 | 12.1 | 221 | 1.1 | 0.3 | 3.7 | 241 | 3.2 | 1.4 | 7.2 | 233 | 4.3 | 1.8 | 9.9 | 294 | 5.2 | 2.8 | 9.5 | 295 | 4.0 | 0.2 | -4.9 | 5.4 | 0.6 | -23.5 | 24.6 |
| Bhutan | 9.6 | 4.2 | 20.4 | 123 | 4.8 | 1.6 | 13.1 | 115 | 7.2 | 3.4 | 14.7 | 149 | 4.4 | 2.0 | 9.8 | 119 | 2.3 | 0.4 | 11.8 | 102 | 5.6 | -8.1 | -17.7 | 1.4 | -23.6 | -45.2 | -2.0 |
| Burkina Faso | 2.2 | 0.8 | 6.0 | 275 | 1.5 | 0.6 | 3.7 | 324 | 0.9 | 0.2 | 3.4 | 329 | 0.8 | 0.2 | 3.3 | 318 | 1.4 | 0.4 | 5.6 | 208 | 1.3 | -1.2 | -4.1 | 1.6 | -15.7 | -49.3 | 18.0 |
| Burundi | 0.0 | 0.0 | 0.0 | 233 | 0.4 | 0.1 | 3.0 | 241 | 0.6 | 0.1 | 4.0 | 222 | 0.1 | 0.0 | 0.9 | 236 | 6.5 | 3.6 | 11.5 | 304 | 1.4 | 6.7 | 2.0 | 11.3 | 72.9 | 54.4 | 91.4 |
| Cambodia | 2.8 | 0.8 | 9.8 | 156 | 1.9 | 0.5 | 6.5 | 121 | 4.1 | 1.7 | 9.6 | 119 | 2.5 | 0.9 | 7.2 | 134 | 7.5 | 3.4 | 15.9 | 158 | 3.7 | 4.5 | -2.3 | 11.3 | 17.5 | -8.7 | 43.7 |
| Cameroon | 4.6 | 1.4 | 13.6 | 117 | 2.5 | 0.6 | 9.9 | 153 | 3.8 | 1.5 | 8.9 | 174 | 3.1 | 1.5 | 6.6 | 147 | 15.4 | 8.6 | 26.0 | 112 | 5.3 | 8.3 | -1.3 | 17.9 | 23.3 | -5.3 | 51.8 |
| CAR^4^ | 0.1 | 0.0 | 0.8 | 277 | 0.1 | 0.0 | 0.5 | 314 | 0.8 | 0.2 | 2.8 | 325 | 2.9 | 1.1 | 7.3 | 233 | 7.2 | 3.2 | 15.4 | 134 | 1.9 | 9.0 | 2.2 | 15.8 | 67.3 | 51.8 | 82.9 |
| Chad | 6.2 | 3.9 | 9.7 | 355 | 11.0 | 7.5 | 15.8 | 356 | 10.9 | 7.7 | 15.1 | 375 | 15.4 | 11.6 | 20.3 | 405 | 18.7 | 13.7 | 25.0 | 332 | 12.2 | 14.5 | 7.7 | 21.3 | 21.0 | 12.0 | 29.9 |
| Comoros | 4.1 | 1.4 | 11.1 | 89 | 23.3 | 12.1 | 40.2 | 66 | 12.3 | 5.4 | 25.6 | 61 | 13.2 | 6.5 | 24.9 | 54 | 27.4 | 17.5 | 40.1 | 57 | 15.2 | 18.6 | 5.2 | 32.0 | 20.7 | 4.6 | 36.9 |
| Congo Brazzaville | 5.2 | 3.3 | 8.0 | 432 | 12.5 | 6.7 | 22.2 | 219 | 14.8 | 7.3 | 27.5 | 112 | 27.3 | 16.6 | 41.4 | 78 | 16.7 | 8.3 | 30.7 | 58 | 14.7 | 21.7 | 9.1 | 34.2 | 24.9 | 14.7 | 35.1 |
| CDR^5^ | 0.8 | 0.3 | 2.7 | 535 | 0.4 | 0.1 | 1.3 | 434 | 3.0 | 0.7 | 11.5 | 369 | 2.5 | 1.0 | 6.2 | 333 | 7.6 | 4.0 | 14.2 | 263 | 2.7 | 8.0 | 2.4 | 13.6 | 46.7 | 28.3 | 65.2 |
| Costa Rica | 17.9 | 7.7 | 36.4 | 90 | 18.6 | 3.6 | 57.9 | 48 | 1.5 | 0.2 | 10.1 | 32 | 4.8 | 0.6 | 28.3 | 24 | 15.3 | 2.1 | 60.8 | 22 | 13.3 | -12.2 | -46.7 | 22.4 | -14.7 | -51.1 | 21.7 |
| Cote d’Ivoire | 2.1 | 0.8 | 5.0 | 316 | 1.9 | 0.5 | 6.9 | 253 | 6.1 | 3.0 | 12.0 | 219 | 12.3 | 5.2 | 26.3 | 119 | 13.7 | 6.3 | 27.4 | 74 | 6.0 | 16.2 | 3.6 | 28.7 | 44.2 | 27.6 | 60.8 |
| Dominican Republic | 55.2 | 49.3 | 61.0 | 552 | 62.8 | 54.2 | 70.7 | 377 | 56.6 | 45.6 | 66.9 | 293 | 66.9 | 58.5 | 74.4 | 253 | 61.2 | 50.5 | 71.0 | 181 | 60.1 | 8.5 | -3.4 | 20.3 | 2.0 | -1.2 | 5.3 |
| Ecuador | 4.3 | 1.9 | 9.8 | 312 | 0.2 | 0.0 | 0.9 | 222 | 4.4 | 1.2 | 14.5 | 168 | 0.8 | 0.2 | 2.9 | 115 | 1.1 | 0.3 | 4.3 | 89 | 2.3 | -3.3 | -7.7 | 1.2 | -20.7 | -52.7 | 11.3 |
| Egypt | 8.2 | 4.8 | 13.9 | 299 | 2.7 | 1.1 | 6.3 | 263 | 8.6 | 5.6 | 12.9 | 324 | 5.5 | 3.2 | 9.3 | 314 | 4.8 | 2.5 | 9.0 | 287 | 6.2 | -1.8 | -7.2 | 3.6 | -4.1 | -18.6 | 10.5 |
| El Salvador | 6.3 | 3.2 | 12.2 | 144 | 7.7 | 3.5 | 16.3 | 104 | 5.0 | 1.5 | 15.3 | 98 | 3.3 | 1.4 | 7.5 | 86 | 5.7 | 1.1 | 24.2 | 83 | 5.7 | -2.9 | -12.3 | 6.5 | -5.2 | -30.7 | 20.4 |
| Eswatini | 4.5 | 1.0 | 17.8 | 65 | 0.6 | 0.1 | 4.6 | 59 | 1.1 | 0.1 | 7.3 | 56 | 0.0 | 0.0 | 0.0 | 30 | 0.0 | 0.0 | 0.0 | 25 | 1.5 | -5.8 | -15.2 | 3.6 | -55.3 | -87.0 | -23.6 |
| Ethiopia | 6.8 | 4.3 | 10.5 | 418 | 2.4 | 0.9 | 6.1 | 174 | 8.9 | 4.1 | 18.4 | 129 | 5.1 | 2.3 | 11.3 | 145 | 6.1 | 3.3 | 11.0 | 226 | 5.7 | 0.5 | -5.0 | 5.9 | -2.8 | -19.7 | 14.0 |
| Gabon | 6.2 | 3.7 | 10.0 | 300 | 10.0 | 3.7 | 24.2 | 137 | 21.4 | 10.1 | 40.0 | 88 | 7.0 | 1.9 | 22.1 | 60 | 7.3 | 2.6 | 19.2 | 46 | 10.8 | 2.1 | -7.9 | 12.1 | 6.0 | -9.2 | 21.2 |
| Gambia | 1.6 | 0.5 | 5.2 | 230 | 0.2 | 0.0 | 1.1 | 235 | 1.6 | 0.5 | 4.7 | 192 | 3.1 | 0.9 | 10.2 | 177 | 4.8 | 1.5 | 14.3 | 117 | 2.1 | 4.4 | -1.1 | 10.0 | 37.1 | 8.9 | 65.2 |
| Ghana | 2.0 | 0.7 | 5.5 | 210 | 5.3 | 2.3 | 12.1 | 121 | 8.2 | 3.5 | 18.0 | 112 | 10.9 | 5.5 | 20.3 | 91 | 12.9 | 5.4 | 27.8 | 72 | 7.4 | 13.9 | 2.9 | 25.0 | 25.0 | 5.9 | 44.1 |
| Guatemala | 0.4 | 0.1 | 2.5 | 318 | 0.3 | 0.0 | 2.1 | 287 | 2.3 | 0.9 | 5.8 | 222 | 5.1 | 2.4 | 10.5 | 206 | 1.4 | 0.3 | 5.4 | 142 | 1.7 | 4.3 | 1.1 | 7.5 | 41.9 | 24.4 | 59.4 |
| Guinea | 6.5 | 3.7 | 11.3 | 168 | 3.7 | 1.7 | 8.0 | 162 | 12.4 | 5.9 | 23.9 | 139 | 6.4 | 3.4 | 11.7 | 133 | 6.4 | 2.9 | 13.5 | 84 | 7.0 | 1.9 | -4.6 | 8.3 | 1.7 | -14.1 | 17.5 |
| Guinea Bissau | 3.8 | 1.9 | 7.7 | 260 | 3.7 | 1.6 | 8.2 | 209 | 3.9 | 1.6 | 9.5 | 198 | 12.1 | 5.9 | 23.1 | 94 | 20.8 | 12.5 | 32.6 | 69 | 7.5 | 18.9 | 7.8 | 29.9 | 37.9 | 19.5 | 56.2 |
| Guyana | 33.1 | 22.2 | 46.2 | 114 | 47.1 | 29.3 | 65.7 | 59 | 44.0 | 27.1 | 62.4 | 50 | 50.6 | 29.0 | 71.9 | 33 | 59.6 | 39.4 | 77.0 | 34 | 44.4 | 27.5 | 3.5 | 51.5 | 9.2 | -0.2 | 18.6 |
| Haiti | 20.0 | 13.8 | 28.0 | 200 | 13.0 | 8.2 | 20.2 | 189 | 14.7 | 8.6 | 24.0 | 141 | 21.7 | 14.6 | 30.9 | 105 | 20.6 | 11.4 | 34.5 | 65 | 17.3 | 3.0 | -9.3 | 15.3 | 2.4 | -9.4 | 14.3 |
| Honduras | 13.9 | 10.0 | 18.9 | 341 | 23.2 | 17.9 | 29.6 | 270 | 22.5 | 16.0 | 30.7 | 185 | 21.4 | 14.9 | 29.8 | 178 | 12.9 | 7.0 | 22.6 | 110 | 19.0 | 1.4 | -8.2 | 10.9 | 2.5 | -5.5 | 10.5 |
| India | 13.0 | 12.0 | 14.2 | 5829 | 13.7 | 12.6 | 15.0 | 5494 | 15.5 | 14.1 | 17.0 | 4508 | 17.8 | 15.9 | 19.9 | 3660 | 19.9 | 17.8 | 22.2 | 3135 | 15.5 | 8.2 | 5.7 | 10.8 | 9.1 | 6.5 | 11.7 |
| Indonesia | 0.8 | 0.3 | 1.7 | 510 | 1.5 | 0.7 | 3.1 | 350 | 1.9 | 0.5 | 7.5 | 281 | 3.9 | 1.7 | 8.7 | 310 | 1.6 | 0.6 | 4.1 | 235 | 1.9 | 2.3 | -0.2 | 4.8 | 19.6 | 1.5 | 37.8 |
| Iraq | 5.1 | 3.5 | 7.3 | 1320 | 5.1 | 3.1 | 8.3 | 939 | 5.4 | 3.6 | 8.1 | 691 | 8.1 | 5.1 | 12.5 | 533 | 10.5 | 6.5 | 16.5 | 399 | 6.6 | 6.3 | 1.0 | 11.6 | 15.5 | 3.5 | 27.5 |
| Jamaica | 12.8 | 4.5 | 31.1 | 28 | 2.5 | 0.5 | 10.5 | 39 | 7.4 | 2.7 | 18.6 | 47 | 6.7 | 0.9 | 35.2 | 30 | 4.5 | 1.0 | 17.4 | 23 | 6.4 | -3.5 | -17.5 | 10.4 | -7.5 | -41.1 | 26.1 |
| Jordan | 10.0 | 6.6 | 14.7 | 441 | 6.5 | 3.8 | 11.1 | 312 | 6.5 | 3.6 | 11.5 | 241 | 11.5 | 6.8 | 18.6 | 164 | 12.1 | 5.0 | 26.5 | 60 | 8.9 | 2.7 | -6.2 | 11.6 | 5.5 | -10.9 | 22.0 |
| Kazakhstan | 6.4 | 1.8 | 20.6 | 82 | 6.4 | 2.6 | 14.8 | 90 | 3.2 | 1.2 | 8.2 | 142 | 3.3 | 1.0 | 10.2 | 102 | 1.5 | 0.4 | 5.5 | 92 | 4.2 | -6.2 | -14.8 | 2.4 | -20.9 | -46.8 | 5.1 |
| Kenya | 8.0 | 5.6 | 11.3 | 670 | 10.2 | 6.7 | 15.2 | 346 | 10.8 | 6.7 | 16.7 | 281 | 4.3 | 1.4 | 12.7 | 273 | 4.4 | 2.3 | 8.5 | 240 | 7.6 | -9.4 | -19.9 | 1.0 | -7.4 | -18.7 | 3.8 |
| Kosovo | 22.3 | 10.7 | 40.8 | 38 | 5.8 | 0.8 | 32.0 | 24 | 15.1 | 4.8 | 38.6 | 28 | 11.9 | 3.7 | 32.1 | 25 | 2.8 | 0.4 | 18.5 | 30 | 12.4 | -20.3 | -41.1 | 0.5 | -22.7 | -44.1 | -1.2 |
| Kyrgyzstan | 11.9 | 6.4 | 21.1 | 89 | 13.1 | 7.0 | 23.0 | 97 | 12.0 | 5.6 | 23.8 | 93 | 10.9 | 4.5 | 24.2 | 79 | 0.0 | 0.0 | 0.0 | 74 | 9.5 | -14.0 | -23.1 | -4.9 | -21.6 | -36.9 | -6.3 |
| Lao | 2.9 | 1.0 | 7.7 | 325 | 1.7 | 0.6 | 4.6 | 273 | 1.6 | 0.6 | 4.0 | 253 | 2.8 | 1.1 | 6.8 | 175 | 5.5 | 2.5 | 11.9 | 142 | 2.7 | 2.4 | -2.6 | 7.4 | 18.2 | -8.5 | 45.0 |
| Lesotho | 2.7 | 0.7 | 9.7 | 82 | 1.7 | 0.2 | 11.3 | 76 | 0.0 | 0.0 | 0.0 | 67 | 7.3 | 1.8 | 25.3 | 59 | 12.4 | 3.4 | 36.5 | 43 | 4.4 | 11.4 | -5.1 | 27.9 | 32.4 | -6.3 | 71.0 |
| Liberia | 0.5 | 0.1 | 3.4 | 256 | 1.2 | 0.2 | 6.4 | 194 | 6.4 | 2.7 | 14.5 | 148 | 7.5 | 2.4 | 21.5 | 77 | 11.4 | 4.2 | 27.2 | 42 | 4.7 | 14.1 | 3.4 | 24.7 | 47.2 | 28.9 | 65.4 |
| Malawi | 0.7 | 0.2 | 2.2 | 379 | 1.6 | 0.7 | 3.8 | 358 | 0.8 | 0.2 | 3.1 | 317 | 0.9 | 0.2 | 3.7 | 293 | 1.2 | 0.4 | 3.8 | 289 | 1.0 | 0.2 | -1.5 | 1.9 | 2.2 | -28.0 | 32.4 |
| Maldives | 2.5 | 0.7 | 8.8 | 86 | 4.6 | 1.4 | 14.0 | 82 | 1.4 | 0.4 | 5.1 | 73 | 1.3 | 0.2 | 8.8 | 28 | 0.0 | 0.0 | 0.0 | 19 | 2.0 | -4.5 | -9.2 | 0.2 | -43.9 | -67.5 | -20.3 |
| Mali | 11.7 | 8.4 | 16.1 | 345 | 6.3 | 4.2 | 9.4 | 362 | 7.1 | 4.4 | 11.2 | 357 | 6.6 | 4.2 | 10.4 | 303 | 11.8 | 8.4 | 16.4 | 296 | 8.6 | -0.1 | -5.7 | 5.6 | -1.9 | -13.1 | 9.3 |
| Mauritania | 17.4 | 12.5 | 23.7 | 198 | 21.2 | 15.6 | 28.2 | 220 | 23.0 | 16.4 | 31.4 | 190 | 30.0 | 20.9 | 41.0 | 147 | 30.3 | 21.3 | 41.2 | 160 | 23.5 | 17.5 | 4.7 | 30.3 | 12.1 | 3.6 | 20.6 |
| Mexico | 4.4 | 1.3 | 13.6 | 189 | 9.2 | 3.9 | 20.0 | 203 | 11.7 | 3.4 | 33.5 | 134 | 5.8 | 2.7 | 12.0 | 93 | 1.6 | 0.2 | 10.9 | 47 | 7.7 | 1.1 | -9.2 | 11.4 | 14.6 | -6.6 | 35.7 |
| Moldova | 17.4 | 5.2 | 44.8 | 21 | 19.6 | 9.3 | 36.8 | 38 | 9.7 | 2.5 | 31.1 | 27 | 10.2 | 3.2 | 28.1 | 34 | 6.0 | 2.1 | 15.5 | 56 | 12.8 | -17.2 | -37.0 | 2.6 | -26.8 | -47.4 | -6.1 |
| Mongolia | 16.1 | 11.1 | 22.6 | 146 | 9.4 | 5.8 | 15.0 | 150 | 8.5 | 4.5 | 15.4 | 129 | 12.2 | 6.8 | 20.9 | 108 | 7.6 | 4.0 | 14.0 | 111 | 10.7 | -7.1 | -15.7 | 1.6 | -10.6 | -23.8 | 2.5 |
| Mozambique | 4.6 | 1.7 | 12.2 | 91 | 7.9 | 3.3 | 17.7 | 95 | 11.6 | 5.6 | 22.5 | 86 | 11.6 | 6.2 | 20.7 | 123 | 21.1 | 12.4 | 33.5 | 119 | 10.8 | 17.6 | 6.3 | 28.9 | 29.0 | 13.8 | 44.1 |
| Myanmar | 0.0 | 0.0 | 0.0 | 119 | 7.8 | 3.0 | 18.8 | 101 | 1.9 | 0.6 | 6.2 | 87 | 4.2 | 1.2 | 13.2 | 87 | 10.1 | 4.4 | 21.7 | 74 | 4.5 | 9.1 | 0.2 | 18.1 | 30.7 | 4.0 | 57.4 |
| Namibia | 4.9 | 2.0 | 11.4 | 122 | 5.7 | 2.6 | 11.9 | 124 | 13.8 | 7.3 | 24.4 | 116 | 22.5 | 13.8 | 34.6 | 97 | 21.0 | 11.9 | 34.3 | 66 | 12.3 | 24.5 | 12.4 | 36.5 | 30.5 | 18.5 | 42.4 |
| Nepal | 10.7 | 5.9 | 18.8 | 131 | 13.4 | 7.5 | 22.9 | 88 | 4.4 | 1.7 | 10.8 | 98 | 16.9 | 9.9 | 27.4 | 82 | 20.5 | 11.4 | 34.1 | 68 | 12.6 | 10.4 | -2.8 | 23.6 | 16.1 | 0.1 | 32.2 |
| Niger | 2.8 | 1.3 | 5.8 | 268 | 3.4 | 1.0 | 10.5 | 241 | 2.4 | 1.0 | 5.9 | 243 | 4.5 | 2.2 | 8.9 | 264 | 6.1 | 3.4 | 10.9 | 287 | 3.7 | 3.4 | -1.0 | 7.9 | 13.2 | -7.7 | 34.0 |
| Nigeria | 9.1 | 6.7 | 12.1 | 611 | 6.3 | 4.5 | 8.9 | 638 | 5.1 | 3.4 | 7.5 | 525 | 12.0 | 8.5 | 16.6 | 551 | 10.9 | 8.0 | 14.8 | 423 | 8.5 | 4.0 | -0.7 | 8.6 | 7.0 | -1.9 | 15.8 |
| Pakistan | 22.2 | 15.4 | 30.8 | 233 | 33.5 | 25.5 | 42.5 | 199 | 29.4 | 22.2 | 37.7 | 212 | 38.3 | 30.4 | 47.0 | 217 | 28.0 | 18.1 | 40.5 | 214 | 30.2 | 9.9 | -4.2 | 23.9 | 6.2 | -1.5 | 13.9 |
| Panama | 12.5 | 7.3 | 20.6 | 285 | 3.9 | 1.2 | 11.6 | 86 | 11.5 | 4.1 | 28.3 | 67 | 1.4 | 0.2 | 9.5 | 42 | 1.6 | 0.4 | 5.3 | 30 | 6.7 | -12.2 | -21.8 | -2.5 | -32.1 | -51.0 | -13.1 |
| Paraguay | 17.8 | 10.0 | 29.6 | 105 | 8.6 | 3.9 | 17.7 | 92 | 9.9 | 2.6 | 30.9 | 80 | 2.9 | 0.8 | 9.3 | 68 | 1.8 | 0.2 | 12.2 | 49 | 9.5 | -20.9 | -33.2 | -8.5 | -33.0 | -48.1 | -17.9 |
| Peru | 2.2 | 1.0 | 4.7 | 399 | 4.7 | 2.6 | 8.2 | 401 | 5.5 | 3.1 | 9.5 | 280 | 4.9 | 2.3 | 10.3 | 213 | 7.5 | 3.8 | 14.3 | 153 | 4.7 | 5.6 | 0.4 | 10.7 | 19.3 | 3.4 | 35.2 |
| Rwanda | 2.6 | 0.8 | 8.0 | 152 | 2.1 | 0.7 | 6.4 | 141 | 4.8 | 2.2 | 10.2 | 124 | 2.1 | 0.7 | 6.3 | 131 | 0.1 | 0.0 | 0.1 | 155 | 3.5 | 3.3 | -2.0 | 8.5 | 17.3 | -7.3 | 41.9 |
| Sao Tome and Principe | 4.0 | 0.9 | 15.9 | 44 | 0.0 | 0.0 | 0.0 | 35 | 0.0 | 0.0 | 0.0 | 40 | 0.0 | 0.0 | 0.0 | 35 | 0.1 | 0.0 | 0.5 | 15 | 2.1 | 3.2 | -11.7 | 18.2 | 23.0 | -63.9 | 109.8 |
| Senegal | 5.4 | 2.8 | 10.1 | 350 | 2.7 | 1.2 | 5.9 | 306 | 2.0 | 0.7 | 5.5 | 244 | 0.4 | 0.1 | 2.9 | 151 | 0.0 | 0.0 | 0.1 | 91 | 2.9 | -4.4 | -10.0 | 1.1 | -23.6 | -54.1 | 6.8 |
| Serbia | 24.3 | 6.1 | 61.4 | 17 | 10.4 | 3.4 | 27.9 | 23 | 5.5 | 0.7 | 31.3 | 40 | 2.4 | 0.8 | 7.3 | 39 | 0.0 | 0.0 | 0.1 | 50 | 5.7 | -16.7 | -35.8 | 2.3 | -39.1 | -72.1 | -6.2 |
| Sierra Leone | 2.2 | 0.9 | 5.0 | 246 | 4.1 | 1.1 | 14.4 | 215 | 3.1 | 1.5 | 6.4 | 266 | 2.9 | 1.2 | 7.0 | 229 | 0.1 | 0.1 | 0.2 | 159 | 4.4 | 7.4 | 1.3 | 13.5 | 25.8 | 3.0 | 48.6 |
| South Africa | 8.8 | 3.0 | 23.1 | 68 | 10.3 | 3.8 | 25.3 | 86 | 7.4 | 3.0 | 17.0 | 89 | 8.7 | 3.8 | 18.7 | 60 | 19.3 | 8.5 | 38.1 | 43 | 10.6 | 8.2 | -8.7 | 25.1 | 16.0 | -10.6 | 42.6 |
| South Sudan | 8.5 | 5.0 | 13.9 | 156 | 17.1 | 11.8 | 24.2 | 164 | 9.7 | 5.8 | 15.7 | 166 | 10.9 | 6.6 | 17.4 | 195 | 0.1 | 0.0 | 0.1 | 196 | 10.4 | -5.3 | -12.2 | 1.6 | -8.3 | -19.7 | 3.1 |
| State of Palestine | 0.0 | 0.0 | 0.0 | 154 | 1.6 | 0.4 | 6.4 | 128 | 2.3 | 0.7 | 7.2 | 121 | 2.2 | 0.8 | 5.6 | 154 | 0.1 | 0.0 | 0.1 | 108 | 2.1 | 6.0 | 1.6 | 10.4 | 42.5 | 20.2 | 64.8 |
| Sudan | 10.9 | 7.2 | 16.1 | 367 | 11.7 | 6.8 | 19.3 | 435 | 7.2 | 4.5 | 11.3 | 352 | 9.3 | 5.5 | 15.4 | 223 | 0.1 | 0.0 | 0.1 | 166 | 9.3 | -5.8 | -13.1 | 1.6 | -12.1 | -25.2 | 0.9 |
| Suriname | 39.4 | 29.8 | 49.9 | 173 | 49.2 | 32.3 | 66.3 | 38 | 40.3 | 25.6 | 57.0 | 39 | 42.2 | 25.3 | 61.1 | 36 | 0.4 | 0.2 | 0.6 | 18 | 41.3 | 1.3 | -24.6 | 27.3 | 1.6 | -7.9 | 11.0 |
| Tajikistan | 8.3 | 4.3 | 15.4 | 99 | 10.5 | 5.0 | 20.8 | 79 | 13.5 | 7.3 | 23.6 | 112 | 10.3 | 5.8 | 17.5 | 121 | 9.3 | 4.8 | 17.1 | 142 | 10.5 | 0.4 | -8.6 | 9.5 | -1.5 | -16.2 | 13.2 |
| Tanzania | 7.4 | 4.7 | 11.4 | 258 | 4.8 | 2.3 | 9.6 | 209 | 4.8 | 2.6 | 8.5 | 179 | 4.9 | 2.5 | 9.6 | 214 | 0.1 | 0.0 | 0.1 | 155 | 6.0 | -0.3 | -6.2 | 5.6 | -2.9 | -19.8 | 14.0 |
| Thailand | 11.4 | 3.1 | 34.0 | 151 | 9.6 | 3.6 | 23.3 | 153 | 9.2 | 3.6 | 21.8 | 136 | 25.2 | 13.0 | 43.2 | 130 | 0.0 | 0.0 | 0.1 | 91 | 12.6 | 4.2 | -13.6 | 22.0 | 4.8 | -18.1 | 27.7 |
| Timor Leste | 1.0 | 0.1 | 6.9 | 126 | 3.1 | 0.9 | 10.0 | 150 | 6.1 | 2.9 | 12.3 | 158 | 6.2 | 2.6 | 14.3 | 160 | 0.2 | 0.1 | 0.3 | 149 | 8.0 | 23.8 | 12.3 | 35.4 | 50.7 | 36.0 | 65.4 |
| Togo | 0.4 | 0.1 | 2.7 | 199 | 0.5 | 0.1 | 3.2 | 114 | 0.0 | 0.0 | 0.0 | 102 | 1.2 | 0.2 | 7.9 | 97 | 0.0 | 0.0 | 0.1 | 91 | 0.9 | 2.3 | -0.9 | 5.5 | 44.6 | 1.0 | 88.2 |
| Tunisia | 40.5 | 27.5 | 54.9 | 78 | 39.4 | 25.9 | 54.7 | 65 | 60.3 | 45.1 | 73.8 | 50 | 51.4 | 37.2 | 65.3 | 75 | 0.6 | 0.4 | 0.8 | 38 | 51.1 | 26.1 | 1.7 | 50.5 | 9.1 | 1.1 | 17.1 |
| Turkmenistan | 0.0 | 0.0 | 0.0 | 49 | 2.1 | 0.3 | 13.4 | 59 | 4.5 | 1.1 | 16.2 | 70 | 0.0 | 0.0 | 0.0 | 84 | 0.0 | 0.0 | 0.1 | 80 | 1.6 | 0.3 | -3.3 | 3.9 | -4.8 | -43.6 | 34.1 |
| Uganda | 4.8 | 2.7 | 8.5 | 400 | 7.3 | 4.8 | 11.0 | 331 | 9.3 | 5.8 | 14.5 | 286 | 17.6 | 12.7 | 23.8 | 255 | 0.2 | 0.1 | 0.3 | 210 | 11.0 | 19.0 | 12.4 | 25.6 | 30.1 | 21.1 | 39.0 |
| Ukraine | 18.1 | 9.4 | 31.9 | 65 | 14.8 | 6.0 | 32.1 | 76 | 11.8 | 3.5 | 32.9 | 49 | 6.4 | 2.4 | 16.1 | 57 | 0.1 | 0.0 | 0.4 | 60 | 11.9 | -9.7 | -32.5 | 13.0 | -19.9 | -52.6 | 12.8 |
| Vietnam | 9.5 | 3.7 | 22.0 | 89 | 21.0 | 11.4 | 35.5 | 68 | 23.9 | 14.3 | 37.1 | 74 | 11.1 | 5.5 | 21.2 | 68 | 0.1 | 0.1 | 0.3 | 59 | 16.2 | 0.2 | -13.9 | 14.4 | 2.9 | -11.0 | 16.8 |
| Yemen | 26.9 | 21.2 | 33.6 | 366 | 17.9 | 13.8 | 22.9 | 376 | 17.7 | 12.6 | 24.3 | 360 | 16.1 | 10.4 | 24.1 | 343 | 0.1 | 0.1 | 0.2 | 227 | 19.0 | -13.2 | -22.6 | -3.7 | -11.4 | -19.5 | -3.2 |
| Zambia | 0.0 | 0.0 | 0.0 | 288 | 1.2 | 0.4 | 3.3 | 309 | 0.3 | 0.1 | 1.1 | 251 | 0.6 | 0.1 | 2.6 | 203 | 0.0 | 0.0 | 0.1 | 138 | 0.9 | 2.5 | -0.1 | 5.1 | 46.8 | 16.7 | 76.9 |
| Zimbabwe | 0.0 | 0.0 | 0.0 | 120 | 0.0 | 0.0 | 0.0 | 117 | 0.0 | 0.0 | 0.0 | 99 | 1.7 | 0.5 | 5.6 | 161 | 0.0 | 0.0 | 0.1 | 106 | 0.9 | 4.2 | -0.2 | 8.6 | 73.1 | 61.5 | 84.7 |

^1^DHS: Demographic Health Survey; MICS: Multiple Indicator Cluster Survey; ENSANUT: Encuesta Nacional de Salud y Nutrición; ^2^SII: slope index of inequality; ^3^CIX: concentration index of inequality; ^4^CAR: Central African Republic; ^5^CDR: Congo Democratic Republic.

**Supplemental Table 9. Average weighted prevalence of consumption of non-human milk other than formula for children under 6 months of age by wealth quintiles according to regions and national income. Source: DHS, MICS, and ENSANUT, 2010-2017.1**

|  | **Wealth quintiles** | | | | |
| --- | --- | --- | --- | --- | --- |
|  | **Poorest** | **Second** | **Third** | **Fourth** | **Wealthiest** |
| **UNICEF regions** |  |  |  |  |  |
| East Asia & Pacific | 3.0 | 5.7 | 5.8 | 6.2 | 2.2 |
| South Asia | 15.1 | 17.0 | 17.1 | 20.0 | 20.3 |
| Eastern Europe & Central Asia | 11.9 | 10.5 | 8.6 | 6.1 | 2.9 |
| Middle East & North Africa | 14.8 | 10.9 | 13.4 | 13.5 | 9.0 |
| West & Central Africa | 5.7 | 4.5 | 5.1 | 8.4 | 10.0 |
| Eastern & Southern Africa | 5.3 | 5.1 | 6.8 | 6.8 | 5.8 |
| Latin America & Caribbean | 9.3 | 11.6 | 10.7 | 9.7 | 6.2 |
|  |  |  |  |  |  |
| **World Bank income groups** |  |  |  |  |  |
| Upper-middle income | 11.5 | 12.0 | 11.9 | 13.4 | 9.9 |
| Lower-middle income | 10.8 | 11.9 | 12.3 | 14.4 | 13.7 |
| Low income | 6.7 | 5.7 | 7.3 | 7.5 | 7.5 |

^1^DHS: Demographic Health Survey; MICS: Multiple Indicator Cluster Survey; ENSANUT: Encuesta Nacional de Salud y Nutrición.

**Supplemental Table 10. Percentage of infant formula consumption over all non-human milks (formula, non-human milks other than formula, or both) by wealth quintiles. Source: DHS, MICS, and ENSANUT, 2010-2017.1**

| **Country** | **Poorest** | **Second** | **Third** | **Fourth** | **Wealthiest** |
| --- | --- | --- | --- | --- | --- |
| Afghanistan | 9,3 | 18,5 | 18,7 | 25,1 | 32,9 |
| Albania | 45,9 | 58,2 | 51,7 | 61,7 | 56,0 |
| Algeria | 22,4 | 26,4 | 17,3 | 19,1 | 21,1 |
| Angola | 35,0 | 65,0 | 58,7 | 61,6 | 74,5 |
| Argentina | 63,5 | 65,3 | 96,0 | 83,3 | 95,3 |
| Armenia | 30,0 | 21,0 | 15,8 | 25,5 | 6,7 |
| Bangladesh | 45,0 | 26,1 | 47,2 | 74,5 | 67,1 |
| Belarus | 85,1 | 91,6 | 100,0 | 96,4 | 91,5 |
| Belize | 70,2 | 68,5 | 84,2 | 90,6 | 81,0 |
| Benin | 5,8 | 0,0 | 26,9 | 30,6 | 32,4 |
| Bhutan | 22,9 | 37,5 | 42,3 | 72,3 | 80,1 |
| Burkina Faso | 0,0 | 0,0 | 46,1 | 0,0 | 74,4 |
| Burundi | 50,0 | 0,0 | 0,0 | 0,0 | 29,1 |
| Cambodia | 0,0 | 43,5 | 56,5 | 81,3 | 67,8 |
| Cameroon | 92,5 | 98,3 | 86,7 | 71,7 | 61,0 |
| Central African Republic | 59,6 | 72,2 | 67,0 | 79,1 | 81,9 |
| Chad | 13,3 | 3,1 | 5,9 | 7,3 | 53,8 |
| Comoros | 65,4 | 56,8 | 66,1 | 72,9 | 61,3 |
| Congo Brazzaville | 46,9 | 41,6 | 40,5 | 36,4 | 48,3 |
| Congo Democratic Republic | 19,2 | 2,0 | 17,0 | 54,8 | 63,6 |
| Costa Rica | 65,6 | 68,4 | 95,2 | 93,2 | 68,4 |
| Cote d’Ivoire | 5,8 | 52,7 | 41,6 | 45,0 | 59,4 |
| Dominican Republic | 28,5 | 29,7 | 39,0 | 31,4 | 40,8 |
| Egypt | 65,1 | 82,0 | 62,7 | 74,5 | 81,5 |
| El Salvador | 74,7 | 80,4 | 87,5 | 92,1 | 87,3 |
| Eswatini | 54,9 | 90,9 | 94,7 | 100,0 | 100,0 |
| Ethiopia | 2,6 | 8,3 | 1,0 | 1,1 | 46,6 |
| Gabon | 87,1 | 86,9 | 77,4 | 90,3 | 92,0 |
| Gambia | 25,6 | 50,0 | 35,8 | 64,9 | 44,4 |
| Ghana | 0,0 | 49,7 | 57,2 | 58,9 | 62,1 |
| Guatemala | 90,3 | 96,4 | 89,8 | 88,7 | 97,6 |
| Guinea | 0,0 | 47,7 | 31,4 | 61,0 | 77,2 |
| Guinea Bissau | 18,3 | 13,0 | 10,4 | 13,2 | 24,9 |
| Guyana | 36,8 | 52,8 | 55,2 | 53,8 | 51,8 |
| Haiti | 28,7 | 50,6 | 54,6 | 54,6 | 68,9 |
| Honduras | 29,5 | 39,2 | 59,7 | 64,9 | 78,2 |
| India | 13,4 | 20,7 | 20,1 | 20,1 | 21,3 |
| Indonesia | 96,1 | 95,4 | 93,9 | 92,1 | 95,6 |
| Iraq | 86,2 | 89,2 | 88,8 | 85,8 | 84,4 |
| Jamaica | 74,4 | 92,6 | 90,3 | 89,9 | 93,6 |
| Jordan | 80,0 | 88,1 | 89,5 | 82,4 | 82,1 |
| **Country** | **Poorest** | **Second** | **Third** | **Fourth** | **Wealthiest** |
| Kazakhstan | 69,1 | 79,7 | 87,7 | 79,4 | 93,9 |
| Kenya | 9,1 | 0,0 | 7,5 | 15,5 | 10,7 |
| Kosovo | 48,7 | 76,6 | 59,9 | 76,4 | 87,8 |
| Kyrgyzstan | 23,5 | 27,1 | 38,1 | 56,0 | 100,0 |
| Lao | 46,9 | 64,8 | 84,4 | 83,7 | 83,2 |
| Lesotho | 61,8 | 84,4 | 100,0 | 76,3 | 79,9 |
| Liberia | 65,1 | 23,9 | 48,9 | 56,8 | 52,4 |
| Malawi | 78,1 | 35,0 | 47,7 | 75,4 | 69,9 |
| Maldives | 89,7 | 73,8 | 83,2 | 92,5 | 100,0 |
| Mali | 4,7 | 10,2 | 7,1 | 25,1 | 26,0 |
| Mauritania | 12,5 | 14,8 | 20,0 | 38,3 | 50,5 |
| Mexico | 90,2 | 84,4 | 77,7 | 91,6 | 97,7 |
| Moldova | 21,1 | 49,9 | 74,1 | 70,5 | 81,7 |
| Mongolia | 17,1 | 45,0 | 68,5 | 69,9 | 81,2 |
| Myanmar | 100,0 | 58,3 | 68,5 | 48,1 | 53,6 |
| Namibia | 58,2 | 54,6 | 44,5 | 56,6 | 62,8 |
| Nepal | 8,1 | 20,0 | 0,0 | 24,1 | 43,4 |
| Niger | 6,0 | 6,8 | 0,0 | 17,2 | 48,0 |
| Nigeria | 7,9 | 29,0 | 50,6 | 48,9 | 58,1 |
| Pakistan | 16,0 | 11,9 | 22,6 | 18,5 | 48,9 |
| Panama | 60,1 | 92,0 | 87,5 | 98,1 | 98,1 |
| Paraguay | 43,5 | 73,0 | 76,3 | 94,0 | 96,0 |
| Peru | 61,4 | 75,4 | 80,0 | 85,6 | 83,7 |
| Rwanda | 0,0 | 0,0 | 17,4 | 0,0 | 97,3 |
| Sao Tome and Principe | 37,5 | 100,0 | 100,0 | 100,0 | 98,2 |
| Senegal | 18,3 | 33,7 | 79,2 | 93,0 | 99,8 |
| Serbia | 49,2 | 83,9 | 85,9 | 96,2 | 99,9 |
| Sierra Leone | 35,2 | 40,0 | 58,2 | 67,6 | 99,4 |
| South Africa | 77,5 | 69,7 | 84,7 | 77,1 | 62,3 |
| South Sudan | 39,1 | 29,1 | 28,7 | 47,7 | 99,5 |
| State of Palestine | 100,0 | 96,5 | 94,0 | 95,0 | 99,8 |
| Sudan | 19,2 | 35,2 | 20,3 | 10,6 | 99,1 |
| Suriname | 63,0 | 61,2 | 66,9 | 64,3 | 99,5 |
| Tajikistan | 43,5 | 34,0 | 44,6 | 52,7 | 57,6 |
| Tanzania | 0,0 | 1,8 | 0,0 | 6,5 | 98,0 |
| Thailand | 76,7 | 80,8 | 81,7 | 70,8 | 100,0 |
| Timor Leste | 68,0 | 56,1 | 60,0 | 66,2 | 98,8 |
| Togo | 0,0 | 73,2 | 100,0 | 62,3 | 99,6 |
| Tunisia | 20,5 | 8,6 | 12,3 | 16,1 | 95,9 |
| Turkmenistan | 100,0 | 75,5 | 73,8 | 100,0 | 99,9 |
| Uganda | 0,8 | 0,8 | 4,0 | 3,6 | 89,8 |
| Ukraine | 59,1 | 66,8 | 61,8 | 86,5 | 99,7 |
| Vietnam | 59,3 | 63,3 | 62,5 | 82,2 | 99,7 |
| Yemen | 43,7 | 62,6 | 65,4 | 67,7 | 99,7 |
| **Country** | **Poorest** | **Second** | **Third** | **Fourth** | **Wealthiest** |
| Zambia | 100,0 | 0,0 | 47,2 | 73,8 | 99,6 |
| Zimbabwe | 100,0 | 100,0 | 100,0 | 74,3 | 99,7 |
| **Mean** | 44,3 | 49,9 | 55,2 | 59,9 | 74,0 |

^1^DHS: Demographic Health Survey; MICS: Multiple Indicator Cluster Survey; ENSANUT: Encuesta Nacional de Salud y Nutrición.
